# Gold(I)‐Catalyzed Haloalkynylation of Aryl Alkynes: Two Pathways, One Goal

**DOI:** 10.1002/anie.201916027

**Published:** 2020-04-06

**Authors:** Mathis Kreuzahler, Gebhard Haberhauer

**Affiliations:** ^1^ Institut für Organische Chemie Universität Duisburg-Essen Universitätsstraße 7 45117 Essen Germany

**Keywords:** C−C bond formation, DFT calculations, enynes, gold, vinyl cations

## Abstract

Haloalkynylation reactions provide an efficient method for the simultaneous introduction of a halogen atom and an acetylenic unit. For the first time, we report a gold(I)‐catalyzed haloalkynylation of aryl alkynes that delivers exclusively the *cis* addition product. This method enables the simple synthesis of conjugated and halogenated enynes in yields of up to 90 %. Notably, quantum chemical calculations reveal an exceptional interplay between the place of the attack at the chloroacetylene: No matter which C−C bond is formed, the same enyne product is always formed. This is only possible through rearrangement of the corresponding skeleton. Hereby, one reaction pathway proceeds via a chloronium ion with a subsequent aryl shift; in the second case the corresponding vinyl cation is stabilized by a 1,3‐chlorine shift. ^13^C‐labeling experiments confirmed that the reaction proceeds through both reaction pathways.

## Graphical abstract

**One way or another**: A gold(I)‐catalyzed haloalkynylation of aryl alkynes was developed that delivers exclusively the *cis* addition product. This method enables the synthesis of conjugated and halogenated enynes in yields of up to 90 % yield. Notably, attack at the chloroacetylene can occur at either of two carbon atoms, but owing to rearrangement of the corresponding skeleton, the same enyne product is always formed.

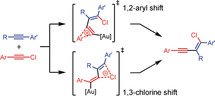

The development of novel and highly efficient carbon–carbon bond‐forming reactions for the design of complex molecules is a fundamental goal in organic chemistry.^1^ One of the most important substrate classes is halogenated compounds. Since the halogen atom is usually discarded in the course of carbon–carbon bond‐forming reactions, the development of C−C bond‐forming reactions in which the halogen atom remains in the product, is of great interest. So far, only a few examples have been reported; some of these reactions start from haloacetylenes.^2^ The latter are readily accessible2b and decompose, with the exception of fluoroacetylenes,^3^ only at higher temperatures.^4^ Until recently, the simultaneous addition of one halogen atom and one alkyne unit (haloalkynylation) to a carbon–carbon double bond was only possible for norbornene systems.^5^ We were able to demonstrate for the first time that the chloroalkynylation of 1,1‐disubstituted alkenes **2** can be achieved through gold(I) catalysis^6^ leading to the homopropargyl chlorides **3** in good yields (Scheme 1 a).^7^ This reaction principle can also be extended to bromoacetylenes **4** and 1,2‐disubstituted alkenes (**5**; Scheme 1 b)^8^ and represents one of the few examples for gold(I)‐catalyzed reactions where the triple bond remains after the reaction.^9^, ^10^ In the case of the gold(I)‐catalyzed haloalkynylation of cyclic alkenes, a side reaction, namely the already known gold(I)‐catalyzed [2+2] cycloaddition,^11^ takes place (Scheme 1 b).^8^ The bromoalkynylation of cyclic alkenes proceeds via a *trans* addition and can also be accomplished enantioselectively by the use of chiral gold(I) catalysts.^12^

**Scheme 1:**
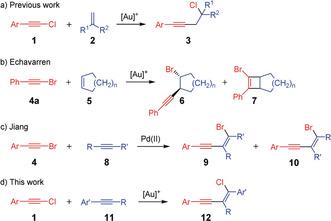
Gold(I)‐catalyzed haloalkynylation of 1,1‐disubstituted (a) and 1,2‐disubstituted (b) alkenes. Palladium(II)‐ and gold(I)‐catalyzed addition of haloarylacetylenes **4** (c) and **1** (d) to internal alkynes.

The gold‐catalyzed haloalkynylation of internal alkynes has not been described so far; only a palladium‐catalyzed variant exists.^13^ However, the application of this method is primarily restricted to hydroxyalkyl‐substituted triple bonds.13a In case of dialkyl‐ and arylalkyl alkynes, both regioisomers (conjugated and cross‐conjugated) are always formed in almost equal ratio (Scheme 1 c).13a In this work, we studied the haloalkynylation of aryl alkynes through gold catalysis, and we demonstrate that this reaction leads to the highly selective formation of the conjugated and halogenated enynes in yields up to 90 % (Scheme 1 d). Mechanistic investigations revealed that the product is formed through two extraordinary and complementary reaction pathways.

For investigation of the haloalkynylation reaction, chloroarylacetylene **1 a** was chosen as model system (Table 1) in the first step. The electronegative fluorine atom, which is attached to the *para* position of the aryl alkyne unit, should slow down the competing dimerization^10^ of **1 a**. Alkyne **11 a** was chosen as simple representative for the aryl alkynes. Dry 1,2‐dichloroethane (DCE) was used as reaction solvent. The use of an appropriate concentration of the chloroarylacetylene **1 a** is essential since higher concentrations accelerate the dimerization,^10^ whereas lower concentrations favor the hydration of the chloroarylacetylene.^14^ The first attempt with a ratio of 1:1 for the starting materials (**1 a** and **11 a**) and 5 mol % [JohnPhos(AuNCMe)]SbF_6_,^15^ which gave good results for the previously reported chloroalkynylation of 1,1‐disubstitued alkenes (see Scheme 1 a),^7^ led to a single product with a yield of 56 % (entry 1 in Table 1). The ^1^H NMR spectrum of the crude product shows no significant formation of byproducts. An analysis by one‐ and two‐dimensional NMR spectroscopy indicates that the reaction product is the conjugated *cis*‐addition product **12 a** (Figures S16–S17).

**Table 1:** Optimization of the reaction conditions for the gold(I)‐catalyzed chloroalkynylation of alkyne **11 a**.^[a]^ 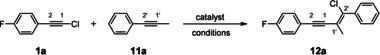

Entry	**1 a** : **11 a**	Catalyst	Yield [%]
1	1:1	[JohnPhosAu(NCMe)]SbF_6_ (5 mol %)^15^	56
2	1:1.5	[JohnPhosAu(NCMe)]SbF_6_ (5 mol %)	66
3	1:1.5	JohnPhosAuNTf_2_ (5 mol %)^16^	67
4	1:1.5	CyJohnPhosAuCl (5 mol %), NaBArF_24_ (5 mol %)^17^	56
5	1:1.5	CyJohnPhosAuCl (5 mol %), AgSbF_6_ (5 mol %)	67
6	1:1.5	CyJohnPhosAuCl (5 mol %), AgNTf_2_ (5 mol %)	65
7	1:1.5	*t*BuXPhosAu(NCMe)SbF_6_ 18	53
8	1:1.5	*t*BuXPhosAuNTf_2_ 16	41
9	1:1.5	*t*BuXPhosAuCl (5 mol %), NaBArF_24_ (7 mol %)^16^	59
10	1:1.5	BrettPhosAuNTf_2_ (5 mol %)^19^	29
11	1:1.5	XPhosAu(NCMe)SbF_6_ (5 mol %)^20^	49
12	1:1.5	Dichloro(2‐picolinato)gold(III)^21^	0
13	1:1.5	IPrAuNTf_2_ 16	23
14	1:1.5	IPrAuCl (5 mol %), AgSbF_6_ (7 mol %)^22^	25
15^b^	1:1.5	[JohnPhosAu(NCMe)]SbF_6_ (5 mol %)	65
16^c^	1:1.5	[JohnPhosAu(NCMe)]SbF_6_ (5 mol %)	57
17	1.5:1	[JohnPhosAu(NCMe)]SbF_6_ (5 mol %)	66
18	1:2	[JohnPhosAu(NCMe)]SbF_6_ (5 mol %)	70
19	1:3	[JohnPhosAu(NCMe)]SbF_6_ (5 mol %)	66
20	1:2	Me_3_PAuCl (5 mol %), AgSbF_6_ (10 mol %)	64

[a] The yield for **12 a** was determined by ^1^H NMR spectroscopy using hexamethylbenzene as internal standard. The reaction was performed in dry 1,2‐dichloroethane (DCE) at room temperature. Unless stated otherwise, the concentration was 0.1 m for **1 a**. [b] 0.05 m for **1 a**. [c] 0.2 m for **1 a**.

Raising the equivalents of **11 a** to 1.5 increased the yield of **12 a**, so this ratio was initially kept for further catalyst screening (entry 2). Starting from JohnPhos‐type ligands, we first varied the counterion^23^ as well as the other substituents attached to the phosphor atom of the phosphine (entries 3–6). The yields barely changed and were in the range of 56 to 67 %. The use of sterically more demanding phosphine ligands, like XPhos and BrettPhos, led to a strong decrease in the yield (entries 7–11). The use of the gold(III) complex dichloro(2‐pyridinecarboxylato)gold did not give any addition product at all (entry 12). When using N‐heterocyclic carbene ligand^22^ complexes with different counterions, the product was only formed in low yields (entry 13 and 14). Lowering the concentration of the starting materials resulted in no significant change in the yield, although the reaction time noticeably increased (entry 15). Increasing the concentration was accompanied by a decrease in the reaction yield (entry 16). A post‐optimization of the ratio of **1 a** and **11** (entries 17–19) showed that the ideal ratio of **1 a** and **11** is 1:2 (entry 18, see Figure S7).

In the second part, we performed the reaction on a preparative scale (0.4 mmol) in order to evaluate the scope of the chloroalkynylation (Scheme 2) under the optimized reaction conditions (entry 18 in Table 1). The size of the alkyl chain of alkyne **11** has no significant impact on the yield (**12 a** to **12 c**). When the alkyne **11** bears a substituent at the *para* position of the aryl unit, the yield increases only with electron‐donating substituents (**12 d** to **12 f**) which gave yields up to 90 % (**12 f**). In the case of electron‐withdrawing substituents attached to the *para* position of the alkyne **11**, for example, for **11 e**, no selective formation of the corresponding enyne product could be observed. The substitution pattern (*para* vs. *ortho*) of the aromatic unit of the chloroarylacetylene is not important since the yields for **12 g** and **12 h** are almost the same. By contrast, the electronic nature of the substituent attached to the aromatic unit of the chloroarylacetylene **1** is crucial: Chloroarylacetylenes **1** with electron‐withdrawing substituents lead to high yields of the corresponding enynes **12**, whereas electron‐donating groups decrease the yield (**12 f** to **12 j**). For chloroarylacetylenes with strong electron‐donating groups, for example, for chloroarylacetylene **1 f**, an unselective reaction was observed that delivered the enyne product in significant lower yields (<20 %).

**Scheme 2:**
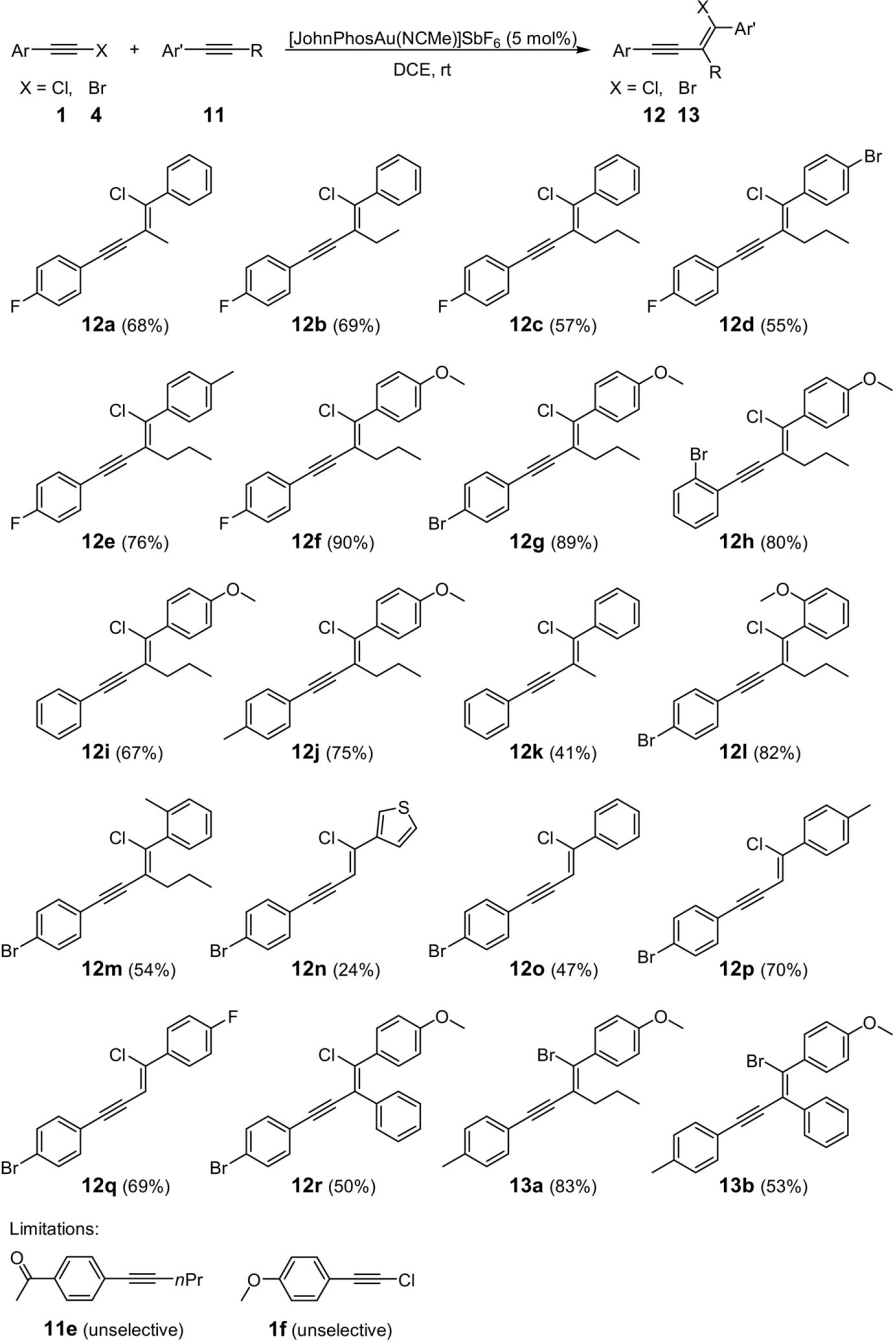
Evaluation of the substrate scope of the gold(I)‐catalyzed haloalkynylation of aryl alkynes **11**.

To our delight, the scope of the reaction could be extended to both terminal aryl alkynes and diaryl alkynes. Here again, only one regioisomer was obtained (**12 n** to **12 r**). Furthermore, the analogous reaction of bromoarylacetylenes **4** led to similar yields (**13 a** and **13 b**).

In the third step, we wanted to gain an insight into the reaction mechanism. As a model reaction, we chose the gold(I)‐catalyzed reaction of phenylchloroacetylene with 1‐phenyl‐1‐propyne (**11 a**) with both Me_3_P and JohnPhos as ligands of the gold catalyst (Scheme 3). For the addition of the alkyne **11 a** to the gold complex **14**, two realistic reaction pathways were considered, namely addition to the C2 (route A) or C1 (route B) positions of complex **14**. Both reaction pathways lead to product **20**, which corresponds to the gold(I) complex of the successfully isolated enyne **12 k** (Scheme 2). Route A starts with addition to the C2 carbon atom of gold complex **14** and proceeds via the vinyl cation **16** to give the chloronium ion **18**. Subsequent shift of the aryl group leads to the gold complex **20**, in which the carbon atom that was initially attached to the chlorine atom is now directly bound to the aromatic unit. A mechanism involving a bromonium cyclic intermediate similar to **18** has been proposed for the bromoalkynylation of 1,2‐disubstituted alkenes.^8^ Route B starts with addition to the C1 carbon atom of gold complex **14** to form the vinyl cation **22**. After rotation around the C1−C1′ axis, the vinyl cation **24** is formed, which can be stabilized through a 1,3‐chlorine shift to give complex **20**. The carbon atom that was formerly attached to the chlorine atom is now connected to the alkenyl unit.

**Scheme 3:**
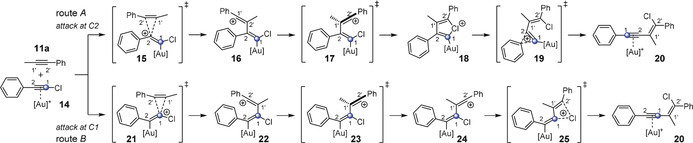
The gold(I)‐catalyzed 1,2‐chloroalkynylation of alkyne **11 a** can proceed through attack at either the C2 (route A) or C1 (route B) carbon atoms of the alkyne complex **14**.

To examine which of these reaction pathways (route A and route B, Scheme 3) the haloalkynylation reaction proceeds through, the reaction of alkyne **11 a** with gold complex **14** was calculated by means of DFT calculations (B3LYP,^24^ PBE0,^26^ M06‐2X^27^ and B97‐D^28^) with dispersion corrections^25^ and different basis sets (see the Supporting Information). The calculated data are summarized in Tables S1 and S2 as well as Figure 1 and Figure S13.

**Figure 1:**
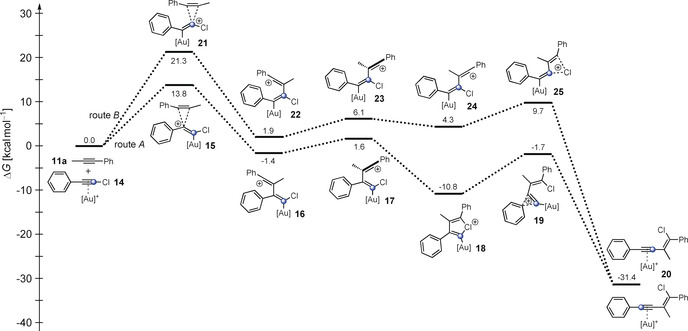
Free‐energy (Δ*G*) profile for the gold(I)‐catalyzed 1,2‐haloalkynylation of alkyne **11 a** through attack at the C2 (route A) or C1 (route B) carbon atoms of alkyne complex **14**, respectively, calculated by means of B3LYP‐D3BJ(SMD). [Au]^+^=JohnPhosAu^+^.

Let us consider the values obtained by B3LYP (B3LYP‐D3BJ(dichloroethane as solvent)/B3//B3LYP‐D3BJ/B1) with JohnPhos as ligand of the gold catalyst for both reaction pathways (route A and B in Scheme 3). It becomes obvious that in either case, the rate‐determining step is the first one, that is, the addition of the alkyne **11 a** to the complex **14** (Figure 1). With this level of theory, the activation barrier for route A amounts to 13.8 kcal mol^−1^. The intermediate **16** (route A) can be stabilized through rotation around the C2−C2′ single bond to form chloronium ion **18** (Δ*G*=−9.4 kcal mol^−1^; for numbering see Scheme 3). The activation barrier for the subsequent aryl shift exhibits a value of 9.1 kcal mol^−1^. However, the activation barrier for the rate‐determining step of route B amounts to 21.3 kcal mol^−1^ and is therefore significantly higher than that for route A (13.8 kcal mol^−1^; Figure 1). The thus formed vinyl cation **22** can now merge into the conformer **24** by rotation. In contrast to route A, the rotation leads to no stabilization (Δ*G*=+2.4 kcal mol^−1^). The final step is the formation of gold complex **20** through a 1,3‐chlorine shift, which has a slightly lower activation barrier (5.4 kcal mol^−1^) than the rearrangement of the aryl group for route A (9.1 kcal mol^−1^; Figure 1).

Additionally, all other density functionals (PBE0, M06‐2X and B97‐D; Table S1) predict that route A is energetically favored compared to route B. Thus, all calculations forecast the preferred addition of alkyne **11 a** to the C2 carbon atom of **14** followed by a 1,2‐aryl shift (route A; for numbering see Scheme 3).

To verify our calculations, we attempted to confirm the previously proposed reaction mechanism. We assumed that ^13^C‐labeling of one of the starting materials should help in gaining mechanistic insights. Therefore, we synthesized a chlorophenylacetylene in which the outer acetylenic carbon atom is ^13^C‐labeled (^13^C(1)‐**1 d**; Scheme 4). The gold(I)‐catalyzed chloroalkynylation of the aryl alkyne **11 f** delivered the enyne product ^13^C‐**12 i** with a total yield of 81 %. A closer look at the ^13^C NMR spectrum reveals that the ^13^C signals for both acetylenic carbon atoms C1 and C2 are enriched with the carbon isotope ^13^C (Figures S14 and S15). According to the quantitative ^13^C NMR spectrum, the percentage of ^13^C is 14 and 98 times, respectively, higher at positions C1 and C2 than that for the quaternary carbon atom C5′ which just shows the natural abundance of the carbon isotope ^13^C (ca. 1 %) (Figure S15). Since both intensive signals of the acetylenic carbon atoms (C1 and C2) exhibit no splitting pattern (one would expect a doublet corresponding to the ^1^ *J* coupling of both ^13^C‐labeled acetylenic carbon atoms), the ^13^C‐enriched carbon atoms (C1 and C2) cannot be present in the same molecule. Therefore, the isolated enyne must be a mixture of compounds ^13^C(2)‐**12 i** and ^13^C(1)‐**12 i** (Scheme 4). The ratio of compounds ^13^C(2)‐**12 i** and ^13^C(1)‐**12 i** was determined using the integrals for the ^13^C‐enriched signals of C2 and C1, respectively, and amounts to 87:13 (Figure S15). This demonstrates that the reaction proceeds through both reaction pathways (route A and B in Figure 1), but the pathway via the chloronium ion **18** (route A) is favored.

**Scheme 4:**
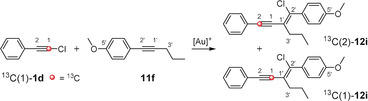
Investigation of the reaction mechanism of the gold(I)‐catalyzed chloroalkynylation of **11 f** by using ^13^C‐labeled **1 d**.

In conclusion, we have developed a gold(I)‐catalyzed variant of the haloalkynylation reaction that gives direct access to conjugated and halogenated enynes with good to very good yields (up to 90 %) from readily available starting materials, while tolerating a broad range of substrates with respect to both alkyne reactants. Since the halogen pattern on both the aromatic and vinylic unit enables potential further transformations (see Scheme S1), the gold(I)‐catalyzed haloalkynylation of aryl alkynes represents an attractive method for the synthesis of more complex conjugated systems. Of particular interest is the fact that the enyne product can be formed through two complementary pathways: The regioselectivity of the C−C bond formation plays absolutely no role since rearrangement of the skeleton results in the same product. ^13^C‐labeling experiments show that the reaction indeed passes through both ways. This interplay could be employed for the development of future novel carbon–carbon bond‐forming reactions.

## Conflict of interest

The authors declare no conflict of interest.

## Supporting information

As a service to our authors and readers, this journal provides supporting information supplied by the authors. Such materials are peer reviewed and may be re‐organized for online delivery, but are not copy‐edited or typeset. Technical support issues arising from supporting information (other than missing files) should be addressed to the authors.

Supplementary

## References

[1] R. C. Larock , Comprehensive Organic Transformations: A Guide to Functional Group Preparations, VCH Publishers, Weinheim, 1989.

[2a] D. A. Petrone , J. Ye , M. Lautens , Chem. Rev. 2016, 116, 8003–8104; 27341176 10.1021/acs.chemrev.6b00089

[2b] W. Wu , H. Jiang , Acc. Chem. Res. 2014, 47, 2483–2504. 24985140 10.1021/ar5001499

[3] H. G. Viehe , R. Merényi , J. F. M. Oth , P. Valange , Angew. Chem. Int. Ed. Engl. 1964, 3, 746;

[4a] A. Janiszewski , J. Fax , G. Haberhauer , Org. Chem. Front. 2019, 6, 1010–1021;

[4b] S. Fabig , A. Janiszewski , M. Floß , M. Kreuzahler , G. Haberhauer , J. Org. Chem. 2018, 83, 7878–7885; 29842787 10.1021/acs.joc.8b00755

[4c] S. Fabig , G. Haberhauer , R. Gleiter , J. Am. Chem. Soc. 2015, 137, 1833–1843. 25597255 10.1021/ja510699b

[5] Y. Li , X. Liu , H. Jiang , B. Liu , Z. Chen , P. Zhou , Angew. Chem. Int. Ed. 2011, 50, 6341–6345; 10.1002/anie.201100002 21630395

[6a] L. Liu , J. Zhang , Chem. Soc. Rev. 2016, 45, 506–516; 26658761 10.1039/c5cs00821b

[6b] D. Pflästerer , A. S. K. Hashmi , Chem. Soc. Rev. 2016, 45, 1331–1367; 26673389 10.1039/c5cs00721f

[6c] R. Dorel , A. M. Echavarren , Chem. Rev. 2015, 115, 9028–9072; 25844920 10.1021/cr500691k PMC4580024

[6d] D. Qian , J. Zhang , Chem. Soc. Rev. 2015, 44, 677–698; 25522173 10.1039/c4cs00304g

[6e] J. Xie , C. Pan , A. Abdukader , C. Zhu , Chem. Soc. Rev. 2014, 43, 5245–5256; 24853478 10.1039/c4cs00004h

[6f] L. Zhang , Acc. Chem. Res. 2014, 47, 877–888; 24428596 10.1021/ar400181x PMC3983127

[6g] M. Rudolph , A. S. K. Hashmi , Chem. Soc. Rev. 2012, 41, 2448–2462; 22182942 10.1039/c1cs15279c

[6h] M. Bandini , Chem. Soc. Rev. 2011, 40, 1358–1367; 21103507 10.1039/c0cs00041h

[6i] A. Corma , A. Leyva-Pérez , M. J. Sabater , Chem. Rev. 2011, 111, 1657–1712; 21391565 10.1021/cr100414u

[6j] A. Fürstner , Chem. Soc. Rev. 2009, 38, 3208–3221; 19847352 10.1039/b816696j

[6k] A. Arcadi , Chem. Rev. 2008, 108, 3266–3325; 18651778 10.1021/cr068435d

[6l] A. Fürstner , P. W. Davies , Angew. Chem. Int. Ed. 2007, 46, 3410–3449; 10.1002/anie.200604335 17427893

[6m] A. S. K. Hashmi , G. J. Hutchings , Angew. Chem. Int. Ed. 2006, 45, 7896–7936; 10.1002/anie.200602454 17131371

[6n] A. Hoffmann-Röder , N. Krause , Org. Biomol. Chem. 2005, 3, 387–391. 15678171 10.1039/b416516k

[7] M. Kreuzahler , G. Haberhauer , J. Org. Chem. 2019, 84, 8210–8224. 31192596 10.1021/acs.joc.9b01371

[8] M. E. de Orbe , M. Zanini , O. Quinonero , A. M. Echavarren , ACS Catal. 2019, 9, 7817–7822.

[9a] S. Mader , L. Molinari , M. Rudolph , F. Rominger , A. S. K. Hashmi , Chem. Eur. J. 2015, 21, 3910–3913; 25620322 10.1002/chem.201406594

[9b] Y. Yu , W. Yang , D. Pflästerer , A. S. K. Hashmi , Angew. Chem. Int. Ed. 2014, 53, 1144–1147; 10.1002/anie.201307647 24338996

[9c] A. S. K. Hashmi , W. Yang , Y. Yu , M. M. Hansmann , M. Rudolph , F. Rominger , Angew. Chem. Int. Ed. 2013, 52, 1329–1332; 10.1002/anie.201207287 23212940

[10] M. Kreuzahler , A. Daniels , C. Wölper , G. Haberhauer , J. Am. Chem. Soc. 2019, 141, 1337–1348. 30588811 10.1021/jacs.8b11501

[11] Y.-B. Bai , Z. Luo , Y. Wang , J.-M. Gao , L. Zhang , J. Am. Chem. Soc. 2018, 140, 5860–5865. 29618202 10.1021/jacs.8b01813 PMC6488029

[12] P. García-Fernández , C. Izquierdo , J. Iglesias-Sigüenza , E. Díez , R. Fernández , J. M. Lassaletta , Chem. Eur. J. 2020, 26, 629–633. 31702073 10.1002/chem.201905078

[13a] Y. Li , X. Liu , H. Jiang , Z. Feng , Angew. Chem. Int. Ed. 2010, 49, 3338–3341; 10.1002/anie.201000003 20340147

[13b] T. Wada , M. Iwasaki , A. Kondoh , H. Yorimitsu , K. Oshima , Chem. Eur. J. 2010, 16, 10671–10674. 20690120 10.1002/chem.201000865

[14] L. Xie , Y. Wu , W. Yi , L. Zhu , J. Xiang , W. He , J. Org. Chem. 2013, 78, 9190–9195. 23952293 10.1021/jo401437w

[15] C. Nieto-Oberhuber , M. P. Muñoz , S. López , E. Jiménez-Núñez , C. Nevado , E. Herrero-Gómez , M. Raducan , A. M. Echavarren , Chem. Eur. J. 2006, 12, 1677–1693. 16358351 10.1002/chem.200501088

[16] C. Fehr , M. Vuagnoux , A. Buzas , J. Arpagaus , H. Sommer , Chem. Eur. J. 2011, 17, 6214–6220. 21503982 10.1002/chem.201002797

[17] C. Nieto-Oberhuber , S. López , A. M. Echavarren , J. Am. Chem. Soc. 2005, 127, 6178–6179. 15853316 10.1021/ja042257t

[18] N. Sun , X. Xie , H. Chen , Y. Liu , Chem. Eur. J. 2016, 22, 14175–14180. 27535212 10.1002/chem.201603055

[19] L. Ye , W. He , L. Zhang , Angew. Chem. Int. Ed. 2011, 50, 3236–3239; 10.1002/anie.201007624 PMC3167741 21381165

[20] V. López-Carrillo , A. M. Echavarren , J. Am. Chem. Soc. 2010, 132, 9292–9294. 20568750 10.1021/ja104177w

[21] A. S. K. Hashmi , J. P. Weyrauch , M. Rudolph , E. Kurpejović , Angew. Chem. Int. Ed. 2004, 43, 6545–6547; 10.1002/anie.200460232 15578764

[22] P. de Frémont , N. M. Scott , E. D. Stevens , S. P. Nolan , Organometallics 2005, 24, 2411–2418.

[23a] J. Schießl , J. Schulmeister , A. Doppiu , E. Wörner , M. Rudolph , R. Karch , A. S. K. Hashmi , Adv. Synth. Catal. 2018, 360, 2493–2502;

[23b] J. Schießl , J. Schulmeister , A. Doppiu , E. Wörner , M. Rudolph , R. Karch , A. S. K. Hashmi , Adv. Synth. Catal. 2018, 360, 3949–3959.

[24a] B. Miehlich , A. Savin , H. Stoll , H. Preuss , Chem. Phys. Lett. 1989, 157, 200–206;

[24b] A. D. Becke , Phys. Rev. A 1988, 38, 3098–3100; 10.1103/physreva.38.3098 9900728

[24c] C. Lee , W. Yang , R. G. Parr , Phys. Rev. B 1988, 37, 785–789. 10.1103/physrevb.37.785 9944570

[25] S. Grimme , S. Ehrlich , L. Goerigk , J. Comput. Chem. 2011, 32, 1456–1465. 21370243 10.1002/jcc.21759

[26a] C. Adamo , V. Barone , J. Chem. Phys. 1999, 110, 6158–6170;

[26b] M. Ernzerhof , G. E. Scuseria , J. Chem. Phys. 1999, 110, 5029–5036.

[27] Y. Zhao , D. G. Truhlar , Theor. Chem. Acc. 2008, 120, 215–241.

[28] S. Grimme , J. Comput. Chem. 2006, 27, 1787–1799. 16955487 10.1002/jcc.20495

