# Supplementary material for: Gold(I)‐Catalyzed Haloalkynylation of Aryl Alkynes: Two Pathways, One Goal
Source: Angew Chem Int Ed Engl. 2020 Apr 6;59(24):9433–7. doi: 10.1002/anie.201916027 (PMC7318269; doi:10.1002/anie.201916027)
Supplement: Supplementary file 1 — Supplementary [file ANIE-59-9433-s001.pdf]

Angewandte Chemie
Eine Zeitschrift der Gesellschaft Deutscher Chemiker

Supporting Information

Gold(I)-Catalyzed Haloalkynylation of Aryl Alkynes: Two Pathways, One Goal

*Mathis Kreuzahler and Gebhard Haberhauer**

anie_201916027_sm_miscellaneous_information.pdf

Supporting Information

1. **Figures and Tables** **S2**
2. **Synthesis of New Compounds** **S11**
3. **NMR Experiments** **S30**
4. **Computational Details, Cartesian Coordinates and Absolute Energies for All Calculated Compounds** **S39**
5. **^{1}H NMR and ^{13}C NMR Spectra of New Compounds** **S68**
6. **Supporting Information References** **S91**

1. Figures and Tables

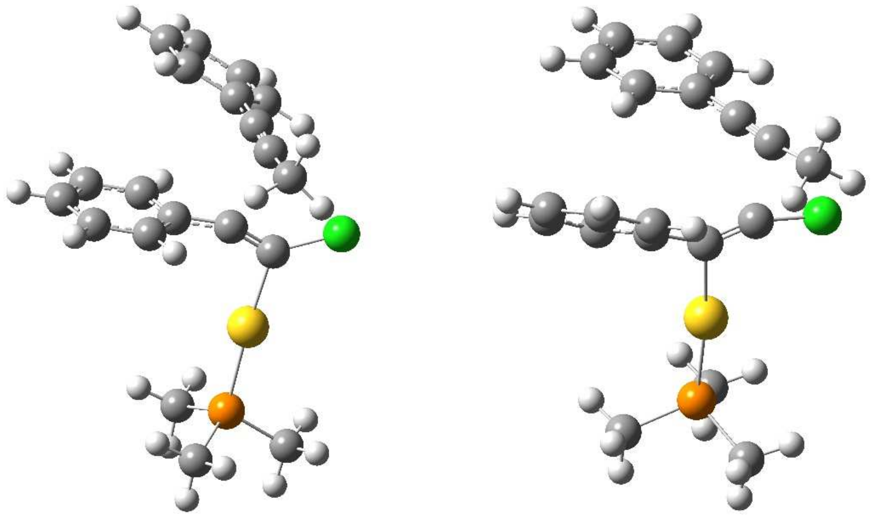

Figure S1. Molecular structures of the transition states **15** (left) and **21** (right) calculated using B3LYP-D3BJ/6-31G(d),def2-TZVP+ECP. $[Au]^+ = Me_3PAu^+$.

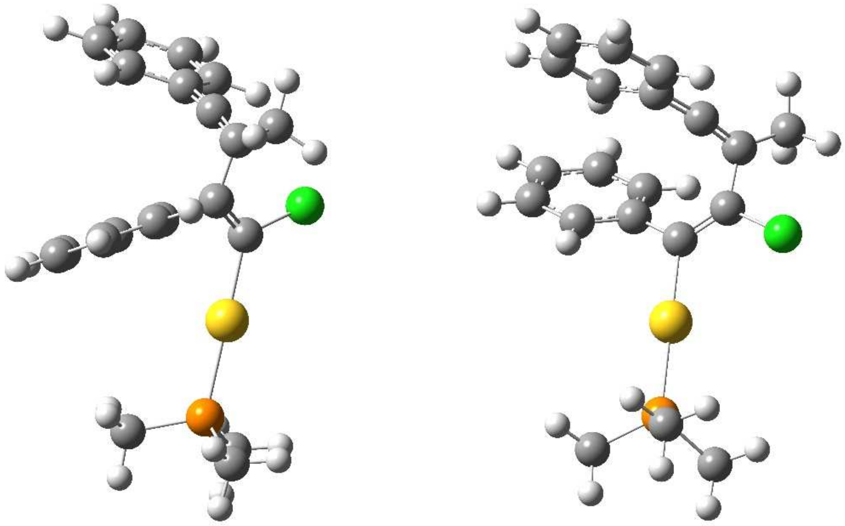

Figure S2. Molecular structures of the intermediates **16** (left) and **22** (right) calculated using B3LYP-D3BJ/6-31G(d),def2-TZVP+ECP. $[Au]^+ = Me_3PAu^+$.

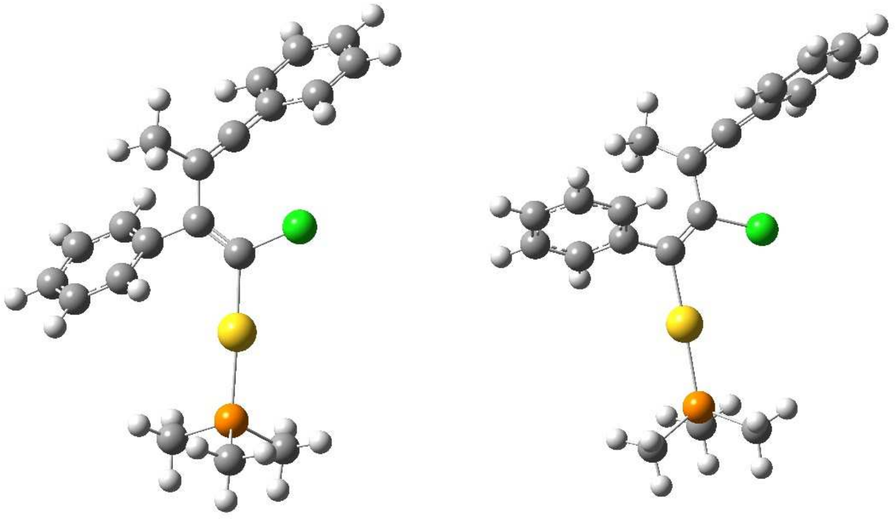

Figure S3. Molecular structures of the transition states **17** (left) and **23** (right) calculated using B3LYP-D3BJ/6-31G(d),def2-TZVP+ECP. $[Au]^+ = Me_3PAu^+$.

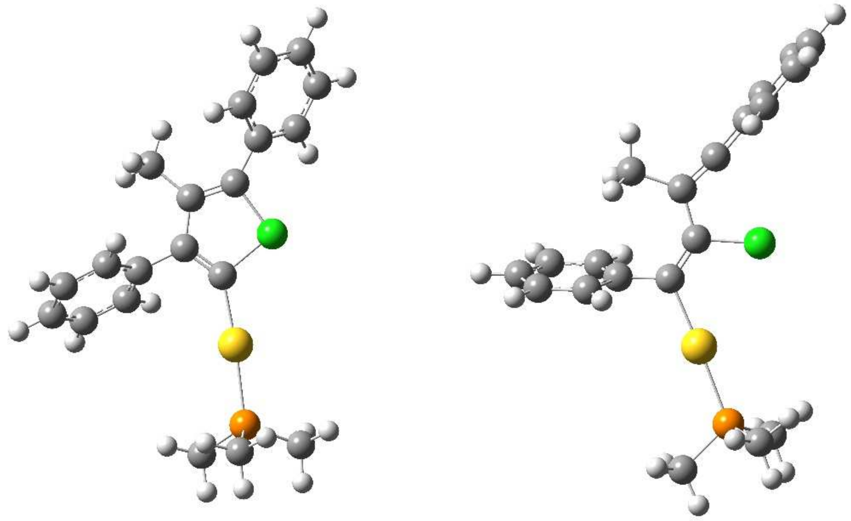

Figure S4. Molecular structures of the intermediates **18** (left) and **24** (right) calculated using B3LYP-D3BJ/6-31G(d),def2-TZVP+ECP. $[Au]^+ = Me_3PAu^+$.

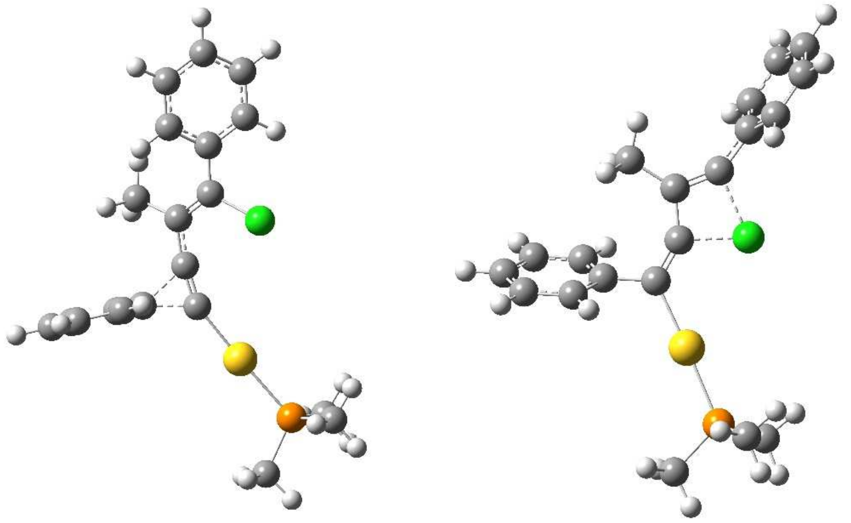

Figure S5. Molecular structures of the transition states **19** (left) and **25** (right) calculated using B3LYP-D3BJ/6-31G(d),def2-TZVP+ECP. $[Au]^+ = Me_3PAu^+$.

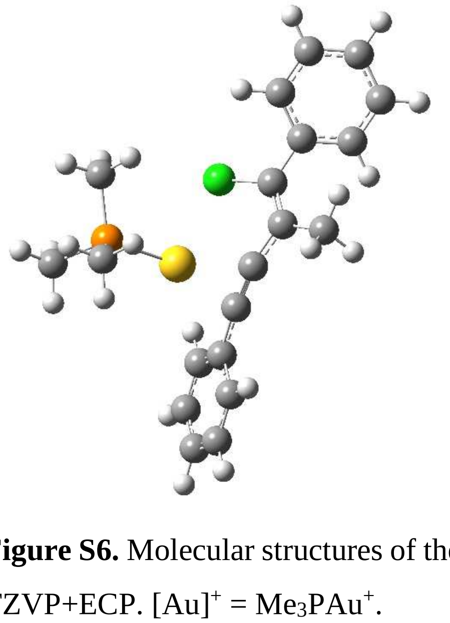

Figure S6. Molecular structures of the complex **20** calculated using B3LYP-D3BJ/6-31G(d),def2-TZVP+ECP. $[Au]^+ = Me_3PAu^+$.

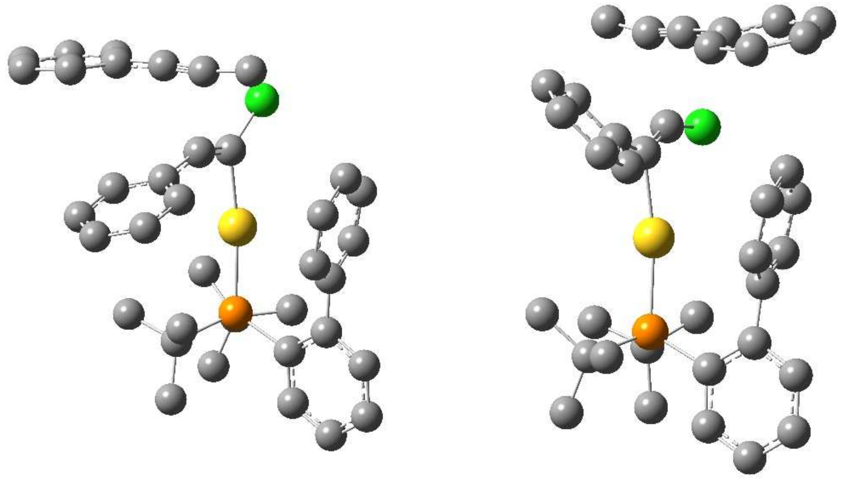

Figure S7. Molecular structures of the transition states **15** (left) and **21** (right) calculated using B3LYP-D3BJ/6-31G(d),def2-TZVP+ECP. $[Au]^+$ = $JohnPhosAu^+$. All hydrogen atoms are omitted for clarity.

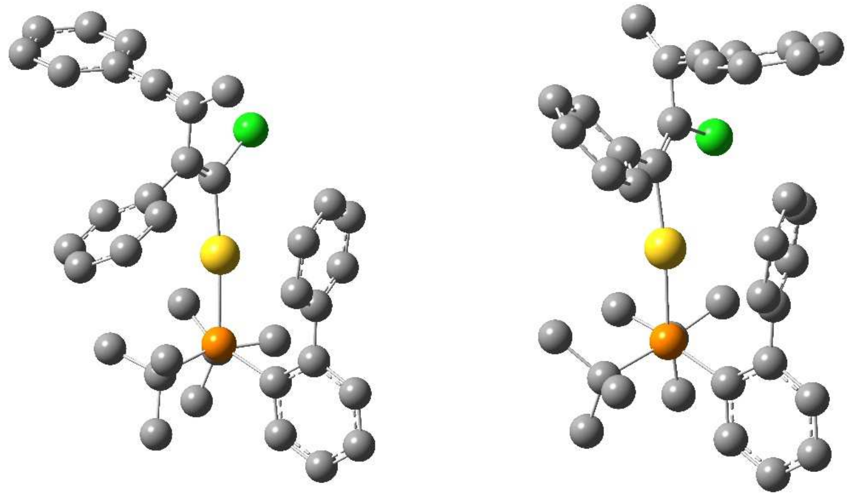

Figure S8. Molecular structures of the intermediates **16** (left) and **22** (right) calculated using B3LYP-D3BJ/6-31G(d),def2-TZVP+ECP. $[Au]^+$ = $JohnPhosAu^+$. All hydrogen atoms are omitted for clarity.

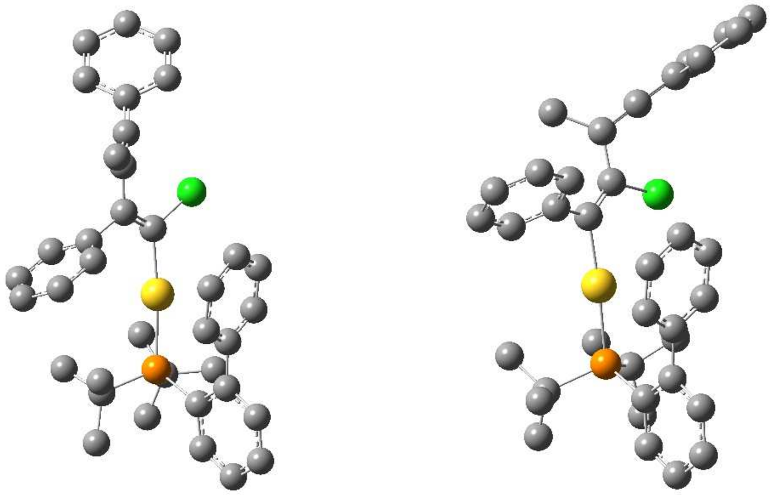

Figure S9. Molecular structures of the transition states **17** (left) and **23** (right) calculated using B3LYP-D3BJ/6-31G(d),def2-TZVP+ECP. $[Au]^+$ = $JohnPhosAu^+$. All hydrogen atoms are omitted for clarity.

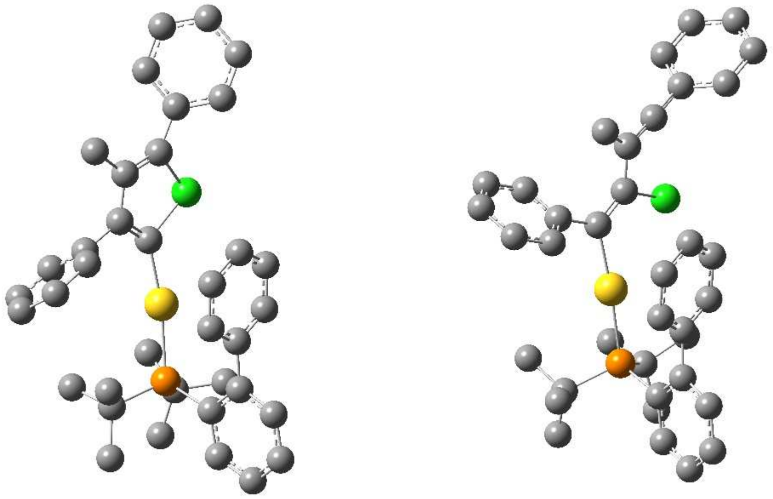

Figure S10. Molecular structures of the intermediates **18** (left) and **24** (right) calculated using B3LYP-D3BJ/6-31G(d),def2-TZVP+ECP. $[Au]^+$ = $JohnPhosAu^+$. All hydrogen atoms are omitted for clarity.

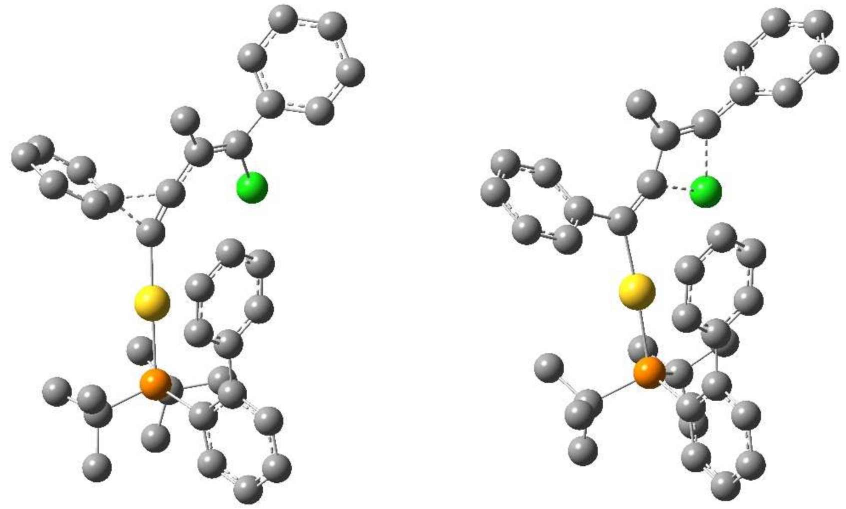

Figure S11. Molecular structures of the transition states **19** (left) and **25** (right) calculated using B3LYP-D3BJ/6-31G(d),def2-TZVP+ECP. $[Au]^+$ = $JohnPhosAu^+$. All hydrogen atoms are omitted for clarity.

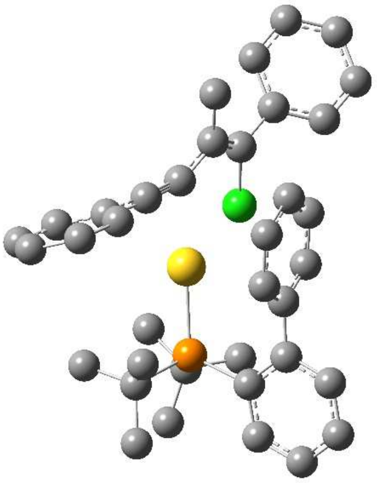

Figure S12. Molecular structures of the complex **20** calculated using B3LYP-D3BJ/6-31G(d),def2-TZVP+ECP. $[Au]^+$ = $JohnPhosAu^+$. All hydrogen atoms are omitted for clarity.

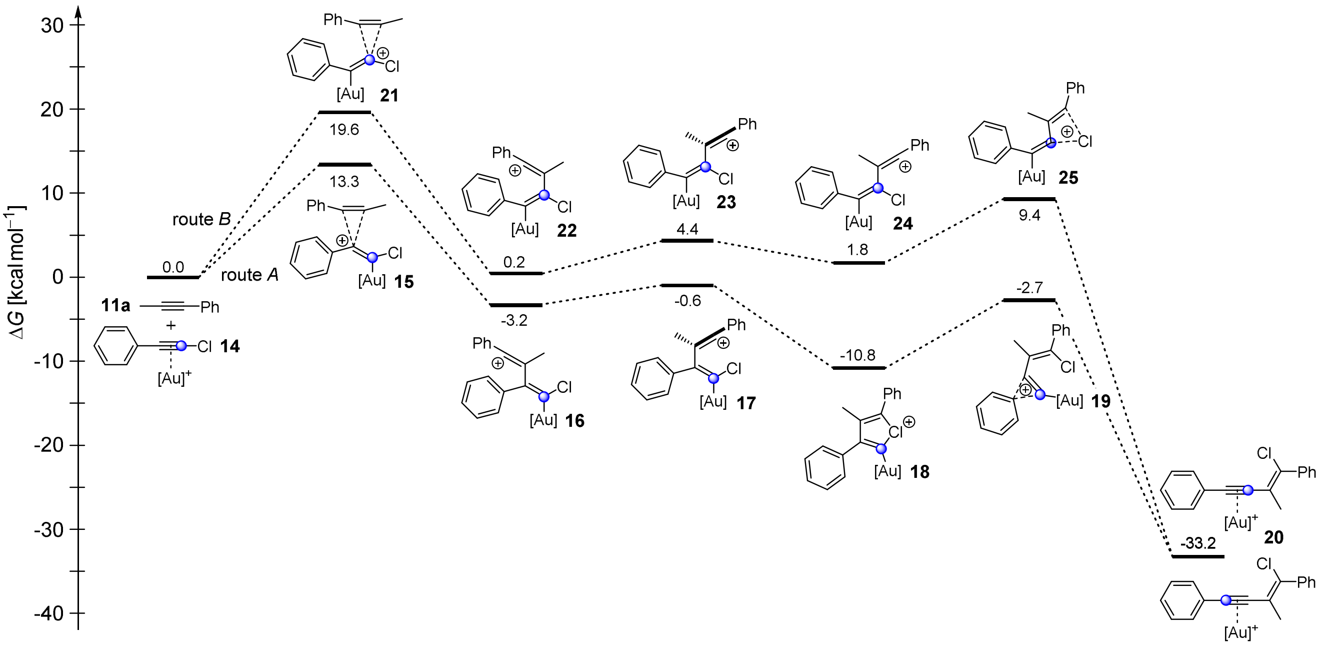

Figure S13. Free-energy (Δ*G*) profile for the gold(I)-catalyzed 1,2-haloalkynylation of alkyne **11a** via an attack at the carbon atom C2 (route *A*) and C1 (route *B*) of alkyne complex **14**, respectively, calculated by means of B3LYP-D3BJ(SMD). $[Au]^+$ = Me_3PAu^+.

Table S1. Free enthalpy (Δ*G* in kcal/mol) of **15-25** relative to the starting materials (**11a** and **14**) as calculated by means different methods. $[Au]^+ = Me_3PAu^+$.

	Δ*G*[a]	Δ*G*[b]	Δ*G*[c]	Δ*G*[d]	Δ*G*[e]	Δ*G*[f]	Δ*G*[g]	Δ*G*[h]
**15**	1.3	4.1	4.4	13.0	13.3	14.7	18.2	9.9
**16**	-17.8	-12.2	-12.1	-3.5	-3.2	-6.2	3.4	-5.8
**17**	-14.1	-8.3	-8.1	-0.8	-0.6	-2.8	5.7	-2.3
**18**	-26.7	-20.8	-20.7	-11.0	-10.8	-18.7	-9.4	-11.4
**19**	-17.5	-11.1	-11.1	-2.7	-2.7	-9.2	-4.0	-4.1
**20**	-43.4	-38.9	-38.8	-33.4	-33.2	-37.5	-34.1	-32.2
**21**	9.4	11.7	11.9	19.3	19.6	20.4	21.4	16.1
**22**	-15.0	-9.0	-8.8	0.0	0.2	-2.5	6.9	-2.4
**23**	-9.0	-3.5	-3.3	4.2	4.4	2.3	11.0	1.8
**24**	-11.9	-6.3	-6.2	1.5	1.8	-0.6	8.9	-0.5
**25**	-6.0	-0.8	-0.6	9.2	9.4	4.7	15.6	6.8

[a] B3LYP-D3BJ/B1. [b] B3LYP-D3BJ/B2//B3LYP-D3BJ/B1.

[c] B3LYP-D3BJ/B3//B3LYP-D3BJ/B1.

[d] B3LYP-D3BJ(dichloroethane as solvent)/B2//B3LYP-D3BJ/B1.

[e] B3LYP-D3BJ(dichloroethane as solvent)/B3//B3LYP-D3BJ/B1.

[f] PBE0-D3BJ(dichloroethane as solvent)/B3//B3LYP-D3BJ/B1.

[g] M06-2X-D3(dichloroethane as solvent)/B3//B3LYP-D3BJ/B1.

[h] B97D3(dichloroethane as solvent)/B3//B3LYP-D3BJ/B1.

Table S2. Free enthalpy (Δ*G* in kcal/mol) of **15-25** relative to the starting materials (**11a** and **14**) as calculated by means different methods. $[Au]^+$ = $JohnPhosAu^+$.

	Δ*G*[a]	Δ*G*[b]	Δ*G*[c]
**15**	3.9	7.2	13.8
**16**	-13.4	-7.6	-1.4
**17**	-8.7	-3.1	1.6
**18**	-23.8	-18.0	-10.8
**19**	-14.1	-8.0	-1.7
**20**	-42.6	-37.4	-31.4
**21**	10.6	14.0	21.3
**22**	-10.6	-4.5	1.9
**23**	-4.0	1.3	6.1
**24**	-5.5	-0.3	4.3
**25**	-1.9	2.9	9.7

[a] B3LYP-D3BJ/B1. [b] B3LYP-D3BJ/B3//B3LYP-D3BJ/B1.

[c] B3LYP-D3BJ(dichloroethane as solvent)/B3//B3LYP-D3BJ/B1.

2. Synthesis of New Compounds

General remarks: Chemicals were purchased from ABCR, Alfa Aesar, Acros Organics, Carbolution, TCI, or Sigma-Aldrich. All chemicals were reagent grade and used without further purification. Reactions were monitored by TLC analysis with silica gel 60 F254 thin-layer plates. Flash chromatography was carried out on silica 60 (40−63 µm, 230−400 mesh). ^{1}H and ^{13}C NMR spectra were measured with Bruker Avance NEO 400 and Avance HD 600 spectrometers. All chemical shifts (δ) are given in ppm. The spectra were referenced to the peak for the protium impurity in the deuterated solvents indicated in brackets in the analytical data ($CDCl_3$, ^{1}H: 7.26 ppm, ^{13}C: 77.16 ppm; 1,2-dichloroethane-d_4, ^{1}H: 3.76 ppm; C_6D_6, ^{1}H: 7.16 ppm, ^{13}C: 128.06 ppm). Signal multiplicities were determined as s (singlet), d (doublet), t (triplet), q (quartet), m (multiplet), dd (doublet of doublets), and td (triplet of doublets). Coupling constants (*J*) are reported in Hertz (Hz). ^{13}C NMR spectra were measured with ^{1}H decoupling and the ^{13}C assignment was achieved via distortionless enhancement by polarization transfer 135, heteronuclear single quantum coherence, HMBC, and correlation spectroscopy spectra. High-resolution mass spectra (HRMS) were recorded with a Bruker BioTOF III spectrometer with electrospray ionization (ESI), atmospheric pressure chemical ionization (APCI) and with a JEOL AccuTOF GCx spectrometer with electron ionization (EI) as the ionization source. UV/Vis absorption spectra were obtained with a Jasco V-550 spectrophotometer. IR absorption spectra were recorded with a Shimadzu IR Tracer-100 Fourier-transform infrared spectrophotometer. Characteristic IR absorption frequencies ($\tilde{\nu}$) are reported as absorption maxima in cm^{-1} and their intensities were determined as very strong (vs), strong (s), medium (m), weak (w), and very weak (vw). Melting points were measured with a Büchi melting point apparatus Model B-540 with an open capillary and are uncorrected.

Preparation of Known Arylalkynes 11

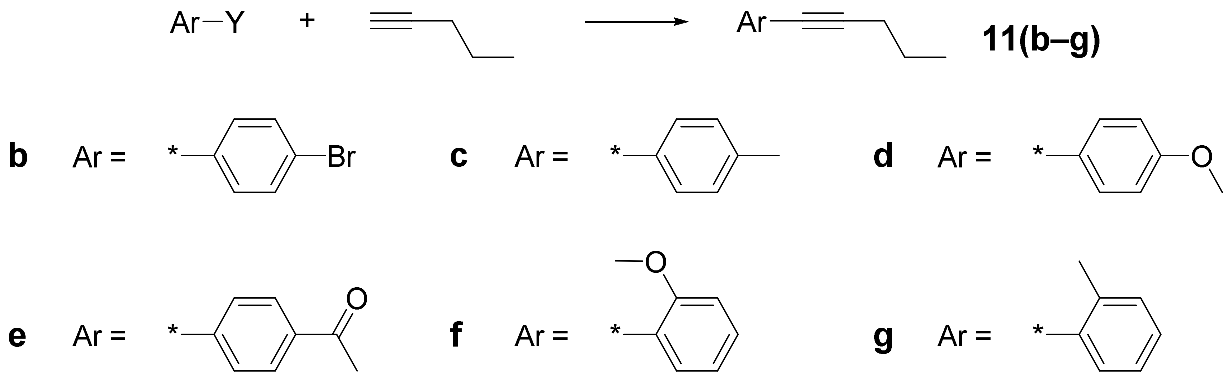

1-Phenyl-1-propyne (**11a**), 1-phenyl-1-butyne and 1-phenyl-1-pentyne were purchased from Sigma-Aldrich. 1-Bromo-4-(pent-1-yn-1-yl)benzene (**11b**)[1], 1-methyl-4-(pent-1-yn-1-yl)benzene (**11c**)[1], 1-methoxy-4-(pent-1-yn-1-yl)benzene (**11d**)[1], 1-(4-(pent-1-yn-1-yl)phenyl)ethan-1-one (**11e**)[1], 1-methoxy-2-(pent-1-yn-1-yl)benzene (**11f**)[2] and 1-methyl-2-(pent-1-yn-1-yl)benzene (**11g**)[3] were prepared from the corresponding haloarenes Ar-Y (Y = I or Br) by Sonogashira coupling with 1-pentyne according to the literature procedure.[1]

Preparation of Known Haloarylacetylenes 1 and 4

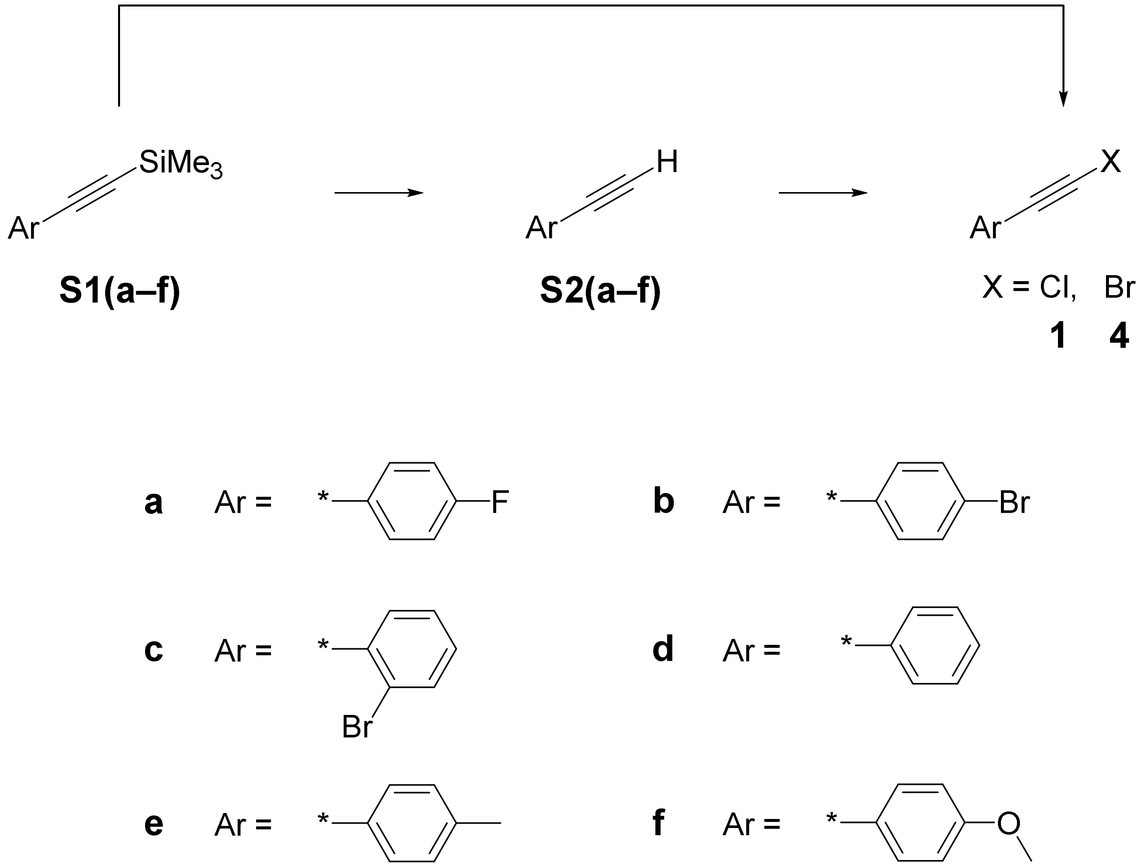

1-(Chloroethynyl)-4-fluorobenzene (**1a**)[4], (chloroethynyl)benzene (**1d**)[4] 1-(chloroethynyl)-4-methylbenzene (**1e**)[4] and 1-(chloroethynyl)-4-methoxybenzene (**1f**)[4] were chlorinated according to the literature procedure[4] starting from the corresponding commercially available terminal acetylene **S2a**, **S2d**, **S2e** and **S2f**. 1-Bromo-4-(chloroethynyl)benzene (**1b**)[4] and 1-bromo-2-(chloroethynyl)benzene (**1c**)[5] were obtained via direct chlorination[6] of the corresponding

trimethylsilylacetylene **S1b** and **S1c**, respectively. 1-(Bromoethynyl)-4-methylbenzene (**4e**)[7] was obtained via bromination[8] of the terminal acetylene **S2e**.

Preparation of ^{13}C-Labeled (Chloroethynyl)benzene (^{13}C-1d)

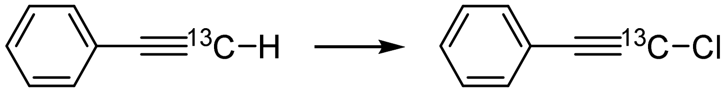

^{13}C-**S2d** ^{13}C-**1d**

Synthesis of chlorophenylacetylene (^{13}C-1d). In analogy to the literature procedure[9], a Schlenk flask was charged with ^{13}C-labeled phenylacetylene (^{13}C-**S2d**)[i] (300 mg, 2.94 mmol, 1.0 eq) and anhydrous THF (6 mL) under argon atmosphere. The flask was cooled to −78 °C and then treated with *n*-butyllithium (2.5 M in *n*-hexane, 3.53 mmol, 1.41 mL, 1.2 eq) over 5 min. After stirring the solution for 30 min at −78 °C, *N*-chlorosuccinimide (431 mg, 3.23 mmol, 1.1 eq) was added in one portion. The reaction mixture was allowed to warm to room temperature. The reaction was quenched with a solution of saturated NH_4Cl (15 mL). After diluting with Et_2O (30 mL), the phases were separated and the aqueous phase was extracted with Et_2O (3 x 30 mL). The combined organic phases were washed with brine and dried over magnesium sulfate. After removing the solvent in vacuo, the residue was adsorbed onto Celite® and purified by flash chromatography (SiO_2, *n*-hexane) to yield ^{13}C-**1d** (185 mg, 1.35 mmol, 46%) as a colorless oil. R_f (*n*-hexane) = 0.62. ^{1}H NMR (400 MHz, $CDCl_3$): δ = 7.47–7.42 (m, 2H, CH_{ar}), 7.37–7.28 ppm (m, 3H, CH_{ar}). ^{13}C NMR (101 MHz, $CDCl_3$): δ = 132.1 (d, $^3J_{C\text{-}C}$ = 3.1 Hz, CH_{ar}), 128.7 (CH_{ar}), 128.5 (CH_{ar}), 122.3 (d, $^2J_{C\text{-}C}$ = 15 Hz, C_{ar}), 68.5 (d, $^1J_{C\text{-}C}$ = 94.4 Hz, *C*≡C-Cl), 68.2 ppm (C≡*C*-Cl).

---

[i] This compound was purchased from Sigma-Aldrich. For the ^{1}H NMR spectrum of the ^{13}C-labeled phenylacetylene (^{13}C-**S2d**) see Figure S20.

General Procedure: Gold(I)-Catalyzed Haloalkynylation of Arylalkylalkynes 11

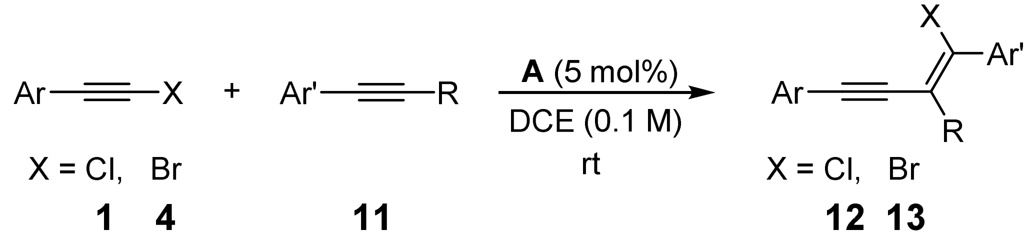

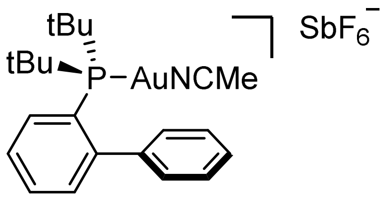

A

In a 5 mL screw-capped vial, 1.0 eq. haloarylacetylene **1** and **4**, respectively (400 µmol) and 2.0 eq. arylalkylalkyne **11** (800 µmol) were dissolved in dry 1,2-dichloroethane (DCE) (4 mL). Then 5 mol% [JohnPhosAu(NCMe)]SbF_6 (**A**) (20 µmol, 15.4 mg) was added. The mixture was stirred at room temperature until full conversion of the haloarylacetylene. Upon completion, the solvent was removed under reduced pressure. The crude product was adsorbed onto Celite® and purified by flash chromatography to yield the desired enyne **12** and **13**, respectively.

Synthesis and Characterization of Enynes 12 and 13

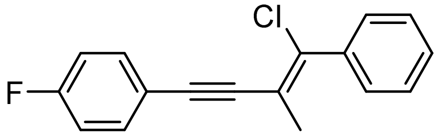

12a

Synthesis of enyne 12a. According to the general procedure, chloroarylacetylene **1a** (61.8 mg, 400 µmol, 1.0 eq) and 1-phenyl-1-propyne (**11a**) (92.9 mg, 800 µmol, 2.0 eq) were dissolved in dry DCE (4 mL). Then [JohnPhosAu(NCMe)]SbF_6 (**A**) (5 mol%, 20 µmol, 15.4 mg) was added. The mixture was stirred at room temperature for 20 hours. After removing the solvent in vacuo, the residue was adsorbed onto Celite® and purified by flash chromatography (SiO_2, *n*-hexane/Et_2O 200:1) to yield **12a** (73.4 mg, 271 µmol, 68%) as a pale yellow solid. M.p.: 94–96 °C. R_f (*n*-hexane/EtOAc 99:1) = 0.35. ^{1}H NMR (600 MHz, $CDCl_3$): δ = 7.53–7.48 (m, 2H, CH_{ar}), 7.43–7.37

(m, 4H, CH_{ar}), 7.37–7.33 (m, 1H, CH_{ar}), 7.07–7.02 (m, 2H, CH_{ar}), 2.01 ppm (s, 3H, CH_3). ^{13}C NMR (151 MHz, $CDCl_3$): δ = 162.8 (d, $^1J_{C\text{-}F}$ = 249.8 Hz, $C_{ar}F$), 137.7 (C_{ar}), 135.6 (C=*C*Cl), 133.7 (d, $^3J_{C\text{-}F}$ = 8.6 Hz, $C_{ar}H$), 129.1 ($C_{ar}H$), 129.0 ($C_{ar}H$), 128.4 ($C_{ar}H$), 119.4 (d, $^4J_{C\text{-}F}$ = 3.3 Hz, C_{ar}), 117.5 (*C*=CCl), 115.8 (d, $^2J_{C\text{-}F}$ = 22.0 Hz, $C_{ar}H$), 94.6 (C≡C), 89.0 (C≡C), 20.9 ppm (CH_3). ^{19}F NMR (565 Hz): −110.56 ppm (m, CF_{ar}). IR (ATR): $\tilde{\nu}$ = 3100 (vw), 3065 (vw), 3003 (vw), 2961 (vw), 2924 (vw), 2114 (vw), 2087 (vw), 1586 (vw) 1503 (w), 1217 (w), 835 (w), 762 (w), 694 cm^{-1} (w). UV/Vis (CH_3CN): λ_{max} (log ε) = 295 nm (4.29). HRMS (EI): $[C_{17}H_{12}{}^{35}ClF]^+$: calculated: 270.0606; observed: 270.0611.

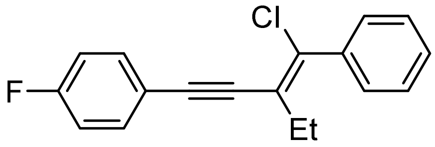

12b

Synthesis of enyne 12b. According to the general procedure, chloroarylacetylene **1a** (61.8 mg, 400 µmol, 1.0 eq) and 1-phenyl-1-butyne (104.2 mg, 800 µmol, 2.0 eq) were dissolved in dry DCE (4 mL). Then [JohnPhosAu(NCMe)]SbF_6 (**A**) (5 mol%, 20 µmol, 15.4 mg) was added. The mixture was stirred at room temperature for 18 hours. After removing the solvent in vacuo, the residue was adsorbed onto Celite® and purified by flash chromatography (SiO_2, *n*-hexane/Et_2O 200:1) to yield **12b** (78.9 mg, 277 µmol, 69%) as a white solid. M.p.: 51–53 °C. R_f (*n*-hexane/EtOAc 99:1) = 0.35. ^{1}H NMR (600 MHz, $CDCl_3$): δ = 7.54–7.49 (m, 2H, CH_{ar}), 7.41–7.34 (m, 5H, CH_{ar}), 7.07–7.02 (m, 2H, CH_{ar}), 2.30 (q, $^3J_{H\text{-}H}$ = 7.5 Hz, 2H, CH_2), 1.18 ppm (t, $^3J_{H\text{-}H}$ = 7.5 Hz, 3H, CH_3). ^{13}C NMR (151 MHz, $CDCl_3$): δ = 162.8 (d, $^1J_{C\text{-}F}$ = 250.2 Hz, $C_{ar}F$), 137.9 (C_{ar}), 135.3 (C=*C*Cl), 133.7 (d, $^3J_{C\text{-}F}$ = 8.6 Hz, $C_{ar}H$), 129.0 ($C_{ar}H$), 128.9 ($C_{ar}H$), 128.5 ($C_{ar}H$), 124.3 (*C*=CCl), 119.5 (d, $^4J_{C\text{-}F}$ = 3.4 Hz, C_{ar}), 115.8 (d, $^2J_{C\text{-}F}$ = 22.1 Hz, $C_{ar}H$), 95.5 (C≡C), 87.7 (C≡C), 27.3 (CH_2), 13.7 ppm (CH_3). ^{19}F NMR (565 Hz): −110.61 ppm (m, CF_{ar}). IR (ATR): $\tilde{\nu}$ = 3100 (vw), 3053 (vw), 2965 (vw), 2955 (vw), 2924 (vw), 2870 (vw), 2197 (vw), 2112 (vw), 1593 (vw), 1501 (w), 1236 (w), 833 cm^{-1} (m). UV/Vis (CH_3CN): λ_{max} (log ε) = 295 nm (4.26). HRMS (EI): $[C_{18}H_{14}{}^{35}ClF]^+$: calculated: 284.0763; observed: 284.0758.

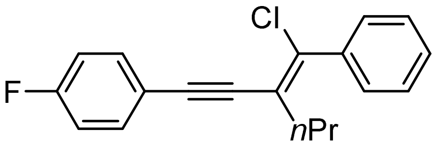

12c

Synthesis of enyne 12c. According to the general procedure, chloroarylacetylene **1a** (61.8 mg, 400 µmol, 1.0 eq) and 1-phenyl-1-pentyne (115.4 mg, 800 µmol, 2.0 eq) were dissolved in dry DCE (4 mL). Then [JohnPhosAu(NCMe)]SbF_6 (**A**) (5 mol%, 20 µmol, 15.4 mg) was added. The mixture was stirred at room temperature for 20 hours. After removing the solvent in vacuo, the residue was adsorbed onto Celite® and purified by flash chromatography (SiO_2, *n*-hexane/DCM 99:1) to yield **12c** (68.3 mg, 229 µmol, 57%) as a colorless oil. R_f (*n*-hexane/EtOAc 99:1) = 0.43. ^{1}H NMR (600 MHz, $CDCl_3$): δ = 7.53–7.49 (m, 2H, CH_{ar}), 7.41–7.34 (m, 5H, CH_{ar}), 7.07–7.02 (m, 2H, CH_{ar}), 2.27–2.22 (m, 2H, CH_2), 1.70–1.62 (m, 2H, CH_2), 0.89 ppm (t, $^3J_{H\text{-}H}$ = 7.5 Hz, 3H, CH_3). ^{13}C NMR (151 MHz, $CDCl_3$): δ = 162.8 (d, $^1J_{C\text{-}F}$ = 249.8 Hz, C_{ar}F), 137.9 (C_{ar}), 135.7 (C=*C*Cl), 133.7 (d, $^3J_{C\text{-}F}$ = 7.9 Hz, C_{ar}H), 129.0 (C_{ar}H), 128.9 (C_{ar}H), 128.5 (C_{ar}H), 123.0 (*C*=CCl), 119.5 (d, $^4J_{C\text{-}F}$ = 3.3 Hz, C_{ar}), 115.8 (d, $^2J_{C\text{-}F}$ = 22.3 Hz, C_{ar}H) , 95.4 (C≡C), 87.8 (C≡C), 35.6 (CH_2), 22.3 (CH_2), 13.7 ppm (CH_3). ^{19}F NMR (565 Hz): −110.63 ppm (m, CF_{ar}). IR (ATR): $\tilde{\nu}$ = 3079 (vw), 3057 (vw), 2961 (vw), 2928 (vw), 2870 (vw), 2114 (vw), 2089 (vw), 1599 (vw), 1505 (w), 1229 (w), 1153 (w), 833 (w), 758 (w), 696 cm^{-1} (w). UV/Vis (CH_3CN): λ_{max} (log ε) = 295 nm (4.38). HRMS (EI): $[C_{19}H_{16}{}^{35}ClF]^+$: calculated: 298.0919; observed: 298.0916.

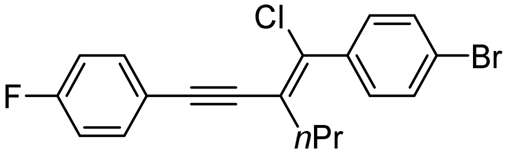

12d

Synthesis of enyne 12d. According to the general procedure, chloroarylacetylene **1a** (61.8 mg, 400 µmol, 1.0 eq) and 1-bromo-4-(pent-1-yn-1-yl)benzene (**11b**) (186.5 mg, 800 µmol, 2.0 eq) were dissolved in dry DCE (4 mL). Then [JohnPhosAu(NCMe)]SbF_6 (**A**) (5 mol%, 20 µmol, 15.4 mg) was added. The mixture was stirred at room temperature for 68 hours. After removing the solvent in vacuo, the residue was adsorbed onto Celite® and purified by flash chromatography (SiO_2, *n*-hexane) to yield **12d** (83.4 mg, 221 µmol, 55%) as a pale yellow solid. M.p.: 72–74 °C. R_f (*n*-hexane) = 0.24. ^{1}H NMR (600 MHz, $CDCl_3$): δ = 7.54–7.48 (m, 4H, CH_{ar}), 7.25–7.22 (m, 2H, CH_{ar}), 7.07–7.02 (m, 2H, CH_{ar}), 2.25–2.20 (m, 2H, CH_2), 1.69–1.62 (m, 2H, CH_2), 0.89 ppm (t,

$^{3}J_{H\text{-}H}$ = 7.4 Hz, 3H, CH_3). ^{13}C NMR (151 MHz, $CDCl_3$): δ = 162.9 (d, $^{1}J_{C\text{-}F}$ = 250.6 Hz, C_{ar}F), 136.8 (C_{ar}), 134.3 (C=*C*Cl), 133.8 (d, $^{3}J_{C\text{-}F}$ = 8.7 Hz, C_{ar}H), 131.7 (C_{ar}H), 130.7 (C_{ar}H), 123.7 (*C*=CCl), 123.1 (C_{ar}), 119.3 (d, $^{4}J_{C\text{-}F}$ = 3.9 Hz, C_{ar}), 115.8 (d, $^{2}J_{C\text{-}F}$ = 22.1 Hz, C_{ar}H), 95.9 (C≡C), 87.6 (C≡C), 35.7 (CH_2), 22.2 (CH_2), 13.7 ppm (CH_3). ^{19}F NMR (565 Hz): −110.37 ppm (m, CF_{ar}). IR (ATR): $\tilde{\nu}$ = 3100 (vw), 3061 (vw), 2957 (vw), 2930 (vw), 2872 (vw), 2114 (vw), 1578 (vw), 1505 (w), 1221 (w), 826 (w), 810 cm^{-1} (w). UV/Vis (CH_3CN): λ_{max} (log ε) = 297 nm (4.45). HRMS (EI): $[C_{19}H_{15}{}^{79}Br^{35}ClF]^+$: calculated: 376.0024; observed: 376.0031.

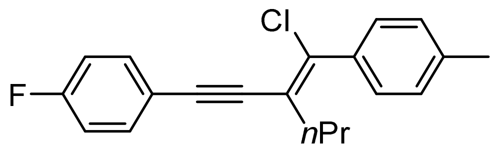

12e

Synthesis of enyne 12e. According to the general procedure, chloroarylacetylene **1a** (61.8 mg, 400 µmol, 1.0 eq) and 1-methyl-4-(pent-1-yn-1-yl)benzene (**11c**) (126.6 mg, 800 µmol, 2.0 eq) were dissolved in dry DCE (4 mL). Then [JohnPhosAu(NCMe)]SbF_6 (**A**) (5 mol%, 20 µmol, 15.4 mg) was added. The mixture was stirred at room temperature for 18 hours. After removing the solvent in vacuo, the residue was adsorbed onto Celite® and purified by flash chromatography (SiO_2, *n*-hexane/Et_2O 200:1) to yield **12e** (94.5 mg, 302 µmol, 76%) as a white solid. M.p.: 56–58 °C. R_f (*n*-hexane/Et_2O 200:1) = 0.31. ^{1}H NMR (600 MHz, $CDCl_3$): δ = 7.54–7.48 (m, 2H, CH_{ar}), 7.28–7.25 (m, 2H, CH_{ar}), 7.22–7.18 (m, 2H, CH_{ar}), 7.07–7.02 (m, 2H, CH_{ar}), 2.39 (s, 3H, $C_{ar}CH_3$), 2.28–2.23 (m, 2H, CH_2), 1.70–1.62 (m, 2H, CH_2), 0.89 ppm (t, $^{3}J_{H\text{-}H}$ = 7.6 Hz, 3H, CH_3). ^{13}C NMR (151 MHz, $CDCl_3$): δ = 162.7 (d, $^{1}J_{C\text{-}F}$ = 249.7 Hz, C_{ar}F), 139.0 (*C*$_{ar}CH_3$), 136.0 (C=*C*Cl), 135.1 (C_{ar}), 133.7 (d, $^{3}J_{C\text{-}F}$ = 8.6 Hz, C_{ar}H), 129.1 (C_{ar}H), 128.9 (C_{ar}H), 122.6 (*C*=CCl), 119.6 (d, $^{4}J_{C\text{-}F}$ = 3.7 Hz, C_{ar}), 115.8 (d, $^{2}J_{C\text{-}F}$ = 22.0 Hz, C_{ar}H), 95.2 (C≡C), 88.0 (C≡C), 35.7 (CH_2), 22.3 (CH_2), 21.5 (C_{ar}*C*H_3), 13.7 ppm (CH_3). ^{19}F NMR (565 Hz): −110.74 ppm (m, CF_{ar}). IR (ATR): $\tilde{\nu}$ = 3102 (vw), 3065 (vw), 2963 (vw), 2924 (vw), 2870 (vw), 2100 (vw), 1591 (vw), 1503 (m), 1219 (w), 839 cm^{-1} (m). UV/Vis (CH_3CN): λ_{max} (log ε) = 296 nm (4.43). HRMS (EI): $[C_{20}H_{18}{}^{35}ClF]^+$: calculated: 312.1076; observed: 312.1075.

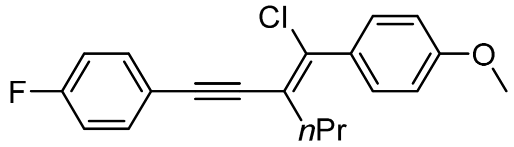

12f

Synthesis of enyne 12f. According to the general procedure, chloroarylacetylene **1a** (61.8 mg, 400 µmol, 1.0 eq) and 1-methoxy-4-(pent-1-yn-1-yl)benzene (**11d**) (139.4 mg, 800 µmol, 2.0 eq) were dissolved in dry DCE (4 mL). Then [JohnPhosAu(NCMe)]SbF_6 (**A**) (5 mol%, 20 µmol, 15.4 mg) was added. The mixture was stirred at room temperature for 20 hours. After removing the solvent in vacuo, the residue was adsorbed onto Celite® and purified by flash chromatography (SiO_2, *n*-hexane/Et_2O 98:2) to yield **12f** (118.5 mg, 360 µmol, 90%) as a pale yellow solid. M.p.: 70–72 °C. R_f (*n*-hexane/Et_2O 98:2) = 0.32. ^{1}H NMR (600 MHz, $CDCl_3$): δ = 7.53–7.48 (m, 2H, CH_{ar}), 7.33–7.29 (m, 2H, CH_{ar}), 7.06–7.01 (m, 2H, CH_{ar}), 6.93–6.89 (m, 2H, CH_{ar}), 3.84 (s, 3H, OCH_3), 2.28–2.24 (m, 2H, CH_2), 1.69–1.62 (m, 2H, CH_2). 0.89 ppm (t, $^3J_{H\text{-}H}$ = 7.5 Hz, 3H, CH_3). ^{13}C NMR (151 MHz, $CDCl_3$): δ = 162.7 (d, $^1J_{C\text{-}F}$ = 249.8 Hz, C_{ar}F), 160.0 ($C_{ar}OCH_3$), 135.8 (C=*C*Cl), 133.7 (d, $^3J_{C\text{-}F}$ = 8.6 Hz, C_{ar}H), 130.4 (C_{ar}H), 130.3 (C_{ar}), 122.3 (*C*=CCl), 119.6 (d, $^4J_{C\text{-}F}$ = 3.3 Hz, C_{ar}), 115.8 (d, $^2J_{C\text{-}F}$ = 22.1 Hz, C_{ar}H), 113.8 (C_{ar}H), 95.1 (C≡C), 88.1 (C≡C), 55.5 (OCH_3), 35.7 (CH_2), 22.3 (CH_2), 13.8 ppm (CH_3). ^{19}F NMR (565 Hz): −110.78 ppm (m, CF_{ar}). IR (ATR): $\tilde{\nu}$ = 3104 (vw), 3069 (vw), 2955 (vw), 2926 (vw), 2866 (vw), 2843 (vw), 2087 (vw), 1591 (w), 1499 (w), 1298 (w), 1236 (w), 1030 (w), 833 cm^{-1} (m). UV/Vis (CH_3CN): λ_{max} (log ε) = 297 nm (4.48). HRMS (ESI): $[C_{20}H_{18}{}^{35}ClFO+H]^+$: calculated: 329.1103; observed: 329.1103.

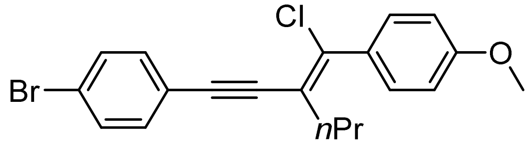

12g

Synthesis of enyne 12g. According to the general procedure, chloroarylacetylene **1b** (86.2 mg, 400 µmol, 1.0 eq) and 1-methoxy-4-(pent-1-yn-1-yl)benzene (**11d**) (139.4 mg, 800 µmol, 2.0 eq) were dissolved in dry DCE (4 mL). Then [JohnPhosAu(NCMe)]SbF_6 (**A**) (5 mol%, 20 µmol, 15.4 mg) was added. The mixture was stirred at room temperature for 25 hours. After removing the solvent in vacuo, the residue was adsorbed onto Celite® and purified by flash chromatography (SiO_2, *n*-hexane/Et_2O 97:3) to yield **12g** (138.4 mg, 355 µmol, 89%) as a pale yellow solid. M.p.: 56–58 °C. R_f (*n*-hexane/Et_2O 97:3) = 0.31. ^{1}H NMR (600 MHz, $CDCl_3$): δ = 7.49–7.45 (m, 2H, CH_{ar}), 7.40–

7.36 (m, 2H, CH_{ar}), 7.33–7.29 (m, 2H, CH_{ar}), 6.93–6.88 (m, 2H, CH_{ar}), 3.84 (s, 3H, OCH_3), 2.28–2.23 (m, 2H, CH_2), 1.69–1.61 (m, 2H, CH_2), 0.89 ppm (t, $^3J_{H-H}$ = 7.7 Hz, 3H, CH_3). ^{13}C NMR (151 MHz, $CDCl_3$): δ = 160.0 ($C_{ar}OCH_3$), 136.3 (C=*C*Cl), 133.2 (C_{ar}H), 131.7 (C_{ar}H), 130.4 (C_{ar}H), 130.2 (C_{ar}), 122.7 (C_{ar}Br), 122.4 (C_{ar}), 122.2 (*C*=CCl), 113.8 (C_{ar}H), 95.1 (C≡C), 89.5 (C≡C), 55.5 (OCH_3), 35.6 (CH_2), 22.3 (CH_2), 13.8 ppm (CH_3). IR (ATR): $\tilde{\nu}$ = 3053 (vw), 3013 (vw), 2963 (vw), 2930 (vw), 2870 (vw), 2835 (vw), 2116 (vw), 1603 (vw), 1506 (vw), 1252 (w), 826 cm^{-1} (m). UV/Vis (CH_3CN): λ_{max} (log ε) = 305 nm (4.50). HRMS (ESI): $[C_{20}H_{18}{}^{79}Br^{35}ClO+H]^+$: calculated: 389.0302; observed: 389.0300.

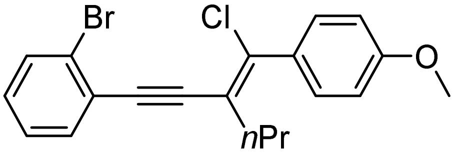

12h

Synthesis of enyne 12h. According to the general procedure, chloroarylacetylene **1c** (86.2 mg, 400 µmol, 1.0 eq) and 1-methoxy-4-(pent-1-yn-1-yl)benzene (**11d**) (139.4 mg, 800 µmol, 2.0 eq) were dissolved in dry DCE (4 mL). Then [JohnPhosAu(NCMe)]SbF_6 (**A**) (5 mol%, 20 µmol, 15.4 mg) was added. The mixture was stirred at room temperature for 7 days. After removing the solvent in vacuo, the residue was adsorbed onto Celite® and purified by flash chromatography (SiO_2, *n*-hexane/Et_2O 97:3) to yield **12h** (124.5 mg, 319 µmol, 80%) as a pale yellow solid. M.p.: 51–53 °C. R_f (*n*-hexane/Et_2O 97:3) = 0.25. ^{1}H NMR (600 MHz, $CDCl_3$): δ = 7.63–7.59 (dd, $^3J_{H-H}$ = 8.0 Hz, $^4J_{H-H}$ = 1.1 Hz, 1H, CH_{ar}), 7.58–7.54 (dd, $^3J_{H-H}$ = 7.7 Hz, $^4J_{H-H}$ = 1.6 Hz, 1H, CH_{ar}), 7.35–7.31 (m, 2H, CH_{ar}), 7.31–7.27 (td, $^3J_{H-H}$ = 7.5 Hz, $^4J_{H-H}$ = 1.3 Hz, 1H, CH_{ar}), 7.20–7.15 (td, $^3J_{H-H}$ = 7.8 Hz, $^4J_{H-H}$ = 1.5 Hz, 1H, CH_{ar}), 6.94–6.89 (m, 2H, CH_{ar}), 3.84 (s, 3H, CH_3), 2.34–2.27 (m, 2H, CH_2), 1.78–1.70 (m, 2H, CH_2), 0.90 ppm (t, $^3J_{H-H}$ = 7.7 Hz, 3H, CH_3). ^{13}C NMR (151 MHz, $CDCl_3$): δ = 160.0 ($C_{ar}OCH_3$), 136.6 (C=*C*Cl), 133.7 (C_{ar}H), 132.6 (C_{ar}H), 130.4 (C_{ar}H), 130.3 (C_{ar}), 129.6 (C_{ar}H), 127.1 (C_{ar}H), 125.7 (C_{ar}), 125.6 (C_{ar}Br), 122.3 (*C*=CCl), 113.8 (C_{ar}H), 94.7 (C≡C), 92.8 (C≡C), 55.5 (OCH_3), 35.7 (CH_2), 22.2 (CH_2), 13.8 ppm (CH_3). IR (ATR): $\tilde{\nu}$ = 3094 (vw), 3065 (vw), 3009 (vw), 2957 (vw), 2930 (vw), 2868 (vw), 2835 (vw), 2116 (vw), 2087 (vw), 1599 (vw), 1506 (w), 1248 (w), 1027 (m), 827 (m), 758 cm^{-1} (m). UV/Vis (CH_3CN): λ_{max} (log ε) = 306 nm (4.42). HRMS (ESI): $[C_{20}H_{18}{}^{79}Br^{35}ClO+H]^+$: calculated: 389.0302; observed: 389.0303.

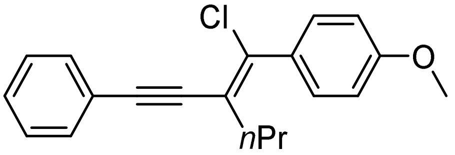

12i

Synthesis of enyne 12i. According to the general procedure, chloroarylacetylene **1d** (54.6 mg, 400 µmol, 1.0 eq) and 1-methoxy-4-(pent-1-yn-1-yl)benzene (**11d**) (139.4 mg, 800 µmol, 2.0 eq) were dissolved in dry DCE (4 mL). Then [JohnPhosAu(NCMe)]SbF_6 (**A**) (5 mol%, 20 µmol, 15.4 mg) was added. The mixture was stirred at room temperature for 5 hours. After removing the solvent in vacuo, the residue was adsorbed onto Celite® and purified by flash chromatography (SiO_2, *n*-hexane/Et_2O 98:2) to yield **12i** (83.6 mg, 269 µmol, 67%) as a pale yellow solid. M.p.: 56–58 °C. R_f (*n*-hexane/Et_2O 98:2) = 0.21. ^{1}H NMR (600 MHz, $CDCl_3$): δ = 7.56–7.50 (m, 2H, CH_{ar}), 7.37–7.30 (m, 5H, CH_{ar}), 6.93–6.88 (m, 2H, CH_{ar}), 3.84 (s, 3H, OCH_3), 2.29–2.24 (m, 2H, CH_2), 1.71–1.64 (m, 2H, CH_2), 0.90 ppm (t, $^3J_{H\text{-}H}$ = 7.4 Hz, 3H, CH_3). ^{13}C NMR (151 MHz, $CDCl_3$): δ = 159.9 ($C_{ar}OCH_3$), 135.7 (C=*C*Cl), 131.8 (C_{ar}H), 130.5 (C_{ar}H), 130.4 (C_{ar}), 128.50 (C_{ar}H), 128.46 (C_{ar}H), 123.5 (C_{ar}), 122.5 (*C*=CCl), 113.8 (C_{ar}H), 96.3 (C≡C), 88.4 (C≡C), 55.5 (OCH_3), 35.7 (CH_2), 22.3 (CH_2), 13.8 ppm (CH_3). IR (ATR): $\tilde{\nu}$ = 3065 (vw), 2961 (vw), 2930 (vw), 2874 (vw), 2841 (vw), 2089 (vw), 1601 (w), 1252 (w), 1173 (w), 1028 (w), 831 (w), 758 (m), 691 cm^{-1} (w). UV/Vis (CH_3CN): λ_{max} (log ε) = 299 nm (4.44). HRMS (ESI): $[C_{20}H_{19}{}^{35}ClO+H]^+$: calculated: 311.1197; observed: 311.1196.

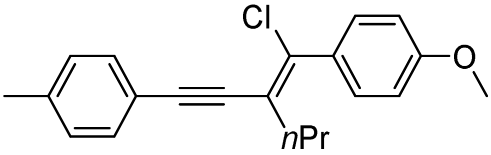

12j

Synthesis of enyne 12j. According to the general procedure, chloroarylacetylene **1e** (60.2 mg, 400 µmol, 1.0 eq) and 1-methoxy-4-(pent-1-yn-1-yl)benzene (**11d**) (139.4 mg, 800 µmol, 2.0 eq) were dissolved in dry DCE (4 mL). Then [JohnPhosAu(NCMe)]SbF_6 (**A**) (5 mol%, 20 µmol, 15.4 mg) was added. The mixture was stirred at room temperature for 3 hours. After removing the solvent in vacuo, the residue was adsorbed onto Celite® and purified by flash chromatography (SiO_2, *n*-hexane/Et_2O 99:1) to yield **12j** (97.0 mg, 299 µmol, 75%) as a white solid. M.p.: 93–95 °C. R_f (*n*-hexane/Et_2O 97:3) = 0.30. ^{1}H NMR (600 MHz, $CDCl_3$): δ = 7.44–7.40 (m, 2H, CH_{ar}), 7.33–7.29 (m, 2H, CH_{ar}), 7.16–7.13 (m, 2H, CH_{ar}), 6.92–6.89 (m, 2H, CH_{ar}), 3.84 (s, 3H, OCH_3), 2.37 (s, 3H,

$C_{ar}CH_3$), 2.28–2.24 (m, 2H, CH_2), 1.70–1.63 (m, 2H, CH_2), 0.89 ppm (t, $^3J_{H\text{-}H}$ = 7.5 Hz, 3H, CH_3). ^{13}C NMR (151 MHz, $CDCl_3$): δ = 159.9 ($\mathit{C}_{ar}OCH_3$), 138.7 ($\mathit{C}_{ar}CH_3$), 135.2 (C=*C*Cl), 131.7 (C_{ar}H), 130.5 (C_{ar}H), 130.4 (C_{ar}), 129.2 (C_{ar}H), 122.6 (*C*=CCl), 120.4 (C_{ar}), 113.8 (C_{ar}H), 96.5 (C≡C), 87.7 (C≡C), 55.5 (OCH_3), 35.8 (CH_2), 22.3 (CH_2), 21.7 ($C_{ar}\mathit{C}H_3$), 13.8 ppm (CH_3). IR (ATR): $\tilde{\nu}$ = 3067 (vw), 3032 (vw), 3003 (vw), 2953 (vw), 2928 (vw), 2868 (vw), 2837 (vw), 2195 (vw), 1593 (vw), 1503 (vw), 1246 (vw), 1175 (vw), 829 cm^{-1} (vw). UV/Vis (CH_3CN): λ_{max} (log ε) = 302 nm (4.31). HRMS (ESI): $[C_{21}H_{21}{}^{35}ClO+H]^+$: calculated: 325.1354; observed: 325.1352.

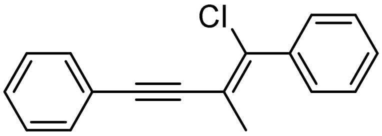

12k

Synthesis of enyne 12k. According to the general procedure, chloroarylacetylene **1d** (54.6 mg, 400 µmol, 1.0 eq) and 1-phenyl-1-propyne (**11a**) (92.9 mg, 800 µmol, 2.0 eq) were dissolved in dry DCE (4 mL). Then [JohnPhosAu(NCMe)]SbF_6 (**A**) (5 mol%, 20 µmol, 15.4 mg) was added. The mixture was stirred at room temperature for 52 hours. After removing the solvent in vacuo, the residue was adsorbed onto Celite® and purified by flash chromatography (SiO_2, *n*-hexane/Et_2O 200:1) to yield **12k** (41.5 mg, 164 µmol, 41%) as a colorless oil. R_f (*n*-hexane/Et_2O 99:1) = 0.28. ^{1}H NMR (600 MHz, $CDCl_3$): δ = 7.56–7.50 (m, 2H, CH_{ar}), 7.43–7.38 (m, 4H, CH_{ar}), 7.37–7.32 (m, 4H, CH_{ar}), 2.03 ppm (s, 3H, CH_3). ^{13}C NMR (151 MHz, $CDCl_3$): δ = 137.8 (C_{ar}), 135.5 (C=*C*Cl), 131.8 (C_{ar}H), 129.1 (C_{ar}H), 128.9 (C_{ar}H), 128.6 (C_{ar}H), 128.5 (C_{ar}H), 128.4 (C_{ar}H), 123.3 (C_{ar}), 117.7 (*C*=CCl), 95.8 (C≡C), 89.3 (C≡C), 21.0 ppm (CH_3). IR (ATR): $\tilde{\nu}$ = 3078 (vw), 3055 (vw), 3030 (vw), 3019 (vw), 2997 (vw), 2953 (vw), 2920 (vw), 2855 (vw), 2185 (vw), 1597 (vw), 1485 (w), 1441 (w), 1018 (w), 752 (m), 727 (w), 689 cm^{-1} (m). UV/Vis (CH_3CN): λ_{max} (log ε) = 296 nm (4.64). HRMS (EI): $[C_{17}H_{13}{}^{35}Cl]^+$: calculated: 252.0700; observed: 252.0704.

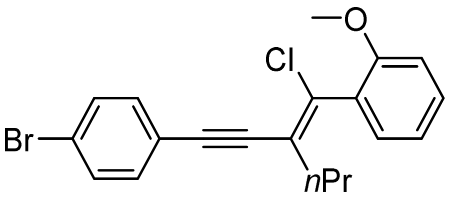

12l

Synthesis of enyne 12l. According to the general procedure, chloroarylacetylene **1b** (86.2 mg, 400 µmol, 1.0 eq) and 1-methoxy-2-(pent-1-yn-1-yl)benzene (**11f**) (69.7 mg, 400 µmol, 1.0 eq) were dissolved in dry DCE (4 mL). Then [JohnPhosAu(NCMe)]SbF_6 (**A**) (5 mol%, 20 µmol, 15.4 mg) was added. The mixture was stirred at room temperature for 20 hours. After removing the solvent in vacuo, the residue was adsorbed onto Celite® and purified by flash chromatography (SiO_2, *n*-hexane/Et_2O 97:3) to yield **12l** (127.1 mg, 326 µmol, 82%) as a pale yellow solid. M.p.: 70–72 °C. R_f (*n*-hexane/Et_2O 97:3) = 0.34. ^{1}H NMR (600 MHz, $CDCl_3$): δ = 7.49–7.45 (m, 2H, CH_{ar}), 7.41–7.37 (m, 2H, CH_{ar}), 7.37–7.33 (m, 1H, CH_{ar}), 7.23–7.20 (m, 1H, CH_{ar}), 7.00–6.96 (m, 1H, CH_{ar}), 6.95–6.92 (m, 1H, CH_{ar}), 3.85 (s, 3H, OCH_3), 2.14–1.98 (m, 2H, CH_2), 1.63–1.55 (m, 2H, CH_2), 0.84 ppm (t, $^3J_{H,H}$ = 7.5 Hz, 3H, CH_3). ^{13}C NMR (151 MHz, $CDCl_3$): δ = 156.6 ($C_{ar}OCH_3$), 133.2 ($C_{ar}H$), 132.7 (C=*C*Cl), 131.7 ($C_{ar}H$), 130.65 ($C_{ar}H$), 130.59 ($C_{ar}H$), 126.7 (*C*=CCl), 124.1 (C_{ar}), 122.7 ($C_{ar}Br$), 122.5 (C_{ar}), 120.6 ($C_{ar}H$), 111.4 ($C_{ar}H$), 94.8 (C≡C), 89.0 (C≡C), 55.8 (OCH_3), 35.7 (CH_2), 21.7 (CH_2), 13.7 ppm (CH_3). 3055 (vw), 3020 (vw), 2958 (vw), 2929 (vw), 2870 (vw), 2841 (vw), 1591 (vw), 1578 (vw),1483 (w), 1455 (w), 1435 (w), 1286 (w), 1249 (m), 1009 (w), 914 (w), 823 (m), 761 cm^{-1} (m). UV/Vis (CH_3CN): λ_{max} (log ε) = 289 nm (4.38). HRMS (ESI): $[C_{20}H_{18}{}^{79}Br^{35}ClO+H]^+$: calculated: 389.0302; observed: 389.0297.

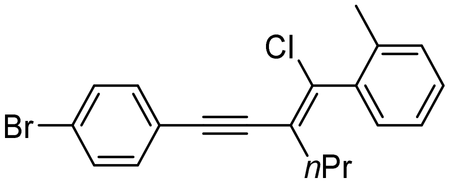

12m

Synthesis of enyne 12m. According to the general procedure, chloroarylacetylene **1b** (86.2 mg, 400 µmol, 1.0 eq) and 1-methyl-2-(pent-1-yn-1-yl)benzene (**11g**) (63.3 mg, 400 µmol, 1.0 eq) were dissolved in dry DCE (4 mL). Then [JohnPhosAu(NCMe)]SbF_6 (**A**) (5 mol%, 20 µmol, 15.4 mg) was added. The mixture was stirred at 50 °C for 40 hours. After removing the solvent in vacuo, the residue was adsorbed onto Celite® and purified by flash chromatography (SiO_2, *n*-hexane) to yield **12m** (80.0 mg, 214 µmol, 54%) as a pale yellow oil. R_f (*n*-hexane/Et_2O 97:3) = 0.62. ^{1}H NMR (600

MHz, $CDCl_3$): δ = 7.51–7.45 (m, 2H, CH_{ar}) 7.42–7.37 (m, 2H, CH_{ar}), 7.30–7.26 (m, 1H, CH_{ar}), 7.26–7.23 (m, 1H, CH_{ar}), 7.23–7.19 (m, 1H, CH_{ar}), 7.19–7.15 (m, 1H, CH_{ar}), 2.33 (s, 3H, $C_{ar}CH_3$), 2.08–1.97 (m, 2H, CH_2), 1.62–1.55 (m, 2H, CH_2), 0.84 ppm (t, $^3J_{H,H}$ = 7.6 Hz, 3H, CH_3). ^{13}C NMR (151 MHz, $CDCl_3$): δ = 137.3 (C_{ar}), 136.5 ($\mathit{C}_{ar}CH_3$), 135.6 (C=*C*Cl), 133.3 (C_{ar}H), 131.8 (C_{ar}H), 130.5 (C_{ar}H), 129.2 (C_{ar}H), 129.1 (C_{ar}H), 126.1 (C_{ar}H), 123.6 (*C*=CCl), 122.9 (C_{ar}Br), 122.3 (C_{ar}), 94.8 (C≡C), 88.7 (C≡C), 35.4 (CH_2), 21.8 (CH_2), 19.5 ($C_{ar}\mathit{C}H_3$), 13.7 ppm (CH_3). IR (ATR): $\tilde{\nu}$ = 3063 (vw), 3018 (vw), 2959 (vw), 2928 (vw), 2870 (vw), 1482 (w), 1456 (vw), 1392 (vw), 1069 (w), 1009 (w), 913 (w), 821 (m), 748 (m), 734 cm^{-1} (m). UV/Vis (CH_3CN): λ_{max} (log ε) = 288 (4.43), 303 nm (4.37). HRMS (EI): $[C_{20}H_{18}{}^{79}Br^{35}Cl]^+$: calculated: 372.0275; observed: 372.0266.

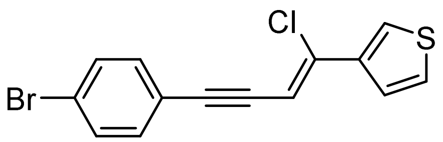

12n

Synthesis of enyne 12n. According to the general procedure, chloroarylacetylene **1b** (86.2 mg, 400 µmol, 1.0 eq) and 3-ethynylthiophene (86.6 mg, 800 µmol, 2.0 eq) were dissolved in dry DCE (4 mL). Then [JohnPhosAu(NCMe)]SbF_6 (**A**) (5 mol%, 20 µmol, 15.4 mg) was added. The mixture was stirred at 70 °C for 4 days. After removing the solvent in vacuo, the residue was adsorbed onto Celite® and purified by flash chromatography (SiO_2, *n*-hexane/Et_2O 99:1) to yield **12n** (30.7 mg, 95 µmol, 24%) as a pale yellow solid. M.p.: 120–122 °C. R_f (*n*-hexane/Et_2O 97:3) = 0.43. ^{1}H NMR (600 MHz, $CDCl_3$): δ = 7.64–7.60 (m, 1H, CH_{ar}), 7.50–7.46 (m, 2H, CH_{ar}), 7.39–7.36 (m, 2H, CH_{ar}), 7.35–7.33 (m, 1H, CH_{ar}), 7.30–7.28 (m, 1H, CH_{ar}), 6.37 ppm (s, 1H, C=CH). ^{13}C NMR (151 MHz, $CDCl_3$): δ = 138.8 (C=*C*Cl), 137.8 (C_{ar}), 133.2 (C_{ar}H), 131.8 (C_{ar}H), 127.1 (C_{ar}H), 125.2 (C_{ar}H), 124.6 (C_{ar}H), 123.0 (C_{ar}Br), 122.2 (C_{ar}), 105.6 (C=*C*H), 97.2 (C≡C), 87.1 ppm (C≡C). IR (ATR): $\tilde{\nu}$ = 3104 (vw), 3029 (vw), 1568 (vw), 1478 (vw), 1241 (vw), 1069 (vw), 1007 (vw),820 (w), 779 (m), 633 cm^{-1} (w). UV/Vis (CH_3CN): λ_{max} (log ε) = 251 (3.83), 321 nm (4.13). HRMS (EI): $[C_{14}H_8{}^{79}Br^{35}ClS]^+$: calculated: 321.9213; observed: 321.9210.

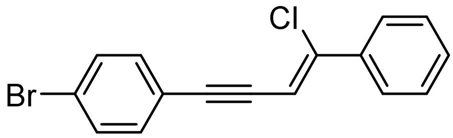

12o

Synthesis of enyne 12o. According to the general procedure, chloroarylacetylene **1b** (86.2 mg, 400 µmol, 1.0 eq) and phenylacetylene (81.7 mg, 800 µmol, 2.0 eq) were dissolved in dry DCE (4 mL). Then [JohnPhosAu(NCMe)]SbF_6 (**A**) (5 mol%, 20 µmol, 15.4 mg) was added. The mixture was stirred at 50°C for 40 hours. After removing the solvent in vacuo, the residue was adsorbed onto Celite® and purified by flash chromatography (SiO_2, *n*-hexane/Et_2O 99:1) to yield **12o** (60.0 mg, 189 µmol, 47%) as a white solid. M.p.: 112–114 °C. R_f (*n*-hexane/Et_2O 97:3) = 0.52. ^{1}H NMR (600 MHz, $CDCl_3$): δ = 7.68–7.64 (m, 2H, CH_{ar}), 7.51–7.47 (m, 2H, CH_{ar}), 7.42–7.37 (m, 5H, CH_{ar}), 6.43 ppm (C=CH). ^{13}C NMR (151 MHz, $CDCl_3$): δ = 143.2 (C=*C*Cl), 136.6 (C_{ar}), 133.2 (C_{ar}H), 131.8 (C_{ar}H), 129.9 (C_{ar}H), 128.7 (C_{ar}H), 126.5 (C_{ar}H), 123.1 (C_{ar}Br), 122.2 (C_{ar}), 106.9 (C=*C*H), 96.8 (C≡C), 87.3 ppm (C≡C). IR (ATR): $\tilde{\nu}$ = 3058 (vw), 3032 (vw), 1482 (vw), 1472 (vw), 1444 (vw), 1391 (vw), 1070 (w), 1007 (w), 822 (w), 757 (m), 685 (w), 671 cm^{-1} (w). UV/Vis (CH_3CN): λ_{max} (log ε) = 247 (4.07), 314 nm (4.32). HRMS (EI): $[C_{16}H_{10}{}^{79}Br^{35}Cl]^+$: calculated: 315.9649; observed: 315.9643.

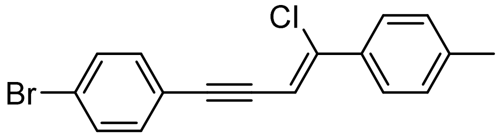

12p

Synthesis of enyne 12p. According to the general procedure, chloroarylacetylene **1b** (86.2 mg, 400 µmol, 1.0 eq) and 1-ethynyl-4-methylbenzene (92.9 mg, 800 µmol, 2.0 eq) were dissolved in dry DCE (4 mL). Then [JohnPhosAu(NCMe)]SbF_6 (**A**) (5 mol%, 20 µmol, 15.4 mg) was added. The mixture was stirred at 50°C for 40 hours. After removing the solvent in vacuo, the residue was adsorbed onto Celite® and purified by flash chromatography (SiO_2, *n*-hexane/Et_2O 99:1) to yield **12p** (92.7 mg, 280 µmol, 70%) as a white solid. M.p.: 137–139 °C. R_f (*n*-hexane/Et_2O 97:3) = 0.53. ^{1}H NMR (600 MHz, $CDCl_3$): δ = 7.57–7.54 (m, 2H, CH_{ar}), 7.49–7.46 (m, 2H, CH_{ar}), 7.40–7.36 (m, 2H, CH_{ar}), 7.21–7.18 (m, 2H, CH_{ar}), 6.39 (s, 1H, C=CH), 2.38 ppm (s, 3H, CH_3). ^{13}C NMR (151 MHz, $CDCl_3$): δ = 143.3 (C=*C*Cl), 140.2 (*C*$_{ar}$$CH_3$), 133.9 ($C_{ar}$) , 133.2 ($C_{ar}$H), 131.8 ($C_{ar}$H), 129.4 ($C_{ar}$H), 126.4 ($C_{ar}$H), 123.0 ($C_{ar}$Br), 122.3 ($C_{ar}$), 105.9 (C=*C*H), 96.5 (C≡C), 87.5 (C≡C),

21.4 ppm (CH_3). IR (ATR): $\tilde{\nu}$ = 3086 (vw), 3028 (vw), 2912 (vw), 2854 (vw), 1910 (vw), 1587 (vw), 1481 (w), 1069 (w), 1008 (w), 915 (w), 823 (w), 807 (m), 665 (w), 633 cm^{-1} (w). UV/Vis (CH_3CN): λ_{max} (log ε) = 252 (4.04), 318 nm (4.24). HRMS (EI): $[C_{17}H_{12}{}^{79}Br^{35}Cl]^+$: calculated: 329.9805; observed: 329.9810.

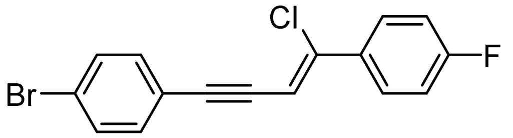

12q

Synthesis of enyne 12q. According to the general procedure, chloroarylacetylene **1b** (86.2 mg, 400 µmol, 1.0 eq) and 1-ethynyl-4-fluorobenzene (96.1 mg, 800 µmol, 2.0 eq) were dissolved in dry DCE (4 mL). Then $[JohnPhosAu(NCMe)]SbF_6$ (**A**) (5 mol%, 20 µmol, 15.4 mg) was added. The mixture was stirred at 50°C for 40 hours. After removing the solvent in vacuo, the residue was adsorbed onto Celite® and purified by flash chromatography (SiO_2, *n*-hexane/Et_2O 99:1) to yield **12q** (92.5 mg, 277 µmol, 69%) as a pale yellow solid. M.p.: 94–96 °C. R_f (*n*-hexane/Et_2O 97:3) = 0.47. ^1H NMR (600 MHz, $CDCl_3$): δ = 7.66–7.62 (m, 2H, CH_{ar}), 7.50–7.47 (m, 2H, CH_{ar}), 7.40–7.36 (m, 2H, CH_{ar}), 7.11–7.06 (m, 2H, CH_{ar}), 6.36 ppm (s, 1H, C=CH). ^{19}F NMR (565 Hz): −110.80 ppm (m, CF_{ar}). ^{13}C NMR (151 MHz, $CDCl_3$): δ = 163.7 (d, $^1J_{C\text{-}F}$ = 250.9 Hz, $C_{ar}F$), 142.0 (C=*C*Cl), 133.2 ($C_{ar}H$), 132.9 (d, $^4J_{C\text{-}F}$ = 3.5 Hz, C_{ar}), 131.8 ($C_{ar}H$), 128.4 (d, $^3J_{C\text{-}F}$ = 9.3 Hz, $C_{ar}H$), 123.2 ($C_{ar}Br$), 122.1 (C_{ar}), 115.8 (d, $^2J_{C\text{-}F}$ = 22.0 Hz, CarH), 106.8 (C=*C*H), 96.9 (C≡C), 87.1 (C≡C) ppm. IR (ATR): $\tilde{\nu}$ = 3035 (vw), 1592 (vw), 1505 (w), 1230 (w), 1160 (w), 1070 (w), 1009 (w), 838 (w), 812 (m), 664 cm^{-1} (w). UV/Vis (CH_3CN): λ_{max} (log ε) = 248 (4.19), 314 nm (4.29). HRMS (EI): $[C_{16}H_9{}^{79}Br^{35}ClF]^+$: calculated: 333.9555; observed: 333.9549.

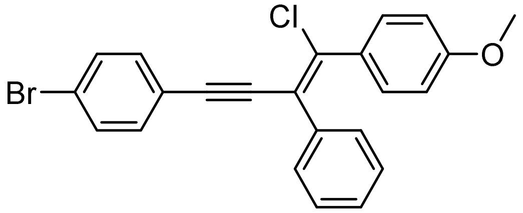

12r

Synthesis of enyne 12r. According to the general procedure, chloroarylacetylene **1b** (86.2 mg, 400 µmol, 1.0 eq) and 1-methoxy-4-(phenylethynyl)benzene (166.6 mg, 800 µmol, 2.0 eq) were

dissolved in dry DCE (4 mL). Then [JohnPhosAu(NCMe)]SbF_6 (**A**) (5 mol%, 20 µmol, 15.4 mg) was added. The mixture was stirred at 50°C for 40 hours. After removing the solvent in vacuo, the residue was adsorbed onto Celite® and purified by flash chromatography (SiO_2, *n*-hexane/Et_2O 97:3) to yield **12r** (85.1 mg, 201 µmol, 50%) as a pale yellow solid. M.p.: 167–169 °C. R_f (*n*-hexane/Et_2O 97:3) = 0.30. ^{1}H NMR (400 MHz, C_6D_6): δ = 7.34–7.29 (m, 2H, CH_{ar}), 7.20–7.18 (m, 2H, CH_{ar}), 7.10–7.04 (m, 4H, CH_{ar}), 6.99–6.90 (m, 3H, CH_{ar}), 6.46–6.40 (m, 2H, CH_{ar}), 3.08 ppm (s, 3H, OCH_3). ^{13}C NMR (101 MHz, C_6D_6): δ = 160.4 ($C_{ar}OCH_3$), 139.3 (C=*C*Cl), 138.2 (C_{ar}), 133.4 ($C_{ar}H$), 131.9 ($C_{ar}H$), 131.8 ($C_{ar}H$), 130.1 (C_{ar}), 130.0 ($C_{ar}H$), 128.6 ($C_{ar}H$), 127.9 ($C_{ar}H$), 123.1 (C_{ar}), 122.6 (C_{ar}), 122.2 (C_{ar}), 113.8 ($C_{ar}H$), 95.9 (C≡C), 91.6 (C≡C), 54.7 ppm (OCH_3). IR (ATR): $\tilde{\nu}$ = 3078 (vw), 3054 (vw), 3009 (vw), 2961 (vw), 2930 (vw), 2835 (vw), 1602 (w), 1505 (vw), 1482 (w), 1440 (vw), 1248 (m), 1174 (w), 1025 (vw), 1011 (vw), 822 (m), 770 (w), 700 (w), 653 cm^{-1} (w). UV/Vis (CH_3CN): λ_{max} (log ε) = 267 (4.40), 329 nm (4.30). HRMS (ESI): $[C_{23}H_{16}{}^{79}Br^{35}ClO+H]^+$: calculated: 423.0146; observed: 423.0126.

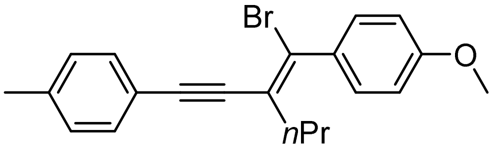

13a

Synthesis of enyne 13a. According to the general procedure, bromoarylacetylene **4e** (78.0 mg, 400 µmol, 1.0 eq) and 1-methoxy-4-(pent-1-yn-1-yl)benzene (**11d**) (139.4 mg, 800 µmol, 2.0 eq) were dissolved in dry DCE (4 mL). Then [JohnPhosAu(NCMe)]SbF_6 (**A**) (5 mol%, 20 µmol, 15.4 mg) was added. The mixture was stirred at room temperature for 19 hours. After removing the solvent in vacuo, the residue was adsorbed onto Celite® and purified by flash chromatography (SiO_2, *n*-hexane/Et_2O 97:3) to yield **13a** (122.4 mg, 331 µmol, 83%) as a white solid. M.p.: 98–100 °C. R_f (*n*-hexane/Et_2O 97:3) = 0.27. ^{1}H NMR (600 MHz, $CDCl_3$): δ = 7.46–7.40 (m, 2H, CH_{ar}), 7.30–7.26 (m, 2H, CH_{ar}), 7.17–7.13 (m, 2H, CH_{ar}), 6.91–6.87 (m, 2H, CH_{ar}), 3.83 (s, 3H, OCH_3), 2.37 (s, $C_{ar}CH_3$), 2.24–2.19 (m, 2H, CH_2), 1.70–1.63 (m, 2H, CH_2), 0.88 ppm (t, $^3J_{H\text{-}H}$ = 7.5 Hz, 3H, CH_3). ^{13}C NMR (151 MHz, $CDCl_3$): δ = 159.7 ($C_{ar}OCH_3$), 138.7 ($C_{ar}CH_3$), 132.1 (C_{ar}), 131.7 ($C_{ar}H$), 130.6 ($C_{ar}H$), 129.2 ($C_{ar}H$), 126.6 (C=*C*Br), 126.3 (*C*=CBr), 120.3 (C_{ar}), 113.7 ($C_{ar}H$), 95.7 (C≡C), 89.7 (C≡C), 55.5 (OCH_3), 36.3 (CH_2), 22.2 (CH_2), 21.7 ($C_{ar}CH_3$), 13.7 ppm (CH_3). IR (ATR): $\tilde{\nu}$ = 3069 (vw), 3003 (vw), 2955 (vw), 2928 (vw), 2868 (vw), 2837 (w), 2116 (vw), 2085 (vw), 1593 (vw), 1501 (vw), 1246 (w), 1175 (vw), 1030 (vw), 826 (w), 812 cm^{-1} (w). UV/Vis

(CH_3CN): λ_{max} (log ε) = 303 nm (4.48). HRMS (ESI): [$C_{20}H_{18}{}^{79}Br^{35}ClO+H]^+$: calculated: 369.0849; observed: 369.0844.

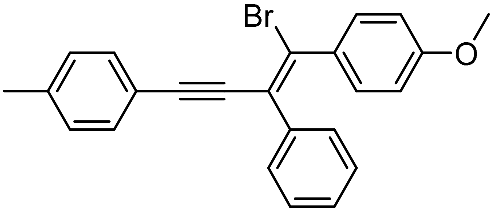

13b

Synthesis of enyne 13b. According to the general procedure, bromoarylacetylene **4e** (78.0 mg, 400 µmol, 1.0 eq) and 1-methoxy-4-(phenylethynyl)benzene (166.6 mg, 800 µmol, 2.0 eq) were dissolved in dry DCE (4 mL). Then [JohnPhosAu(NCMe)]SbF_6 (**A**) (5 mol%, 20 µmol, 15.4 mg) was added. The mixture was stirred at room temperature for 41 hours. After removing the solvent in vacuo, the residue was adsorbed onto Celite® and purified by flash chromatography (SiO_2, *n*-hexane/Et_2O 97:3) to yield **13b** (85.2 mg, 211 µmol, 53%) as a pale yellow solid. M.p.: 169–171 °C. R_f (*n*-hexane/Et_2O 97:3) = 0.36. ^{1}H NMR (400 MHz, $CDCl_3$): δ = 7.56–7.50 (m, 2H, CH_{ar}), 7.37–7.33 (m, 2H, CH_{ar}), 7.31–7.21 (m, 7H, CH_{ar}), 6.82–6.77 (m, 2H, CH_{ar}), 3.85 (s, 3H, OCH_3), 2.45 ppm (s, 3H, CH_3). ^{13}C NMR (101 MHz, $CDCl_3$): δ = 159.7 ($C_{ar}OCH_3$), 138.9 ($C_{ar}CH_3$), 138.2 (C_{ar}), 131.9 (C_{ar}), 131.8 ($C_{ar}H$), 131.7 ($C_{ar}H$), 129.8 (C=*C*Br), 129.6 ($C_{ar}H$), 129.2 ($C_{ar}H$), 128.3 ($C_{ar}H$), 127.6 ($C_{ar}H$), 125.5 (*C*=CBr), 120.2 (C_{ar}), 113.5 ($C_{ar}H$), 96.0 (C≡C), 91.3 (C≡C), 55.4 (OCH_3), 21.7 ppm (CH_3). IR (ATR): $\tilde{\nu}$ = 3078 (vw), 3052 (vw), 3028 (vw), 3007 (vw), 2961 (vw), 2932 (vw), 2838 (vw), 2194 (vw), 1600 (w), 1500 (w), 1441 (w), 1248 (w), 1183 (w), 1026 (w), 832 (w), 813 (m), 761 (w), 701 (w), 621 cm^{-1} (w). UV/Vis (CH_3CN): λ_{max} (log ε) = 232 (4.35), 270 (4.20), 325 nm (4.30). HRMS (APCI): [$C_{24}H_{19}{}^{79}BrO+H]^+$: calculated: 403.0692; observed: 403.0656.

Preparation of ^{13}C-Labeled Enyne 12i

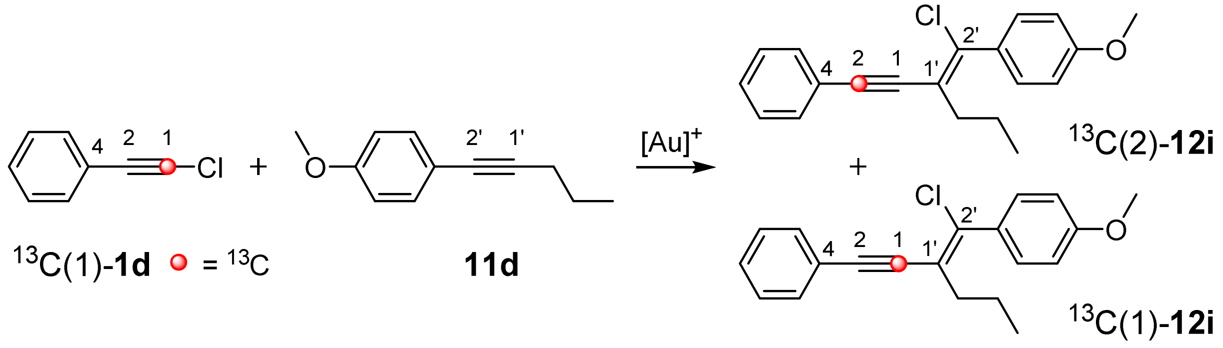

Synthesis of ^{13}C-labeled Enyne 12i. Chloroarylacetylene ^{13}C(1)-**1d** (54.6 mg, 400 µmol, 1.0 eq) and 1-methoxy-4-(pent-1-yn-1-yl)benzene (**11d**) (70.0 mg, 400 µmol, 1.0 eq) were dissolved in dry DCE (4 mL). Then [JohnPhosAu(NCMe)]SbF_6 (**A**) (5 mol%, 20 µmol, 15.4 mg) was added. The mixture was stirred at room temperature for 43 hours. After removing the solvent in vacuo, the residue was adsorbed onto Celite® and purified by flash chromatography (SiO_2, *n*-hexane/Et_2O 97:3) to yield a mixture of ^{13}C(2)-**12i** and ^{13}C(1)-**12i** (100.1 mg, 322 µmol, 81%) as a pale yellow solid. R_f (*n*-hexane/Et_2O 97:3) = 0.35. ^{1}H NMR (400 MHz, $CDCl_3$): δ = 7.56–7.49 (m, 2H, CH_{ar}), 7.38–7.29 (m, 5H, CH_{ar}), 6.93–6.89 (m, 2H, CH_{ar}), 3.84 (s, 3H, OCH_3), 2.30–2.23 (m, 2H, CH_2), 1.73–1.62 (m, 2H, CH_2), 0.90 ppm (t, $^3J_{H,H}$ = 7.4 Hz, 3H, CH_3). ^{13}C NMR (101 MHz, $CDCl_3$) ^{13}C(2)-**12i**: δ = 159.9 (C_{ar}OCH3), 135.7 (d, $^3J_{C2'-C2}$ = 4.4 Hz, C=*C*Cl), 131.8 (C_{ar}H), 130.5 (C_{ar}H), 130.4 (C_{ar}), 128.50 (C_{ar}H), 128.46 (C_{ar}H), 123.5 (d, $^1J_{C4-C2}$ = 90.9 Hz, C_{ar}), 122.5 (d, $^2J_{C1'-C2}$ = 11.4 Hz, *C*=CCl), 113.8 (C_{ar}H), 96.3 (d, $^1J_{C2-C1}$ = 182.7 Hz; d, $^1J_{C2-C4}$ = 90.9 Hz, C≡C), 88.4 (d, $^1J_{C1-C2}$ = 182.7 Hz, C≡C), 55.5 (OCH_3), 35.7 (CH_2), 22.3 (CH_2), 13.8 ppm (CH_3). ^{13}C(1)-**12i**: δ = 159.9 ($C_{ar}OCH_3$), 135.6 (d, $^2J_{C2'-C1}$ = 97.1 Hz, C=*C*Cl), 131.8 (C_{ar}H), 130.5 (C_{ar}H), 130.4 (C_{ar}), 128.50 (C_{ar}H), 128.46 (C_{ar}H), 123.5 (d, $^2J_{C4-C1}$ = 13.1 Hz, C_{ar}), 122.4 (d, $^1J_{C1'-C1}$ = 92.0 Hz, *C*=CCl), 113.8 (C_{ar}H), 96.3 (d, $^1J_{C2-C1}$ = 182.7 Hz, C≡C), 88.4 (d, $^1J_{C1-C2}$ = 182.7 Hz; d, $^1J_{C1-C1'}$ = 91.9 Hz, C≡C), 55.5 (OCH_3), 35.7 (CH_2), 22.3 (CH_2), 13.8 ppm (CH_3).

Preparation of Enediyne S3

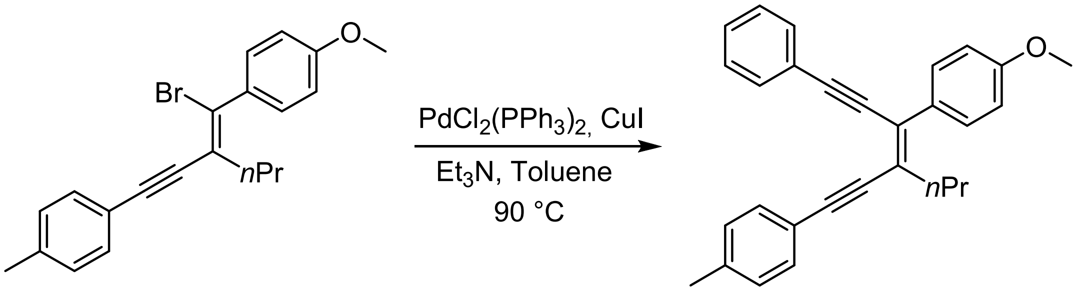

Scheme S1. The halogen atom of the obtained enynes can be used as reactive group in a subsequent cross coupling reaction.

Synthesis of Enediyne S3. A nitrogen flask was charged with enyne **13a** (51.8 mg, 0.140 mmol, 1.0 eq), CuI (4.0 mg, 0.021 mmol, 15 mol%) and $PdCl_2(PPh_3)_2$ (4.9 mg, 0.007 mmol, 5 mol%). The flask was evacuated and backfilled with argon. Then dry toluene (1 mL) and Et_3N (1 mL) were added. After the addition of phenylacetylene (21.4 mg, 0.210 mmol, 1.5 eq), the flask was sealed and the mixture was stirred at 90 °C for 3 days. After removing the solvent in vacuo, the residue was adsorbed onto Celite® and purified by flash chromatography (SiO_2, *n*-hexane/Et_2O 97:3) to yield **S3** (43.7 mg, 0.112 mmol, 80%) as a pale yellow solid. M.p.: 80–82 °C. R_f (*n*-hexane/Et_2O 97:3) = 0.23. ^{1}H NMR (400 MHz, $CDCl_3$): δ = 7.53–7.45 (m, 2H, CH_{ar}), 7.45–7.40 (m, 2H, CH_{ar}), 7.38–7.34 (m, 2H, CH_{ar}), 7.31–7.27 (m, 3H, CH_{ar}), 7.16–7.11 (m, 2H, CH_{ar}), 6.96–6.90 (m, 2H, CH_{ar}), 3.85 (s, 3H, OCH_3), 2.40–2.33 (m, 2H, CH_2), 2.36 (s, 3H, CH_3), 1.77–1.65 (m, 2H, CH_2), 0.93 ppm (t, $^3J_{H\text{-}H}$ = 7.5 Hz, 3H, CH_3). ^{13}C NMR (101 MHz, $CDCl_3$): δ = 159.3 ($C_{ar}OCH_3$), 138.6 (CarCH_3), 131.7 ($C_{ar}H$), 131.6 ($C_{ar}H$), 130.9 (C=$C$$CH_2$), 130.4 ($C_{ar}$), 130.3 ($C_{ar}H$), 129.3 ($C_{ar}H$), 128.4 ($C_{ar}H$), 128.3 ($C$=C$CH_2$), 128.2 ($C_{ar}H$), 123.9 ($C_{ar}$), 120.7 ($C_{ar}$), 113.8 ($C_{ar}H$), 96.9 (C≡C), 94.5 (C≡C), 91.6 (C≡C), 90.4 (C≡C), 55.5 (OCH_3), 35.0 ($C_{ar}CH_3$), 22.4 (CH_2), 21.7 (CH_2), 14.0 ppm (CH_3). IR (ATR): $\tilde{\nu}$ = 3053 (vw), 3030 (vw), 3010 (vw), 2957 (vw), 2925 (vw), 2869 (vw), 2834 (vw), 1605 (w), 1504 (w), 1246 (m), 1158 (w), 1027 (w), 837 (w), 819 (w), 760 (w), 696 cm^{-1} (w). UV/Vis (CH_3CN): λ_{max} (log ε) = 226 (4.11), 267 (4.09), 342 nm (4.11). HRMS (EI): $[C_{29}H_{26}O]^+$: calculated: 390.1978; observed: 390.1980.

3. NMR Experiments

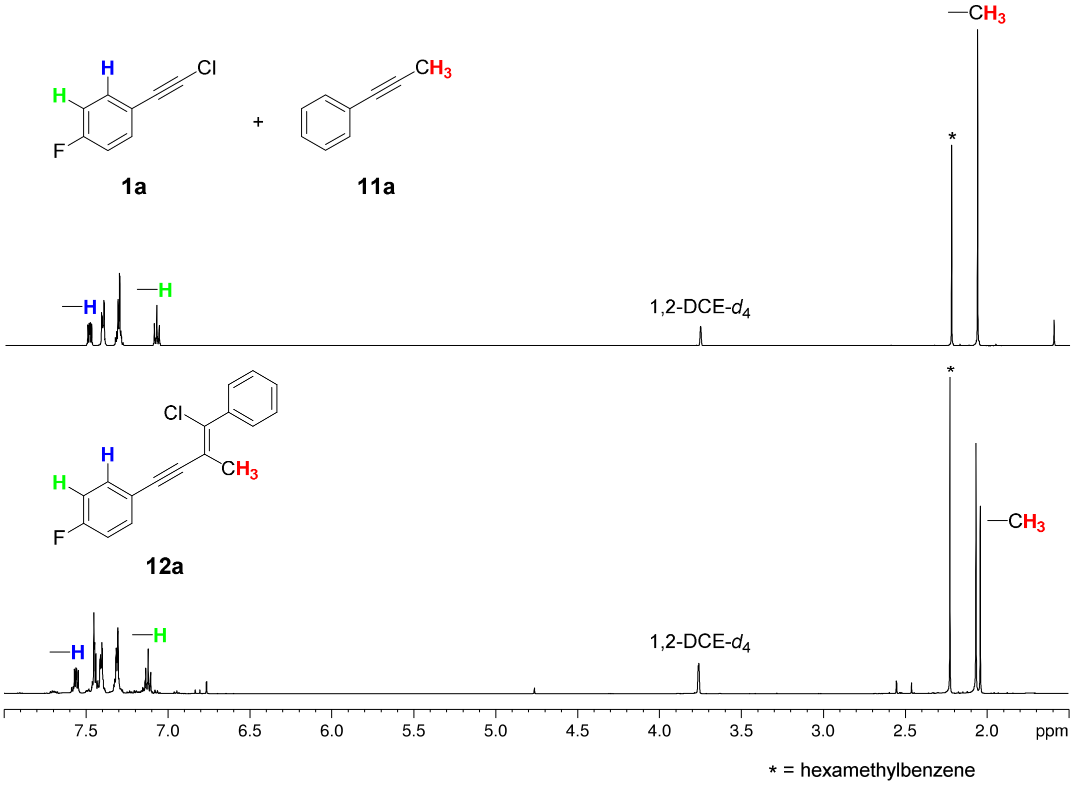

Figure S14. ^{1}H NMR spectra of chloroarylacetylene **1a** (0.1 M), 1-phenyl-1-propyne (**11a**) (2.0 eq) and hexamethylbenzene as internal standard in 1,2-dichloroethane-d_4 at 600 MHz before (top) and after (bottom) the addition of 5 mol% [JohnPhosAu(NCMe)]SbF_6 (**A**) at room temperature (12 hours reaction time).

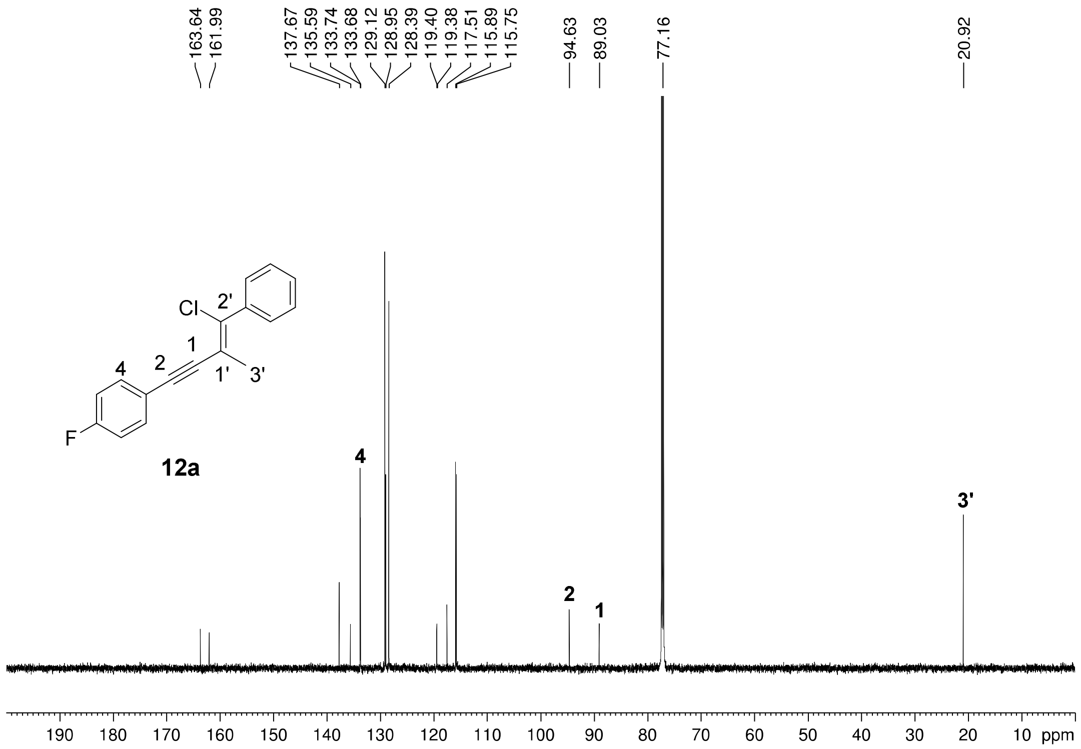

Figure S15. ^{13}C NMR spectrum of **12a** in $CDCl_3$ at 151 MHz. Some relevant carbon atoms are indicated.

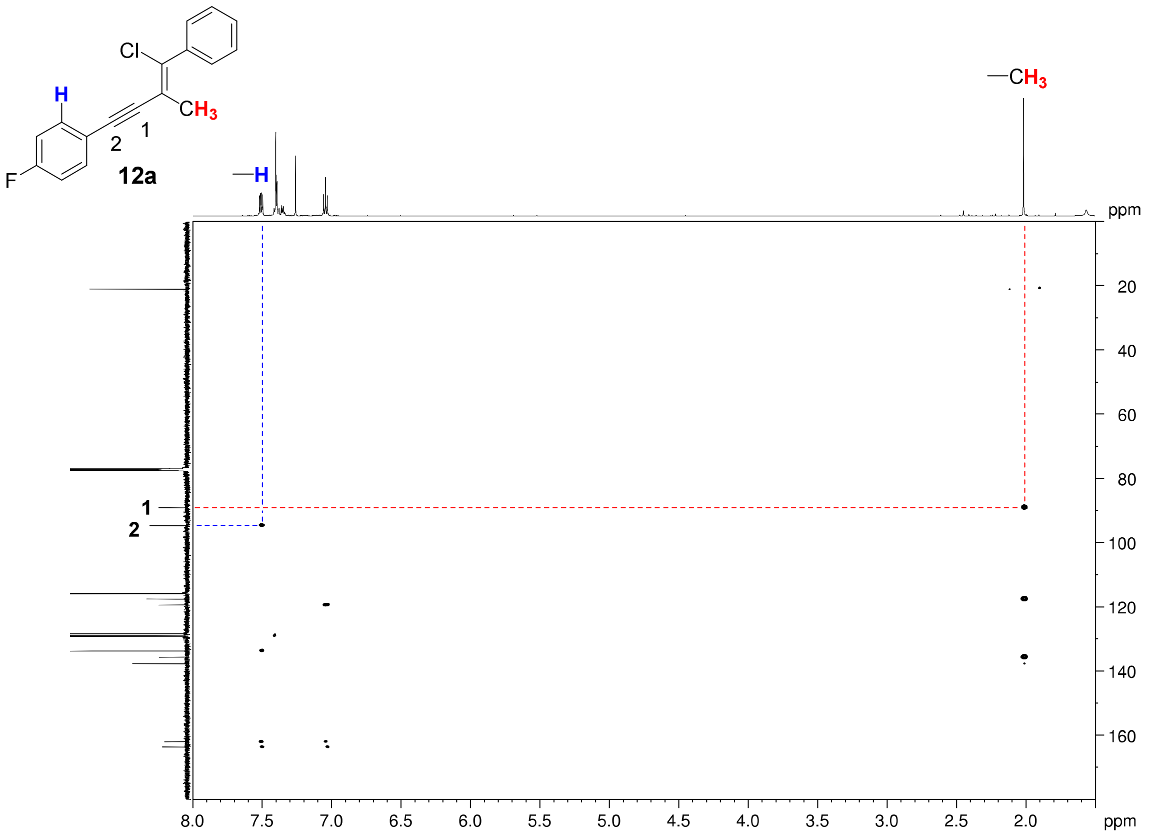

Figure S16. HMBC NMR spectrum of **12a** in $CDCl_3$ at 600 MHz. The correlation of the methyl protons (red) with the acetylenic carbon atom C1 and the correlation of the aromatic protons (blue) of the aryl group with the acetylenic carbon atom C2 are indicated.

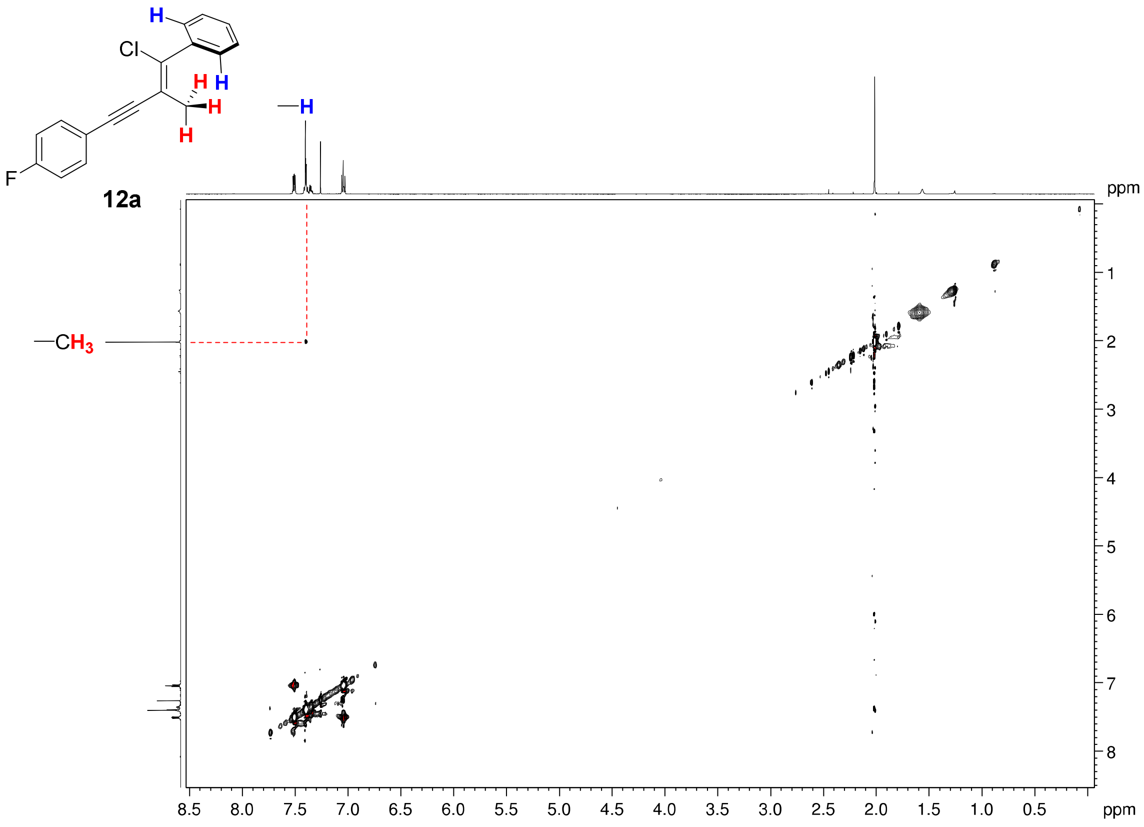

Figure S17. NOESY NMR spectrum of **12a** in $CDCl_3$ at 600 MHz. The correlation of the methyl protons (red) with the aromatic protons (blue) of the phenyl group is indicated.

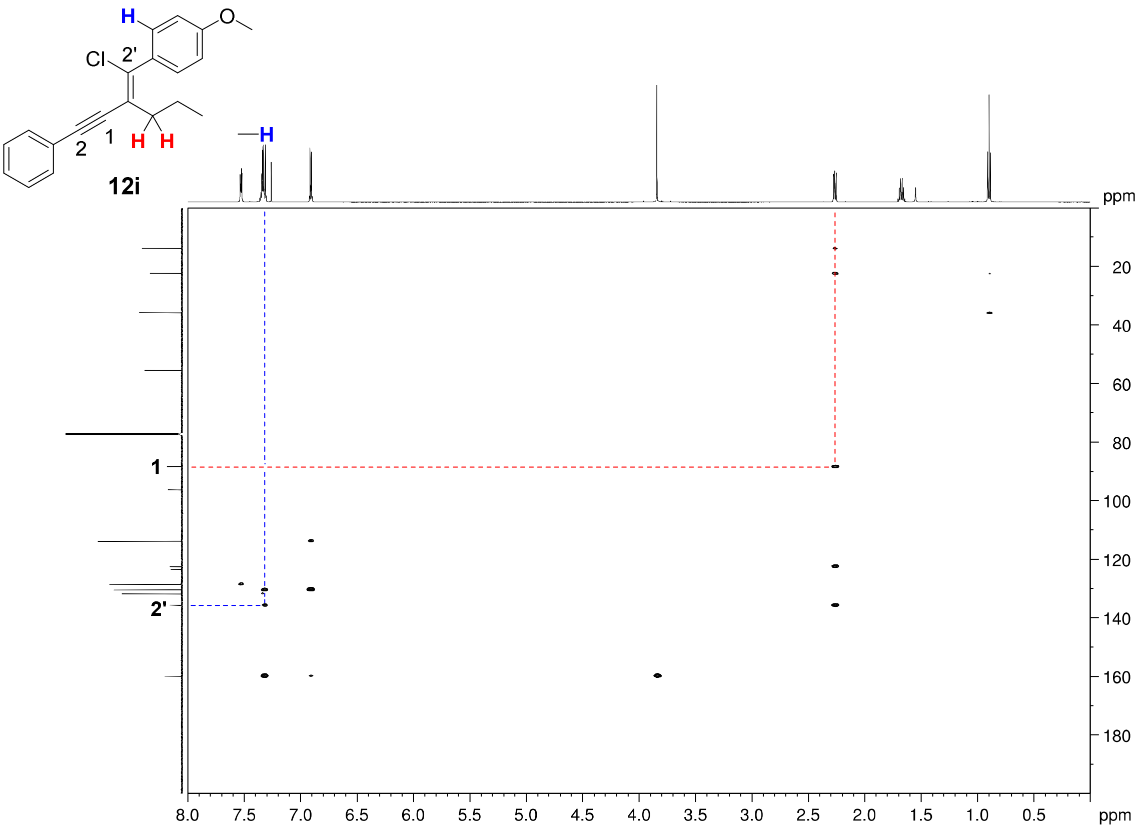

Figure S18. HMBC NMR spectrum of **12i** in $CDCl_3$ at 600 MHz. The correlation of the methylene protons (red) with the acetylenic carbon atom C1 and the correlation of the aromatic protons (blue) of the aryl group with the vinylic carbon atom C2' are indicated.

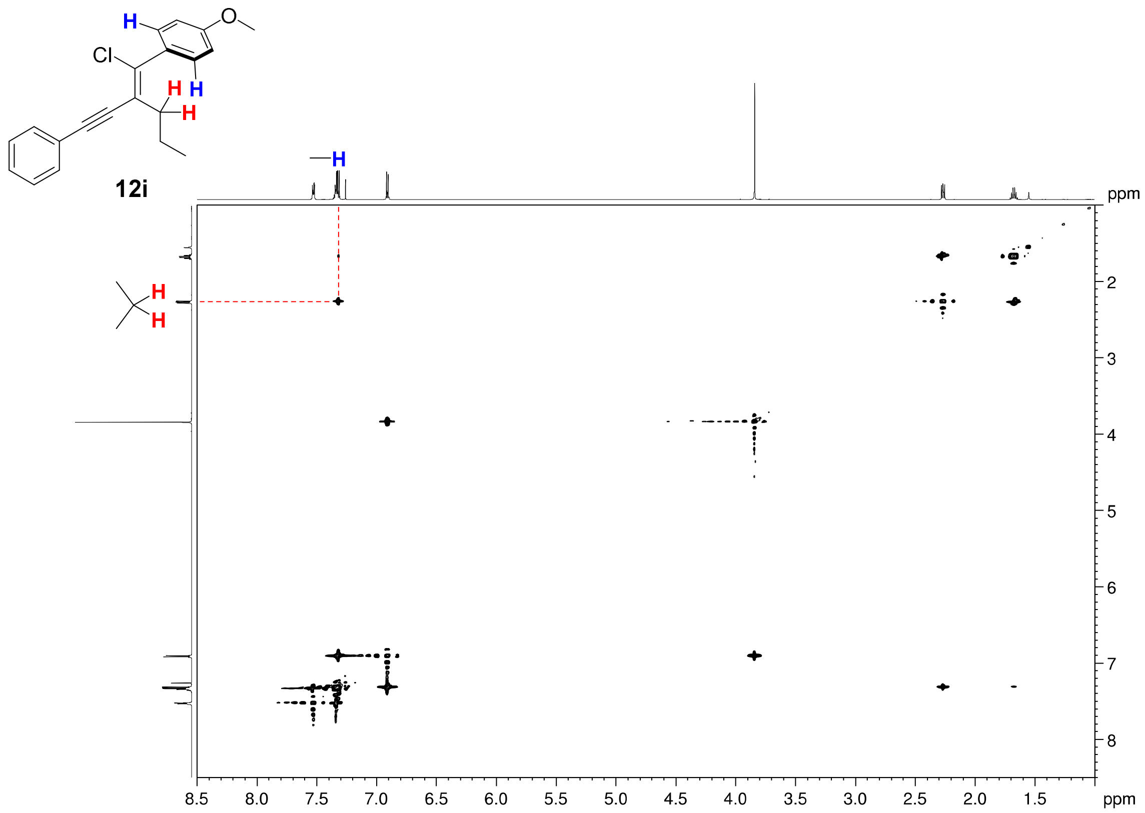

Figure S19. NOESY NMR spectrum of **12i** in $CDCl_3$ at 600 MHz. The correlation of the methylene protons (red) with the aromatic protons (blue) of the aryl group is indicated.

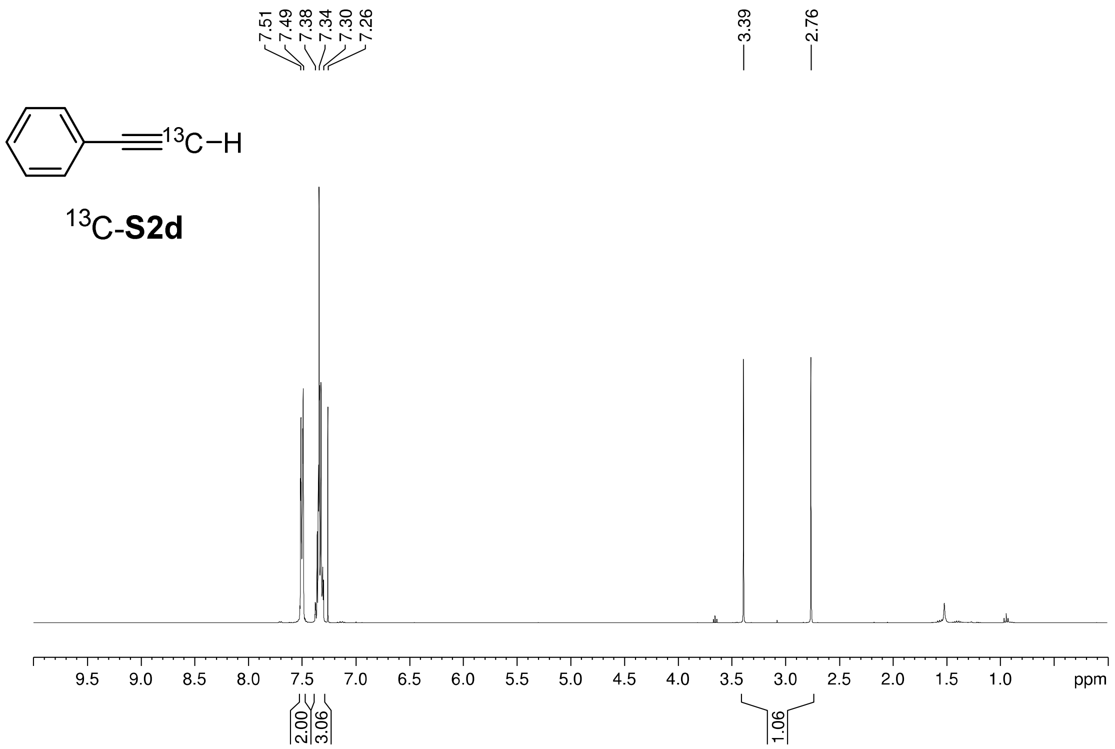

Figure S20. ^{1}H NMR spectrum of ^{13}C-**S2d** in $CDCl_3$ at 400 MHz. The doublet at δ = 3.03 ppm shows a coupling constant of J = 250 Hz which corresponds to the ^{1}J coupling of the acetylenic proton with the ^{13}C-labeled carbon atom.

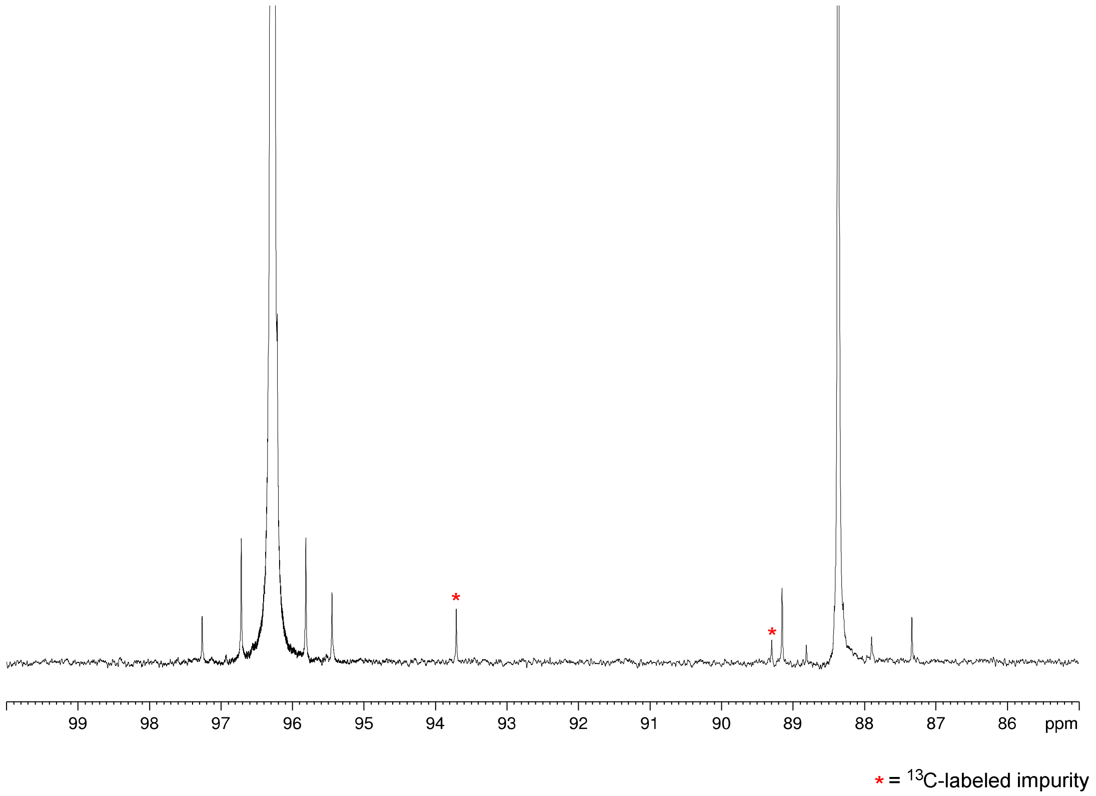

Figure S21. Part of the ^{13}C NMR spectrum of ^{13}C(1)-**12i** and ^{13}C(2)-**12i** in $CDCl_3$ at 101 MHz.

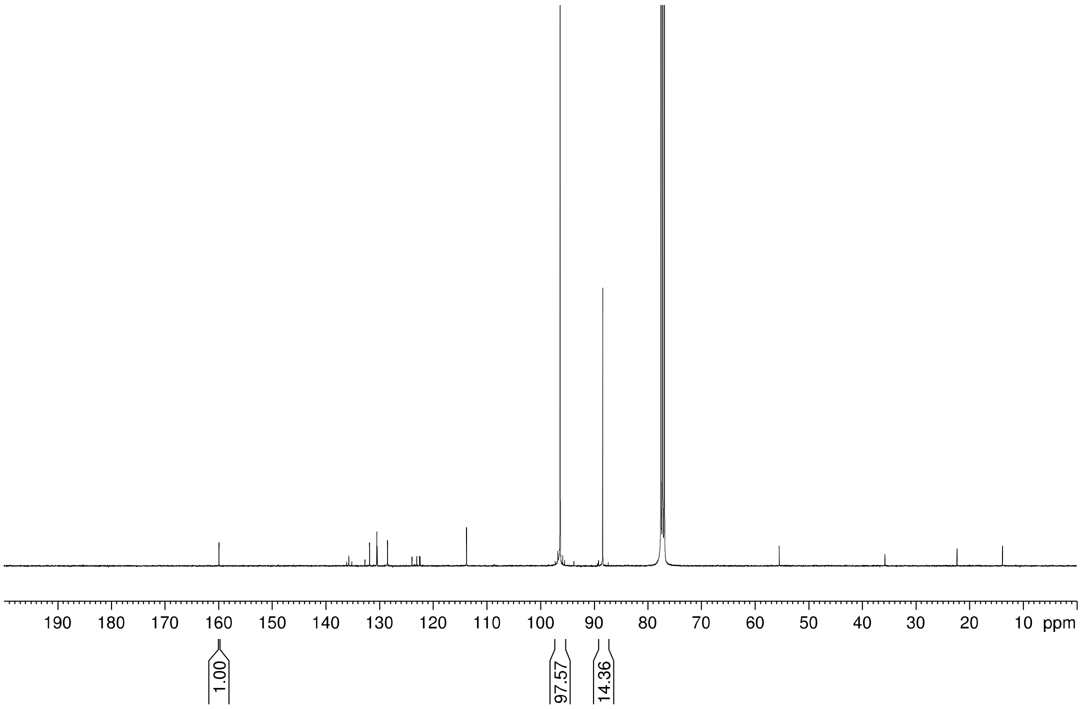

Figure S22. Quantitative ^{13}C NMR spectrum of ^{13}C(1)-**12i** and ^{13}C(2)-**12i** in $CDCl_3$ at 101 MHz measured with inverse gated-decoupling. The integrals for both acetylenic carbon atoms are referenced to the quaternary carbon atom ($C_{ar}OCH_3$) at δ = 159.9 ppm.

4. Computational Details, Cartesian Coordinates and Absolute Energies for All Calculated Compounds

Computational Details. All calculations were performed by using the program package Gaussian 16[10]. The geometrical parameters of all stationary points were optimized by means of B3LYP[11-13] with additional dispersion correction with Becke-Johnson damping[14]. For this optimization process the basis set B1 was used. For this basis set 6-31G(d)[15-16] was employed for the atoms C, H, Cl and P. For Au the def2-TZVP+ECP[17-18] basis was applied. For all stationary points no symmetry restriction was applied. Frequency calculations were carried out at each of the structures to verify the nature of the stationary point. It turned out that all transition states have exactly one imaginary frequency, whereas all other structures have none. Furthermore, the energies of the stationary points were calculated using the density functionals B3LYP, PBE0[19-20], M06-2X[21] and B97-D[22] with additional dispersion correction[23-24,14]. Two different types of basis sets were used (B2 and B3). In the case of B2 the def2-TZVP+ECP basis was used for Au, whereas for B3 the aug-cc-pVTZ-PP+ECP[25-26] basis set was employed. For C, H, Cl and P the 6-311++G(d,p)[27-30] basis was applied in both cases (B2 and B3). To determine the solvent effect, the single point calculations were also performed using dichloroethane (DCE) as solvent.

Taking a look at the data in Tables S1 and S2 shows that the usage of the smaller basis set B1 leads to overall smaller energy values compared to the larger basis sets B2 and B3. However, the differences between the values for B2 and B3 are rather small. Taking solvent effects into account and replacing Me_3P by JohnPhos as ligand results in higher energy values, although the tendencies remain the same.

Table S3. Absolute energies [au] of Me_3PAu^+, **1d**, **11a** and **14-25** ($[Au]^+ = Me_3PAu^+$) calculated by B3LYP-D3BJ/6-31G(d),def2-TZVP+ECP.

	E (B3LYP)	*G* (B3LYP)
Me_3PAu^+	-596.707922	-596.626241
**1d**	-767.997889	-767.929750
**11a**	-347.740961	-347.638084
**14**	-1364.777586	-1364.610219
**15**	-1712.539841	-1712.246311
**16**	-1712.572859	-1712.276702
**17**	-1712.570006	-1712.270734
**18**	-1712.592441	-1712.290834
**19**	-1712.575164	-1712.276178
**20**	-1712.614669	-1712.317463
**21**	-1712.526590	-1712.233259
**22**	-1712.571868	-1712.272261

**23**	-1712.561881	-1712.262690
**24**	-1712.563583	-1712.267214
**25**	-1712.555191	-1712.257833

Table S4. Absolute energies [au] of Me_3PAu^+, **1d**, **11a** and **14-25** ($[Au]^+$ = Me_3PAu^+) calculated by different methods.

	E (B3LYP)[a]	*E* (B3LYP)[b]	*E* (B3LYP)[c]	*E* (B3LYP)[d]
Me_3PAu^+	-596.773805	-596.869363	-596.767094	-596.862990
**1d**	-768.116148	-768.125992	-768.116148	-768.125992
**11a**	-347.840563	-347.853638	-347.840563	-347.853638
**14**	-1364.955261	-1365.030847	-1364.950126	-1365.025728
**15**	-1712.812536	-1712.887080	-1712.806985	-1712.881505
**16**	-1712.841217	-1712.915912	-1712.835846	-1712.910444
**17**	-1712.838010	-1712.914834	-1712.832607	-1712.909273
**18**	-1712.860351	-1712.933325	-1712.855021	-1712.927985
**19**	-1712.842250	-1712.917481	-1712.837148	-1712.912351
**20**	-1712.884691	-1712.964600	-1712.879415	-1712.959252
**21**	-1712.800203	-1712.876748	-1712.794779	-1712.871283
**22**	-1712.839475	-1712.913844	-1712.834090	-1712.908383
**23**	-1712.830285	-1712.906790	-1712.824876	-1712.901254
**24**	-1712.831990	-1712.908158	-1712.826627	-1712.902680
**25**	-1712.824209	-1712.896878	-1712.818814	-1712.891430

[a] B3LYP-D3BJ/6-311++G(d,p),def2-TZVP+ECP//B3LYP-D3BJ/6-31G(d),def2-TZVP+ECP.
[b] B3LYP-D3BJ/6-311++G(d,p),def2-TZVP+ECP(DCE as solvent)//B3LYP-D3BJ/6-31G(d),def2-TZVP+ECP.
[c] B3LYP-D3BJ/6-311++G(d,p),aug-cc-pVTZ-PP+ECP//B3LYP-D3BJ/6-31G(d),def2-TZVP+ECP.
[d] B3LYP-D3BJ/6-311++G(d,p),aug-cc-pVTZ-PP+ECP(DCE as solvent)//B3LYP-D3BJ/6-31G(d),def2-TZVP+ECP.

Table S5. Absolute energies [au] of Me_3PAu^+, **1d**, **11a** and **14-25** ($[Au]^+$ = Me_3PAu^+) calculated by different methods.

	E (PBE0)[a]	*E* (M06-2X)[b]	*E* (B97)[c]
Me_3PAu^+	-596.493406	-596.564345	-597.018541
**1d**	-767.568049	-767.937280	-767.899939
**11a**	-347.404556	-347.664294	-347.584766
**14**	-1364.100554	-1364.526079	-1364.953551
**15**	-1711.504913	-1712.184698	-1712.545821
**16**	-1711.540903	-1712.210941	-1712.573536
**17**	-1711.538644	-1712.210293	-1712.570937
**18**	-1711.566280	-1712.236648	-1712.587842
**19**	-1711.548497	-1712.225425	-1712.573538
**20**	-1711.591863	-1712.271681	-1712.616574
**21**	-1711.495687	-1712.179312	-1712.535804
**22**	-1711.538510	-1712.208765	-1712.571489
**23**	-1711.530439	-1712.201787	-1712.564401
**24**	-1711.532168	-1712.202319	-1712.565259
**25**	-1711.524684	-1712.192713	-1712.554628

[a] PBE0-D3BJ/6-311++G(d,p),aug-cc-pVTZ-PP+ECP(DCE as solvent)//B3LYP-D3BJ/6-31G(d),def2-TZVP+ECP.
[b] M06-2X-D3/6-311++G(d,p),aug-cc-pVTZ-PP+ECP(DCE as solvent)//B3LYP-D3BJ/6-31G(d),def2-TZVP+ECP.
[c] B97D3/6-311++G(d,p),aug-cc-pVTZ-PP+ECP(DCE as solvent)//B3LYP-D3BJ/6-31G(d),def2-TZVP+ECP.

Table S6. Absolute energies [au] of JohnPhosAu+ and **14-25** ([Au]+ = JohnPhosAu+) calculated by different methods.

	E (B3LYP)[a]	*G* (B3LYP)[a]	*E* (B3LYP)[b]	*E* (B3LYP)[c]
JohnPhosAu+	-1255.475093	-1255.105478	-1255.702991	-1255.787726
	-767.997889	-767.929750	-768.116148	-768.125992
**14**	-2023.536105	-2023.077866	-2023.878993	-2023.958875
**15**	-2371.294918	-2370.709765	-2371.732157	-2371.814645
**16**	-2371.325878	-2370.737279	-2371.759142	-2371.842177
**17**	-2371.320140	-2370.729739	-2371.753799	-2371.839188
**18**	-2371.346187	-2370.753897	-2371.779381	-2371.860836
**19**	-2371.327342	-2370.738474	-2371.760072	-2371.843043
**20**	-2371.374603	-2370.783796	-2371.808809	-2371.892253
**21**	-2371.286116	-2370.699121	-2371.723196	-2371.804528
**22**	-2371.324505	-2370.732896	-2371.757226	-2371.840031
**23**	-2371.311786	-2370.722349	-2371.745889	-2371.831181
**24**	-2371.312459	-2370.724661	-2371.746649	-2371.832360
**25**	-2371.305795	-2370.719016	-2371.740598	-2371.822736

[a] B3LYP-D3BJ/6-31G(d),def2-TZVP+ECP.

[b] B3LYP-D3BJ/6-311++G(d,p),aug-cc-pVTZ-PP+ECP//B3LYP-D3BJ/6-31G(d),def2-TZVP+ECP.

[c] B3LYP-D3BJ/6-311++G(d,p),aug-cc-pVTZ-PP+ECP(DCE as solvent)//B3LYP-D3BJ/6-31G(d),def2-TZVP+ECP.

Cartesian coordinates of the optimized geometries for Me_3PAu^+ at B3LYP-D3BJ/6-31G(d),def2-TZVP+ECP level of theory (number of imaginary frequencies = 0):

Au	-0.94531500	-0.00000700	0.00000300
P	1.32938100	0.00001700	-0.00004700
C	1.98190600	-1.44017700	-0.91009300
C	1.98187900	-0.06809400	1.70225400
C	1.98181000	1.50831200	-0.79213300
H	1.63974900	-2.36666600	-0.44153400
H	3.07720900	-1.40682100	-0.89002000
H	1.63838500	-1.41580500	-1.94758100
H	1.63987800	1.56573200	-1.82885000
H	3.07710200	1.47443300	-0.77300400
H	1.63799800	2.39453300	-0.25233000
H	1.63879400	-0.97910300	2.19958500
H	1.63927600	0.80057400	2.27061300
H	3.07718000	-0.06683600	1.66338800

Cartesian coordinates of the optimized geometries for **1d** at B3LYP-D3BJ/6-31G(d),def2-TZVP+ECP level of theory (number of imaginary frequencies = 0):

C	-2.63469900	-1.20884700	0.00001300
C	-1.24265400	-1.21361900	-0.00001500
C	-0.53026600	0.00019000	-0.00001200
C	-1.24288600	1.21374400	0.00002300
C	-2.63500400	1.20862000	0.00005200
C	-3.33485700	-0.00016100	0.00004800
H	-3.17508700	-2.15106200	0.00000900
H	-0.69347400	2.14954900	0.00002500
H	-3.17556300	2.15073700	0.00007700
H	-0.69288400	-2.14921200	-0.00004300
C	0.89671500	0.00019200	-0.00005000
Cl	3.75635500	-0.00005600	-0.00001100
C	2.10697500	0.00009300	-0.00005200
H	-4.42097100	-0.00033600	0.00007100

Cartesian coordinates of the optimized geometries for **11a** at B3LYP-D3BJ/6-31G(d),def2-TZVP+ECP level of theory (number of imaginary frequencies = 0):

C	-2.14367700	-1.20822200	-0.00010500
C	-0.75141000	-1.21196700	-0.00001200
C	-0.03562700	0.00032100	0.00010300
C	-0.75187700	1.21218300	0.00008000
C	-2.14423900	1.20777900	-0.00002000
C	-2.84516800	-0.00032700	-0.00010800
H	-2.68358100	-2.15098700	-0.00017600
H	-0.20301600	2.14850600	0.00013800
H	-2.68446100	2.15036300	-0.00003100
H	-0.20190100	-2.14789100	0.00000900
C	1.39227300	0.00040900	0.00010600
C	2.60409700	0.00030300	0.00013100
H	-3.93140000	-0.00063800	-0.00018200
C	4.06265700	-0.00020100	-0.00005000
H	4.46135800	-0.50195700	0.89046700
H	4.46089000	-0.52101500	-0.87974900
H	4.45994300	1.02194900	-0.01122500

Cartesian coordinates of the optimized geometries for **14** ($[Au]^+ = Me_3PAu^+$) at B3LYP-D3BJ/6-31G(d),def2-TZVP+ECP level of theory (number of imaginary frequencies = 0):

C	-1.67578600	1.01875700	0.00057400
C	-0.80862700	1.91447900	0.00018500
C	-2.65634300	-0.00231000	0.00034000
Cl	-0.37237000	3.52909300	0.00012600
C	-3.15539400	-0.49897800	1.22500000
C	-3.15643300	-0.49711100	-1.22465400
C	-4.14727600	-1.47022300	-1.21624500
C	-4.64060700	-1.95648600	-0.00032300
C	-4.14622800	-1.47208700	1.21592900
H	-4.53860600	-1.85256300	2.15320900
H	-2.76600200	-0.10714500	2.15876800
H	-2.76782000	-0.10386400	-2.15815100
H	-4.54044000	-1.84924200	-2.15378700
Au	0.64685400	0.28968800	0.00029300
H	-5.41667000	-2.71559300	-0.00056500
P	2.49895300	-1.10504200	0.00010800
C	4.06573100	-0.16262500	-0.10516500
H	4.91796100	-0.85004800	-0.10259300
H	4.08216000	0.42809300	-1.02518100
H	4.14830100	0.51649200	0.74785200
C	2.63631500	-2.13274600	1.50996200
H	1.75723600	-2.77653900	1.60159800
H	3.53587200	-2.75516300	1.46141400
H	2.69124400	-1.48801400	2.39143900
C	2.52674000	-2.27617400	-1.40803900
H	3.42933300	-2.89464000	-1.36562600
H	1.64526100	-2.92210800	-1.37069100
H	2.51416800	-1.72095300	-2.34998000

Cartesian coordinates of the optimized geometries for **15** ($[Au]^+ = Me_3PAu^+$) at B3LYP-D3BJ/6-31G(d),def2-TZVP+ECP level of theory (number of imaginary frequencies = 1):

C	0.10899500	-1.03397900	0.72399300
C	0.92447800	-0.04772400	0.84348100
Au	-1.75481100	-0.31560000	0.05723400
C	2.84818100	-0.99029000	2.13420300
C	3.30823000	-0.98167800	1.00301900
C	3.81317300	-0.88085900	-0.32096400
C	4.70600600	0.15778300	-0.65107400
C	3.38050100	-1.77367700	-1.32104800
C	5.15786200	0.29586400	-1.96064300

H	5.03146300	0.84313300	0.12409700
C	3.83201800	-1.62084500	-2.62750300
H	2.69149300	-2.57002000	-1.05986100
C	4.72005600	-0.58833400	-2.94930500
H	5.85132400	1.09285900	-2.21040800
H	3.49731000	-2.30901000	-3.39747600
H	5.07314200	-0.47758600	-3.97002800
P	-3.86081500	0.33388500	-0.67653100
C	-4.70925400	1.50733700	0.44907600
H	-5.69892000	1.76491500	0.05771300
H	-4.81968900	1.05366700	1.43791200
H	-4.11293100	2.41844200	0.54952300
C	-3.85460500	1.16062600	-2.31377100
H	-4.87352100	1.43162700	-2.60927400
H	-3.24009500	2.06401900	-2.26831900
H	-3.42886300	0.48801900	-3.06354800
C	-5.01721600	-1.07729600	-0.85747400
H	-5.13508300	-1.58306500	0.10487600
H	-5.99535600	-0.72797100	-1.20393600
H	-4.61410600	-1.79403500	-1.57830600
C	1.35109000	1.29205400	0.69204200
C	1.85596300	1.72513100	-0.55608400
C	1.29784900	2.19209300	1.78103600
C	2.29064100	3.03720000	-0.70452300
H	1.89494000	1.02577600	-1.38295900
C	1.73697200	3.49778300	1.61869900
H	0.90657200	1.84933100	2.73313000
C	2.23490200	3.91931600	0.37829500
H	2.67718400	3.37203300	-1.66158800
H	1.69410600	4.19247900	2.45124300
H	2.57825700	4.94227800	0.25762100
Cl	0.30211000	-2.71657800	1.01682100
C	2.42591000	-1.07173000	3.52687500
H	2.16699500	-0.08135700	3.91886900
H	1.55573800	-1.72716500	3.63543100
H	3.23594300	-1.47507600	4.14576500

Cartesian coordinates of the optimized geometries for **16** ([Au]$^+$ = Me_3PAu^+) at B3LYP-D3BJ/6-31G(d),def2-TZVP+ECP level of theory (number of imaginary frequencies = 0):

C	0.34739900	-0.64974500	0.94151100
C	1.17098200	0.42917100	1.08581300
Au	-1.55965800	-0.54326900	0.16915600
C	2.48766400	0.34626800	1.86325800
C	3.27850100	-0.04456500	0.93351500
C	3.99447000	-0.45755300	-0.17483300
C	4.57826400	0.50876900	-1.04166700
C	4.13824300	-1.84629400	-0.45167400
C	5.28566300	0.08701000	-2.15429900
H	4.45208900	1.56236300	-0.81725400
C	4.85129400	-2.24691100	-1.56894100
H	3.67980700	-2.56488900	0.21823700
C	5.42267400	-1.28468900	-2.41421200
H	5.73503100	0.81354600	-2.82290100
H	4.96956800	-3.30229300	-1.79025000
H	5.98323300	-1.60763400	-3.28639400
P	-3.72268400	-0.36257400	-0.68788800
C	-4.93774200	0.30688400	0.51451000
H	-5.92990600	0.38732600	0.05837700
H	-4.99355600	-0.35236800	1.38526600
H	-4.61362600	1.29579200	0.85074000
C	-3.83650400	0.76346900	-2.13390100
H	-4.87132100	0.84775300	-2.48153000
H	-3.46607700	1.75389800	-1.85447900
H	-3.21410900	0.37925500	-2.94705400

C	-4.45993300	-1.94135100	-1.26430800
H	-4.50641800	-2.64885300	-0.43166600
H	-5.46925500	-1.77786400	-1.65595600
H	-3.83401200	-2.37440200	-2.04961100
C	0.85079400	1.73501900	0.45807100
C	0.66755000	1.81608700	-0.93305000
C	0.73806000	2.90266100	1.22799100
C	0.36397900	3.03542300	-1.53628400
H	0.77333400	0.91739300	-1.53293600
C	0.43309800	4.12098900	0.62265500
H	0.85927900	2.85342100	2.30548200
C	0.24546800	4.19036100	-0.75942000
H	0.22641700	3.08556900	-2.61243300
H	0.33697000	5.01521100	1.23082500
H	0.01058100	5.14076300	-1.22889600
Cl	0.90352100	-2.21604200	1.53658900
C	2.64139500	0.63932200	3.32280500
H	2.40226600	1.68878200	3.52157400
H	1.93637700	0.01940300	3.88576100
H	3.65879300	0.43545600	3.66284800

Cartesian coordinates of the optimized geometries for **17** ([Au]$^+$ = Me_3PAu^+) at B3LYP-D3BJ/6-31G(d),def2-TZVP+ECP level of theory (number of imaginary frequencies = 1):

C	-0.45319200	-0.33430400	-0.59406700
C	-1.02778800	0.89000600	-0.56518800
Au	1.52542300	-0.69635000	-0.12704300
C	-2.45199700	1.20129400	-0.94519600
C	-3.41110200	0.50200200	-0.43818600
C	-4.44693400	-0.21629500	0.10272300
C	-5.07552400	0.23684600	1.30307000
C	-4.91890600	-1.39923800	-0.54492500
C	-6.14172900	-0.47035300	1.82638200
H	-4.70385400	1.13593700	1.78254900
C	-5.99297100	-2.08592600	-0.01214200
H	-4.41270900	-1.73910900	-1.44082700
C	-6.59728400	-1.62410400	1.16868000
H	-6.62725700	-0.13751000	2.73732600
H	-6.36411300	-2.98303600	-0.49571000
H	-7.43548700	-2.17502500	1.58530100
P	3.75804000	-1.02545400	0.45112200
C	4.87735800	-1.31675000	-0.97553200
H	5.91205200	-1.44022000	-0.63907300
H	4.56215300	-2.21696500	-1.51057400
H	4.82111100	-0.46949000	-1.66477600
C	4.48104400	0.42485100	1.31534000
H	5.53849200	0.25770200	1.54487900
H	4.38136700	1.31228200	0.68388300
H	3.93352900	0.60507800	2.24481700
C	4.07319400	-2.44135500	1.57655200
H	3.74457200	-3.36830000	1.09820000
H	5.13857800	-2.51726100	1.81776300
H	3.50255100	-2.30953600	2.50012200
C	-0.23444600	2.08968500	-0.16890900
C	-0.60497600	2.84272800	0.95399600
C	0.88630000	2.48477200	-0.91583500
C	0.14609000	3.95172800	1.34170900
H	-1.47453600	2.54465700	1.53392300
C	1.63965200	3.59150900	-0.52367400
H	1.15508200	1.92319600	-1.80476300
C	1.27302300	4.32555100	0.60621700
H	-0.14449900	4.52069100	2.21974600
H	2.50431300	3.88873100	-1.10997900
H	1.85650200	5.19030500	0.90720800
Cl	-1.42923600	-1.74359400	-1.06216000

C	-2.71738200	2.36050200	-1.89192000
H	-2.30833300	3.27517500	-1.45466400
H	-2.19368300	2.16356500	-2.83285500
H	-3.78239800	2.49478500	-2.09080200

Cartesian coordinates of the optimized geometries for **18** ([Au]$^+$ = Me_3PAu^+) at B3LYP-D3BJ/6-31G(d),def2-TZVP+ECP level of theory (number of imaginary frequencies = 0):

C	-0.33424600	-0.07953300	0.00958100
C	-0.99346200	1.10331200	0.05201000
Au	1.61015400	-0.69214400	-0.03797600
C	-2.45548100	1.09553600	0.07058400
C	-3.03212000	-0.12668200	0.03618400
C	-4.39097800	-0.64486700	0.01290100
C	-4.77977000	-1.71536200	0.83775200
C	-5.33570200	-0.05052900	-0.84326900
C	-6.09257700	-2.17436500	0.81160800
H	-4.05811000	-2.17232200	1.50869100
C	-6.65138400	-0.50685000	-0.85195500
H	-5.03038200	0.75233400	-1.50579400
C	-7.03108600	-1.56852600	-0.02847400
H	-6.38708200	-2.99895300	1.45280500
H	-7.37602800	-0.04290900	-1.51346700
H	-8.05544400	-1.92714600	-0.04382600
P	3.87686600	-1.23075100	-0.11445400
C	4.60068800	-1.12857000	-1.79711300
H	5.66849600	-1.36917900	-1.77182500
H	4.08854500	-1.83008000	-2.46154600
H	4.46786800	-0.11776500	-2.19287200
C	4.90824400	-0.11120600	0.90990600
H	5.96617400	-0.38164400	0.83054800
H	4.77320700	0.92081900	0.57405400
H	4.59675600	-0.17864200	1.95605800
C	4.28143300	-2.92059200	0.47402900
H	3.76357500	-3.65985700	-0.14342000
H	5.36044800	-3.09771600	0.41839000
H	3.94984600	-3.03973200	1.50928700
C	-0.18824100	2.35078500	0.10778400
C	0.70045900	2.56850900	1.17120300
C	-0.29284300	3.30957800	-0.91033400
C	1.47367500	3.72889800	1.21319800
H	0.76426600	1.83560500	1.96947300
C	0.48037900	4.46785100	-0.86402000
H	-0.96493800	3.13779800	-1.74588600
C	1.36473200	4.67923800	0.19676300
H	2.15163900	3.89478800	2.04497800
H	0.39597100	5.20359600	-1.65783600
H	1.96301900	5.58450000	0.23203800
Cl	-1.69888500	-1.36957900	-0.01864200
C	-3.25686400	2.36174000	0.20298100
H	-2.78689400	3.02264400	0.93727700
H	-3.29299800	2.90691600	-0.74650300
H	-4.28118000	2.15027300	0.51529400

Cartesian coordinates of the optimized geometries for **19** ([Au]$^+$ = Me_3PAu^+) at B3LYP-D3BJ/6-31G(d),def2-TZVP+ECP level of theory (number of imaginary frequencies = 1):

C	-0.20011100	0.58999100	-0.15154500
C	1.05617000	0.61719100	-0.31763100
Au	-2.06839600	-0.21206800	-0.06862100
C	2.46340200	0.43501300	-0.45263400
C	3.01795400	-0.72157000	0.00728900
C	4.44879200	-1.05618100	0.00479200
C	4.89370300	-2.31503300	-0.43404600

C	5.38931400	-0.11899300	0.46740000
C	6.25224100	-2.61495000	-0.43836500
H	4.17286600	-3.04847600	-0.77928800
C	6.74686300	-0.42950100	0.47242700
H	5.05062200	0.83759600	0.85128700
C	7.18148900	-1.67436200	0.01366200
H	6.58704500	-3.58531400	-0.79115900
H	7.46336800	0.29643200	0.84387500
H	8.24012800	-1.91481000	0.01651200
P	-4.16791400	-1.21098300	0.03510300
C	-4.30004200	-2.48753900	1.34566200
H	-5.30095200	-2.93133200	1.34915500
H	-3.55852100	-3.27209000	1.17104700
H	-4.10251800	-2.03556300	2.32164800
C	-5.53913500	-0.04008400	0.37336400
H	-6.49548200	-0.57171600	0.41305400
H	-5.36687800	0.46292300	1.32896200
H	-5.58054900	0.71687500	-0.41477100
C	-4.64879200	-2.07025300	-1.51250200
H	-3.91461700	-2.84644400	-1.74569800
H	-5.63711500	-2.52898800	-1.40495100
H	-4.67137300	-1.35523500	-2.33960300
C	0.28272100	2.14214900	-0.00111500
C	0.10820200	3.00999900	-1.09986400
C	0.55720900	2.66753000	1.27970000
C	0.18496200	4.38414600	-0.91184700
H	-0.09155700	2.58769100	-2.07928300
C	0.64485300	4.04236600	1.45529900
H	0.70111000	1.98401100	2.11008700
C	0.45507500	4.89757500	0.36241800
H	0.04412400	5.05722600	-1.75125300
H	0.85828700	4.45300000	2.43682200
H	0.52278700	5.97187300	0.50434500
Cl	1.96245500	-1.97173200	0.65004600
C	3.23382800	1.52922100	-1.15794400
H	2.69998000	1.83844600	-2.06192200
H	3.33894800	2.41450800	-0.51966000
H	4.22837500	1.18666000	-1.44260400

Cartesian coordinates of the optimized geometries for **20** ([Au]$^+$ = Me_3PAu^+) at B3LYP-D3BJ/6-31G(d),def2-TZVP+ECP level of theory (number of imaginary frequencies = 0):

C	-0.91501100	1.69152400	-0.22196300
C	0.25621700	1.31237500	-0.36810500
Au	-0.97425500	-0.61706100	-0.20421500
C	1.64728800	1.16020500	-0.65940900
C	2.50527200	0.67557600	0.27763000
C	3.96617800	0.57959400	0.15558900
C	4.64955600	-0.58139300	0.55666800
C	4.70082200	1.66519000	-0.35253400
C	6.03127600	-0.66455200	0.41948700
H	4.09273400	-1.41649900	0.96857400
C	6.08553400	1.58049200	-0.47746500
H	4.18823700	2.58362400	-0.61775700
C	6.75263400	0.41457500	-0.09848500
H	6.54822800	-1.56992500	0.72180100
H	6.64332800	2.42959500	-0.85969800
H	7.83190700	0.34988100	-0.19575000
P	-1.64357300	-2.81716700	-0.07341400
C	-0.26150700	-3.93305500	0.37574900
H	-0.61803000	-4.96488800	0.45980300
H	0.51757400	-3.88164100	-0.38981100
H	0.16822600	-3.61875900	1.33084300
C	-2.93285900	-3.09538300	1.19813400
H	-3.20430500	-4.15546300	1.23680300

H	-2.55787300	-2.78269900	2.17655500
H	-3.82093500	-2.50366000	0.95982800
C	-2.33462200	-3.48607700	-1.63216400
H	-1.58803200	-3.41367200	-2.42779500
H	-2.62242600	-4.53433500	-1.50089700
H	-3.21311800	-2.90452500	-1.92491900
C	-2.17968200	2.31659400	0.02024900
C	-3.02917800	2.63561600	-1.05621400
C	-2.56817700	2.61682800	1.34055600
C	-4.24793000	3.25886900	-0.80926700
H	-2.72076200	2.40048400	-2.06970700
C	-3.79057600	3.23836800	1.57258500
H	-1.90361000	2.36982700	2.16201000
C	-4.62966400	3.55948800	0.50147900
H	-4.90052500	3.51276300	-1.63841500
H	-4.08846000	3.47728700	2.58848000
H	-5.58155300	4.04668500	0.68837200
Cl	1.84996300	0.10241300	1.81265300
C	2.05435400	1.54177400	-2.06862800
H	1.33705000	1.12730300	-2.78492800
H	2.05464300	2.63000700	-2.19890800
H	3.04849600	1.16325100	-2.30880200

Cartesian coordinates of the optimized geometries for **21** ($[Au]^+ = Me_3PAu^+$) at B3LYP-D3BJ/6-31G(d),def2-TZVP+ECP level of theory (number of imaginary frequencies = 1):

C	0.44030900	-0.22052100	-0.59551900
C	0.72877700	-1.45289900	-0.49972400
C	1.07984600	1.03623400	-0.97831800
Cl	0.42035000	-3.03937800	-0.15070600
C	1.01709600	2.14569500	-0.12041100
C	1.77834600	1.13296300	-2.19144500
C	2.42583900	2.32001200	-2.52636900
C	2.38236200	3.41480300	-1.65897700
C	1.67612600	3.32489800	-0.45716300
H	1.63913900	4.17338800	0.21882400
H	0.48233200	2.06458800	0.82076200
H	1.81127700	0.28085500	-2.85988600
H	2.96582600	2.38997700	-3.46553400
Au	-1.63571800	-0.09904600	-0.04934000
C	3.01689000	-1.92063800	-1.48083000
C	3.30693600	-1.34374400	-0.44433500
C	3.63214500	-0.62973500	0.74383700
C	3.55797000	-1.26481900	1.99806200
C	4.01322800	0.72329800	0.66764700
C	3.85594800	-0.55252700	3.15530300
H	3.27056000	-2.31011500	2.04992500
C	4.31102000	1.42503300	1.83104700
H	4.06186700	1.20929700	-0.30017300
C	4.23198900	0.79147700	3.07402200
H	3.80131600	-1.04517000	4.12110700
H	4.60479800	2.46820500	1.76835100
H	4.46780300	1.34271300	3.97931100
H	2.89103900	4.33669400	-1.92312800
P	-3.85803000	0.28119100	0.47349600
C	-5.03025700	-0.55125700	-0.66335600
H	-6.06268700	-0.32464300	-0.37738400
H	-4.85603400	-0.20916600	-1.68723300
H	-4.87521100	-1.63304200	-0.62677500
C	-4.32828100	-0.27744400	2.15470800
H	-5.38399900	-0.05908200	2.34654700
H	-4.16047900	-1.35405600	2.24642800
H	-3.71272000	0.23510200	2.89908200
C	-4.30043100	2.05858900	0.41027000
H	-4.10721500	2.45136100	-0.59178400

H	-5.35893900	2.19600700	0.65432600
H	-3.69049100	2.61661700	1.12603700
C	2.83476900	-2.65643500	-2.72749900
H	2.16417300	-3.51201900	-2.58886100
H	2.41332400	-2.01814000	-3.51243500
H	3.79675800	-3.04105900	-3.08575600

Cartesian coordinates of the optimized geometries for **22** ($[Au]^+ = Me_3PAu^+$) at B3LYP-D3BJ/6-31G(d),def2-TZVP+ECP level of theory (number of imaginary frequencies = 0):

C	0.30009100	-0.25118900	-0.67503500
C	0.84175900	-1.48674300	-0.84849400
C	1.07219800	0.96392800	-1.00691800
Cl	-0.04673400	-2.95823600	-0.44339200
C	1.08837100	2.03921000	-0.09820300
C	1.78360500	1.10151500	-2.21354500
C	2.49814600	2.26510200	-2.49175200
C	2.53108300	3.31180600	-1.56468200
C	1.82277300	3.19330800	-0.36713900
H	1.82825000	4.00654300	0.35255300
H	0.52524600	1.95436300	0.82641300
H	1.74620500	0.30535600	-2.94993100
H	3.02304000	2.35983900	-3.43774000
Au	-1.65677200	0.00308200	-0.03282200
C	2.24302900	-1.81553400	-1.28221900
C	2.96811600	-1.36230100	-0.31637700
C	3.64344200	-0.73720200	0.70553300
C	3.94123600	-1.43823300	1.91197200
C	4.03699400	0.62609100	0.55130000
C	4.60453800	-0.78378900	2.93202400
H	3.63410400	-2.47384600	2.00953600
C	4.71026200	1.25698300	1.58382900
H	3.81326400	1.14080800	-0.37462400
C	4.98822800	0.55812400	2.76594000
H	4.83403100	-1.30262400	3.85652200
H	5.01772900	2.29169400	1.47761200
H	5.51459200	1.06210100	3.57121400
H	3.08669900	4.21804900	-1.78523200
P	-3.87619400	0.31178700	0.63547900
C	-5.09726800	-0.39972800	-0.53710500
H	-6.11932600	-0.23524900	-0.18028100
H	-4.97857000	0.06742500	-1.51880000
H	-4.92041000	-1.47374200	-0.64320500
C	-4.29182000	-0.45634900	2.25094700
H	-5.34516800	-0.29088600	2.50000700
H	-4.09665000	-1.53149700	2.20557100
H	-3.66421900	-0.02494500	3.03587400
C	-4.38214000	2.06780500	0.81612700
H	-4.24318700	2.58856800	-0.13550000
H	-5.43233800	2.14069700	1.11726100
H	-3.75713200	2.55486800	1.56996900
C	2.59198200	-2.59095400	-2.51759900
H	2.10193800	-3.56903100	-2.48254600
H	2.21571900	-2.05959700	-3.39807100
H	3.67083400	-2.73024400	-2.60978300

Cartesian coordinates of the optimized geometries for **23** ($[Au]^+ = Me_3PAu^+$) at B3LYP-D3BJ/6-31G(d),def2-TZVP+ECP level of theory (number of imaginary frequencies = 1):

C	0.01121700	0.68481000	-0.21624100
C	1.14666900	0.03107300	-0.52088800
C	0.01380800	2.13766500	0.02614500
Cl	1.21380000	-1.74469200	-0.55497700
C	0.89140100	2.70713500	0.96792800

C	-0.87869500	2.98159700	-0.66065700
C	-0.85255700	4.35742100	-0.45376900
C	0.03677600	4.91356400	0.47110000
C	0.89965600	4.08247400	1.18998200
H	1.57679100	4.50751100	1.92518100
H	1.55713500	2.05811100	1.52910600
H	-1.57368000	2.54678000	-1.37317000
H	-1.53115900	4.99898400	-1.00781100
Au	-1.81031400	-0.29762800	-0.01035700
C	2.45274500	0.67384100	-0.90607200
C	3.53012600	0.11627700	-0.45198500
C	4.63283800	-0.58557100	-0.04305700
C	5.11128800	-1.68066800	-0.82898100
C	5.31064400	-0.22719900	1.16328300
C	6.23129300	-2.37790400	-0.41832400
H	4.57393700	-1.94840600	-1.73191700
C	6.42592900	-0.94077800	1.55888300
H	4.93411400	0.60447200	1.74874800
C	6.88335100	-2.00865900	0.76960200
H	6.60432200	-3.21042800	-1.00505700
H	6.94864900	-0.67960300	2.47264600
H	7.76121100	-2.56384600	1.08687700
H	0.04632600	5.98586500	0.64043900
P	-3.88190500	-1.34299200	0.25507500
C	-4.57101400	-2.05674800	-1.29053800
H	-5.53646200	-2.53710100	-1.10006700
H	-4.70273300	-1.26502100	-2.03342400
H	-3.87362300	-2.79575500	-1.69497500
C	-3.85649100	-2.74939500	1.43582700
H	-4.84905900	-3.20400400	1.52076600
H	-3.14234000	-3.50285400	1.09184000
H	-3.53649700	-2.39551800	2.41992300
C	-5.20931400	-0.24062800	0.88348100
H	-5.35584600	0.59024900	0.18761000
H	-6.15064800	-0.78898400	0.99408600
H	-4.91517400	0.17076200	1.85316400
C	2.50112000	1.88437800	-1.82906600
H	1.74798100	1.75117900	-2.60956200
H	2.27293100	2.79897200	-1.28012500
H	3.48787900	1.97981600	-2.28750500

Cartesian coordinates of the optimized geometries for **24** ([Au]$^+$ = Me_3PAu^+) at B3LYP-D3BJ/6-31G(d),def2-TZVP+ECP level of theory (number of imaginary frequencies = 0):

C	-0.05623800	0.77907500	0.02232800
C	1.18142000	0.26214000	0.02147700
C	-0.30158400	2.23938300	-0.00112600
Cl	1.41215400	-1.51448200	-0.08119000
C	-0.74851500	2.90970500	1.14899300
C	-0.13800500	2.96449400	-1.19254600
C	-0.38258200	4.33696500	-1.22228800
C	-0.79259800	5.00351500	-0.06545500
C	-0.97711900	4.28443600	1.11812700
H	-1.30604100	4.79460000	2.01869400
H	-0.90149600	2.34848600	2.06648700
H	0.18065800	2.44382200	-2.09130200
H	-0.25202000	4.88656500	-2.14996400
Au	-1.76976500	-0.39180100	-0.00092900
C	2.49886900	0.91293000	0.12919400
C	3.56286600	0.15716600	0.06494000
C	4.75778900	-0.51587300	0.02919100
C	5.44276200	-0.69567300	-1.21217300
C	5.32499800	-1.03602200	1.23360200
C	6.65314600	-1.36286700	-1.23636000
H	4.99484700	-0.30268400	-2.11829600

C	6.53738400	-1.69803600	1.18933600
H	4.78683300	-0.90117800	2.16542700
C	7.19581200	-1.85980100	-0.04052400
H	7.18298400	-1.50426800	-2.17217500
H	6.97931300	-2.09462900	2.09702900
H	8.14688700	-2.38356400	-0.06753600
H	-0.97900100	6.07269500	-0.08880700
P	-3.75731200	-1.62188400	-0.03049900
C	-3.92166400	-2.74184800	-1.47677800
H	-4.86761500	-3.29199200	-1.43711100
H	-3.88371300	-2.15618300	-2.39962000
H	-3.09061500	-3.45272500	-1.48565200
C	-3.98645200	-2.71867800	1.42472000
H	-4.93023800	-3.26914900	1.35195800
H	-3.15731600	-3.42960000	1.48131200
H	-3.98881200	-2.11883900	2.33920000
C	-5.27690600	-0.59167000	-0.07297600
H	-5.26450300	0.04203600	-0.96422800
H	-6.17337800	-1.22025000	-0.08883400
H	-5.30547300	0.05542100	0.80823000
C	2.71350900	2.40329800	0.36584700
H	2.38523400	2.97572500	-0.50283800
H	2.11876500	2.72269700	1.22413200
H	3.76785000	2.61392100	0.55979300

Cartesian coordinates of the optimized geometries for **25** ([Au]$^+$ = Me_3PAu^+) at B3LYP-D3BJ/6-31G(d),def2-TZVP+ECP level of theory (number of imaginary frequencies = 1):

C	-0.13645700	0.88617200	0.01739100
C	1.12623600	0.50156800	0.00494700
C	-0.37414000	2.35674900	0.02909800
Cl	1.62070900	-1.37836100	-0.11216900
C	-1.07649000	2.94839800	1.09122300
C	0.07504800	3.16076300	-1.03012800
C	-0.16230100	4.53508700	-1.01925900
C	-0.83790000	5.12147900	0.05197000
C	-1.29321300	4.32446500	1.10593300
H	-1.82589000	4.77510500	1.93791100
H	-1.43829300	2.32641600	1.90467000
H	0.58689300	2.69909800	-1.86947700
H	0.17966200	5.14656200	-1.84892800
Au	-1.77904700	-0.37911300	-0.01762200
C	2.50835700	0.92136500	0.13110600
C	3.25888900	-0.18346500	0.08742400
C	4.56610400	-0.74709900	0.05249300
C	5.39147900	-0.49808600	-1.06600300
C	5.05140000	-1.52147300	1.12836100
C	6.69179500	-0.98984200	-1.08659400
H	5.00277300	0.08349600	-1.89532200
C	6.34552700	-2.02196900	1.08799900
H	4.40724400	-1.71420200	1.98022700
C	7.16503000	-1.75328600	-0.01538700
H	7.33319300	-0.78860400	-1.93837300
H	6.72346400	-2.61516700	1.91421000
H	8.17760500	-2.14419700	-0.03988300
H	-1.01908100	6.19172600	0.06158700
P	-3.68415600	-1.72800200	-0.07330800
C	-3.71506800	-2.92085700	-1.46811000
H	-4.63455600	-3.51499800	-1.44743900
H	-3.65788700	-2.37855900	-2.41608300
H	-2.85259100	-3.58980600	-1.40003300
C	-3.92214000	-2.75888400	1.42672000
H	-4.83296500	-3.36051400	1.34117500
H	-3.06291900	-3.42270500	1.55735000
H	-3.99770400	-2.11446000	2.30708000

C	-5.24581800	-0.77788600	-0.23872300
H	-5.22150800	-0.18922300	-1.16006400
H	-6.10791900	-1.45245900	-0.26377600
H	-5.34972600	-0.09149500	0.60630300
C	2.99940900	2.32064000	0.35039600
H	2.82965000	2.93543300	-0.53765900
H	2.45387400	2.78384800	1.17807000
H	4.06722000	2.31375600	0.58195400

Cartesian coordinates of the optimized geometries for JohnPhosAu$^+$ at B3LYP-D3BJ/6-31G(d),def2-TZVP+ECP level of theory (number of imaginary frequencies = 0):

Au	0.96134700	-1.21126000	-0.24412600
P	-1.11203000	-0.26326400	0.12328000
C	-1.63324400	-0.45033700	1.92877200
C	-2.31284100	-0.98896600	-1.15996500
C	-0.44074400	0.03197200	2.77635400
H	0.45764700	-0.56897500	2.60268000
H	-0.70641400	-0.05525100	3.83557400
H	-0.20231600	1.08060000	2.57660300
C	-2.86255200	0.40816900	2.27852100
H	-3.09485500	0.25358600	3.33816100
H	-3.75013000	0.13341300	1.70602800
H	-2.66679500	1.47362300	2.13613100
C	-1.90919500	-1.93467200	2.21919800
H	-1.06153700	-2.56941700	1.93951700
H	-2.80276800	-2.29869200	1.70532800
H	-2.07718600	-2.05673400	3.29484900
C	-2.02996200	-2.50187200	-1.27406700
H	-1.01246400	-2.69676600	-1.62719600
H	-2.72687800	-2.93105200	-2.00232100
H	-2.16956300	-3.02936500	-0.32795100
C	-3.80195400	-0.80065200	-0.81602000
H	-4.08304900	-1.32554500	0.09997600
H	-4.39401000	-1.23023000	-1.63175400
H	-4.09791100	0.24594400	-0.72422800
C	-1.98643300	-0.31941400	-2.50825700
H	-0.92446300	-0.41643800	-2.76141500
H	-2.24668200	0.74109800	-2.52182500
H	-2.56021100	-0.82180500	-3.29461600
C	-0.91181500	1.54774800	-0.18415300
C	-2.09237300	2.30168300	-0.31889700
C	0.33527000	2.22844000	-0.24516400
C	-2.06510200	3.68020600	-0.51072500
H	-3.05484200	1.81074000	-0.26612000
C	0.33934800	3.61821100	-0.42881700
C	-0.84151000	4.34374100	-0.56509500
H	-2.99646300	4.22781500	-0.61263400
H	1.29614700	4.12911300	-0.47103300
H	-0.80356200	5.41837900	-0.71213300
C	1.66298500	1.56040700	-0.09341800
C	2.22909300	0.82179500	-1.15738000
C	2.34703200	1.61372300	1.12478200
C	3.39761100	0.06200500	-0.95324500
H	1.77017200	0.86372700	-2.14117500
C	3.52415100	0.88083600	1.32171300
H	1.93720000	2.20591900	1.93678400
C	4.03508400	0.08177000	0.30039600
H	3.83627200	-0.48457100	-1.78278100
H	4.03232500	0.92825200	2.27954100
H	4.93931000	-0.49764800	0.45557500

Cartesian coordinates of the optimized geometries for **14** ([Au]$^+$ = JohnPhosAu$^+$) at B3LYP-D3BJ/6-31G(d),def2-TZVP+ECP level of theory (number of imaginary frequencies = 0):

C	2.87308600	0.25667500	0.37740100
C	2.51915900	1.13196800	1.18526800
C	3.30327600	-0.78360900	-0.49623600
Cl	2.76091800	2.42751500	2.20365300
C	3.91910400	-1.93229200	0.03960500
C	3.08825200	-0.67692100	-1.88504300
C	3.49150600	-1.70945900	-2.72334400
C	4.10684300	-2.84660500	-2.18952400
C	4.32152700	-2.95536600	-0.81227900
H	4.80178300	-3.83862500	-0.40387500
H	4.07496800	-2.00523300	1.11073600
H	2.60273100	0.20917700	-2.27760700
H	3.32987800	-1.63036500	-3.79362800
Au	0.55962900	0.22191200	0.70774200
H	4.42054700	-3.64983100	-2.84900000
P	-1.54024700	-0.76166200	0.45024400
C	-2.42392100	-0.69082700	2.12326700
C	-1.20923400	-2.51489100	-0.22043600
C	-2.38626600	0.79149900	2.54770600
H	-1.36103700	1.15543900	2.67473600
H	-2.90327700	0.89904800	3.50786100
H	-2.89617800	1.43190000	1.82077000
C	-3.89185100	-1.14855700	2.09746800
H	-4.27914500	-1.11234900	3.12211800
H	-4.01281100	-2.17316100	1.73938700
H	-4.51260900	-0.48602400	1.49062000
C	-1.63365700	-1.53603100	3.13762100
H	-0.57080000	-1.27194700	3.15451400
H	-1.72385800	-2.60719500	2.93731400
H	-2.03874900	-1.35430600	4.13936100
C	0.02640800	-3.10717700	0.48827800
H	0.92795800	-2.51927100	0.29430000
H	0.19851100	-4.11612200	0.09631800
H	-0.10373400	-3.18950100	1.56930200
C	-2.38582000	-3.48972100	-0.05249100
H	-2.62178400	-3.67349000	0.99894200
H	-2.10363800	-4.45178500	-0.49542600
H	-3.28941500	-3.15536000	-0.56523200
C	-0.87600300	-2.34149800	-1.71549000
H	-0.05133000	-1.63517700	-1.86586100
H	-1.73705800	-1.99734400	-2.29326900
H	-0.55917100	-3.31025900	-2.11812700
C	-2.61516000	0.07613600	-0.78017500
C	-3.86617900	-0.50421000	-1.06895900
C	-2.21321100	1.21760500	-1.51422900
C	-4.70378100	0.01213000	-2.05214400
H	-4.19579900	-1.37672500	-0.52316200
C	-3.06947600	1.71749700	-2.50881200
C	-4.30057700	1.12943100	-2.78144100
H	-5.66161900	-0.45974800	-2.24676700
H	-2.75340200	2.59423100	-3.06519200
H	-4.94032500	1.54331900	-3.55457900
C	-0.93427900	1.96402200	-1.32630400
C	0.07784300	1.87187600	-2.29275500
C	-0.77224400	2.86062100	-0.25795600
C	1.23594300	2.64474400	-2.18270000
H	-0.05343100	1.19730800	-3.13393300
C	0.38391800	3.63693200	-0.15276300
H	-1.56614200	2.96547100	0.47455100
C	1.39333700	3.52618100	-1.11145500
H	2.00668500	2.57444100	-2.94508800
H	0.48968500	4.33393900	0.67250800
H	2.28874600	4.13475300	-1.03245400

Cartesian coordinates of the optimized geometries for **15** ([Au]$^+$ = JohnPhosAu$^+$) at B3LYP-D3BJ/6-31G(d),def2-TZVP+ECP level of theory (number of imaginary frequencies = 1):

C	-1.54161300	0.99140500	-0.97650100
C	-2.15504500	0.79420200	0.13678600
Au	0.34091300	0.08687900	-0.69314900
P	2.29386300	-1.11306700	-0.24805200
C	1.89498200	-2.22821800	1.23784000
C	2.80655200	-2.04470800	-1.82336500
C	1.36564100	-1.28443000	2.33735500
H	0.44769500	-0.77408500	2.03454300
H	1.14093000	-1.87499700	3.23315700
H	2.10848700	-0.52827400	2.61112000
C	3.09790000	-3.00141300	1.80185600
H	2.74625400	-3.63216200	2.62683200
H	3.56590200	-3.65747500	1.06547000
H	3.85784900	-2.32871000	2.20543100
C	0.78229700	-3.21138900	0.83500100
H	-0.07765400	-2.69423000	0.39666200
H	1.13551300	-3.96537500	0.12587700
H	0.43435300	-3.73867900	1.73091300
C	1.52341900	-2.49563200	-2.55276300
H	0.89641000	-1.64319300	-2.83134200
H	1.80835500	-3.02109200	-3.47161800
H	0.91833900	-3.17903400	-1.95204300
C	3.69722100	-3.27871200	-1.60569700
H	3.18820300	-4.05640000	-1.03035600
H	3.94113900	-3.70607200	-2.58531600
H	4.64430000	-3.04397100	-1.11612000
C	3.53673900	-1.01525000	-2.70770300
H	2.92809900	-0.11703000	-2.86359300
H	4.49536900	-0.71049100	-2.28079800
H	3.72625100	-1.46244100	-3.69013600
C	3.72057200	-0.08690800	0.29111400
C	4.96092800	-0.71875300	0.50337400
C	3.61431300	1.29867700	0.55627000
C	6.06779100	-0.02177700	0.97686000
H	5.06699200	-1.77625700	0.30475600
C	4.73743900	1.98133000	1.05152900
C	5.95267000	1.33762400	1.26299400
H	7.00962300	-0.54062300	1.12515700
H	4.64488300	3.04375900	1.25400600
H	6.80416600	1.89559200	1.64001700
C	2.39961200	2.13596800	0.32182300
C	2.10802300	2.61193400	-0.96714100
C	1.61255300	2.56759700	1.39766400
C	1.04807000	3.49586500	-1.17505000
H	2.73164400	2.30222200	-1.80036700
C	0.55112400	3.45064500	1.18775500
H	1.84171200	2.21573300	2.39909800
C	0.26804900	3.91742000	-0.09643800
H	0.83785500	3.85878900	-2.17606100
H	-0.03706600	3.79504000	2.03409800
H	-0.54331500	4.62090100	-0.25587800
C	-4.06643100	2.21578400	0.13007000
C	-4.60112500	1.15272500	-0.15039900
C	-5.15293300	-0.12998200	-0.40952800
C	-4.99340500	-0.73147700	-1.67338500
C	-5.80965400	-0.83046400	0.62125700
C	-5.48107000	-2.01485900	-1.89420500
H	-4.48184600	-0.18662300	-2.45977800
C	-6.30012800	-2.11174400	0.38588600
H	-5.92328100	-0.36149600	1.59268300
C	-6.13443100	-2.70560600	-0.86762800
H	-5.35676600	-2.47858100	-2.86779900

H	-6.81116200	-2.64762800	1.17966400
H	-6.51715700	-3.70559700	-1.04766000
Cl	-2.00245600	1.80477200	-2.41236000
C	-2.23576500	0.17051900	1.41730800
C	-1.87983800	0.87833600	2.58313300
C	-2.64225400	-1.17735300	1.51106200
C	-1.92159700	0.24403900	3.81841300
H	-1.54838500	1.90645500	2.49369600
C	-2.67635700	-1.80309300	2.75300900
H	-2.91024900	-1.71302100	0.60786800
C	-2.31968400	-1.09528800	3.90426700
H	-1.63956100	0.78570200	4.71568100
H	-2.98201900	-2.84208200	2.82438900
H	-2.34812700	-1.58778400	4.87150300
C	-3.60103000	3.56482100	0.42997000
H	-3.13583200	3.60785400	1.42122400
H	-2.86598800	3.89348600	-0.31113200
H	-4.44142700	4.26831100	0.41905900

Cartesian coordinates of the optimized geometries for **16** ([Au]$^+$ = JohnPhosAu$^+$) at B3LYP-D3BJ/6-31G(d),def2-TZVP+ECP level of theory (number of imaginary frequencies = 0):

C	-1.61766400	0.76575900	-0.70090500
C	-2.41589600	0.98569400	0.38657600
Au	0.25609800	-0.05481800	-0.53980800
P	2.33152400	-1.10709000	-0.19988000
C	2.23596700	-1.99569800	1.47383900
C	2.67365800	-2.25438000	-1.67850600
C	1.80668000	-0.91255400	2.48509500
H	0.83877200	-0.47251900	2.23219800
H	1.72011500	-1.36739800	3.47872800
H	2.54864800	-0.10993800	2.54711600
C	3.55485400	-2.61631700	1.96013300
H	3.36925300	-3.11612000	2.91841900
H	3.95224800	-3.36569800	1.27234400
H	4.32077800	-1.85651700	2.13071600
C	1.14833100	-3.08096000	1.39409800
H	0.20389200	-2.68097300	1.01319700
H	1.45364700	-3.92150300	0.76417600
H	0.96413900	-3.47354700	2.40111300
C	1.33012500	-2.86980800	-2.12431800
H	0.61074100	-2.09891000	-2.41599000
H	1.51002600	-3.51423900	-2.99293500
H	0.87176600	-3.48248700	-1.34409000
C	3.66694500	-3.39996200	-1.42732300
H	3.30639200	-4.09642900	-0.66582100
H	3.78422500	-3.96776300	-2.35801100
H	4.66068400	-3.05154300	-1.14004000
C	3.19168900	-1.34463200	-2.80975500
H	2.50934100	-0.50671000	-2.99410200
H	4.18227000	-0.93831200	-2.59097700
H	3.25834900	-1.92954600	-3.73440800
C	3.76685400	0.03467200	-0.04964800
C	5.06160500	-0.51164700	0.04096100
C	3.62392800	1.44014200	0.00364800
C	6.18900600	0.29017200	0.18523000
H	5.19606400	-1.58362500	0.00469500
C	4.77238800	2.23362400	0.16080300
C	6.04325100	1.67492400	0.25105600
H	7.17181500	-0.16644500	0.24879400
H	4.65021800	3.31147300	0.20212300
H	6.91161000	2.31606300	0.36758500
C	2.33610500	2.18985400	-0.11592200
C	1.86718400	2.58232000	-1.37942000
C	1.66021800	2.63659600	1.02753300

C	0.74460500	3.40226900	-1.49651800
H	2.39827300	2.25347500	-2.26766100
C	0.53723000	3.45723200	0.90894200
H	2.02450900	2.34873000	2.00873500
C	0.08040200	3.84469600	-0.35122100
H	0.39438600	3.69895700	-2.47997900
H	0.03542400	3.81144300	1.80482800
H	-0.77852100	4.50310500	-0.44318000
C	-3.68613300	1.83649800	0.32781600
C	-4.49992900	0.92045500	-0.04302100
C	-5.25101000	-0.18211800	-0.42245900
C	-5.44314100	-0.47445200	-1.79992200
C	-5.81581800	-1.02626300	0.57237100
C	-6.18620300	-1.58610900	-2.16295000
H	-4.99748300	0.17756300	-2.54240700
C	-6.55224600	-2.13477900	0.18859100
H	-5.65137700	-0.79147500	1.61840000
C	-6.73820500	-2.41127300	-1.17342400
H	-6.34097400	-1.81800100	-3.21138200
H	-6.98687300	-2.78653100	0.93915200
H	-7.32113100	-3.27924600	-1.46658200
Cl	-2.17028900	1.34747700	-2.27237000
C	-2.09839000	0.37700200	1.70538200
C	-1.74748200	1.17448200	2.80393700
C	-2.16179900	-1.01645300	1.86621200
C	-1.45600400	0.58963100	4.03624900
H	-1.67237400	2.25001900	2.68280700
C	-1.87785700	-1.59871200	3.10020800
H	-2.43034300	-1.63384700	1.01444000
C	-1.52472900	-0.79664700	4.18793800
H	-1.17279200	1.21589400	4.87680200
H	-1.92681400	-2.67776500	3.21173100
H	-1.30051800	-1.25000200	5.14869900
C	-3.76421000	3.29378300	0.64439100
H	-3.47545900	3.46162500	1.68720800
H	-3.05654000	3.83337800	0.00752500
H	-4.77195400	3.68297000	0.48518900

Cartesian coordinates of the optimized geometries for **17** ([Au]$^+$ = JohnPhosAu$^+$) at B3LYP-D3BJ/6-31G(d),def2-TZVP+ECP level of theory (number of imaginary frequencies = 1):

C	1.69751100	0.30983000	-0.12608700
C	2.37630900	-0.75217200	-0.61713000
Au	-0.30453900	0.26129200	0.34868600
P	-2.55422000	0.04160400	0.98670600
C	-2.91793400	-1.81525800	1.12593000
C	-2.81032400	1.00778500	2.60734800
C	-2.51717500	-2.40780900	-0.24026100
H	-1.46251900	-2.23648100	-0.46867900
H	-2.68616500	-3.49091700	-0.22244300
H	-3.12123900	-1.98662900	-1.05047300
C	-4.38556500	-2.17868500	1.39963100
H	-4.46353400	-3.27016000	1.47252100
H	-4.76096000	-1.76123400	2.33615700
H	-5.04048700	-1.85796600	0.58627700
C	-2.01813100	-2.41220500	2.22204800
H	-0.96678200	-2.14929600	2.07028600
H	-2.32098300	-2.08962100	3.22253000
H	-2.09729100	-3.50535900	2.18986400
C	-1.52386700	0.87122200	3.44950500
H	-0.65285600	1.27174200	2.92282500
H	-1.65113600	1.43756000	4.37984600
H	-1.30838200	-0.16618700	3.71710900
C	-3.99886100	0.56795600	3.47722000
H	-3.88861000	-0.45986500	3.83281800

H	-4.03965800	1.21526100	4.36145400
H	-4.96133900	0.66054400	2.97075600
C	-2.97132700	2.48565600	2.20128200
H	-2.14561400	2.81621100	1.56054500
H	-3.90976600	2.66798500	1.67188400
H	-2.96164700	3.10658300	3.10467200
C	-3.79020000	0.67091300	-0.22500700
C	-5.14955300	0.69265800	0.14136100
C	-3.43732100	1.10127100	-1.52496100
C	-6.14157900	1.11702100	-0.73657200
H	-5.44425700	0.36566300	1.12863700
C	-4.45349800	1.51392100	-2.40272000
C	-5.79160700	1.52592300	-2.02238600
H	-7.17910700	1.12286500	-0.41709300
H	-4.17022600	1.83993600	-3.39868700
H	-6.55283300	1.85572900	-2.72278800
C	-2.04639600	1.17963600	-2.06580600
C	-1.26639500	2.32553200	-1.84589500
C	-1.55439900	0.17651000	-2.91065500
C	-0.01559700	2.45883800	-2.44891400
H	-1.65251900	3.11553900	-1.20870800
C	-0.30284900	0.31163300	-3.51369500
H	-2.15898400	-0.70539100	-3.09871600
C	0.46982400	1.45016600	-3.28275000
H	0.57811300	3.34831800	-2.26487300
H	0.05692900	-0.46628900	-4.18140400
H	1.44045400	1.55853400	-3.75808200
C	3.79064500	-0.73094000	-1.13730800
C	4.70482600	-0.12465500	-0.45662300
C	5.72826900	0.43811200	0.26815400
C	6.06873800	1.81361100	0.09780000
C	6.47831300	-0.37106100	1.17387500
C	7.13587000	2.34513700	0.79663900
H	5.47069400	2.41833800	-0.57392900
C	7.53527400	0.18195700	1.87428200
H	6.20530100	-1.41390500	1.29375800
C	7.86223800	1.53280800	1.68270100
H	7.40727700	3.38786800	0.67178400
H	8.11141700	-0.42352500	2.56562100
H	8.69332800	1.96126600	2.23520800
Cl	2.53628500	1.87006600	0.03644600
C	1.71286700	-2.09082300	-0.64879100
C	1.16879700	-2.60861900	-1.83377500
C	1.61409700	-2.84374400	0.53033500
C	0.53829500	-3.85256800	-1.83775700
H	1.20968600	-2.01568200	-2.74055000
C	0.98113300	-4.08610400	0.52661100
H	2.02567000	-2.43928100	1.45018100
C	0.44644500	-4.59525000	-0.65888900
H	0.11058000	-4.23722600	-2.75882300
H	0.90438300	-4.65572800	1.44792100
H	-0.04509900	-5.56343500	-0.66293800
C	4.13149700	-1.44786000	-2.43329000
H	3.84999000	-2.50059600	-2.34629600
H	3.54861200	-1.00017500	-3.24474400
H	5.19438900	-1.37426500	-2.67247900

Cartesian coordinates of the optimized geometries for **18** ([Au]$^+$ = JohnPhosAu$^+$) at B3LYP-D3BJ/6-31G(d),def2-TZVP+ECP level of theory (number of imaginary frequencies = 0):

C	1.54738700	0.31882700	-0.47051800
C	2.29413800	1.27246400	0.13334200
Au	-0.44953800	-0.00509300	-0.64146400
P	-2.79387900	-0.02554400	-0.67979600
C	-3.34724500	1.72744500	-0.20795400

C	-3.37896800	-0.59963700	-2.39692200
C	-2.63899500	2.04190900	1.12622900
H	-1.54986100	2.01718500	1.03330200
H	-2.92316600	3.05001600	1.44910300
H	-2.93988500	1.34149300	1.91190000
C	-4.86132700	1.89034900	-0.00122700
H	-5.06523200	2.93964300	0.24276700
H	-5.44222300	1.64344500	-0.89214800
H	-5.22344600	1.28457600	0.83256800
C	-2.85767300	2.70861000	-1.28754300
H	-1.78611900	2.59897800	-1.48108700
H	-3.39929200	2.58725800	-2.22992400
H	-3.03012200	3.73325700	-0.93822700
C	-2.39135200	-0.04560300	-3.44551800
H	-1.37384000	-0.40884100	-3.27300200
H	-2.70831900	-0.38298800	-4.43921200
H	-2.36219200	1.04652100	-3.45824100
C	-4.79918700	-0.16920100	-2.79714300
H	-4.89464900	0.91746300	-2.86599800
H	-5.01818600	-0.57857100	-3.79036700
H	-5.56864600	-0.54604500	-2.12073600
C	-3.27446600	-2.13723900	-2.39132100
H	-2.27769200	-2.47090200	-2.07954900
H	-4.01160500	-2.59840100	-1.72963300
H	-3.44728100	-2.50856400	-3.40795500
C	-3.56687200	-1.13804800	0.56426300
C	-4.96602200	-1.29695200	0.54592300
C	-2.82073000	-1.84631500	1.53458900
C	-5.62321400	-2.12491600	1.44990800
H	-5.55739400	-0.76412900	-0.18554600
C	-3.50195200	-2.67455500	2.44170400
C	-4.88531100	-2.81879800	2.40751500
H	-6.70318000	-2.22443600	1.40443600
H	-2.91974800	-3.21286000	3.18307800
H	-5.38144700	-3.46850200	3.12186700
C	-1.33384400	-1.80959000	1.69090000
C	-0.54069100	-2.79814900	1.08817200
C	-0.72553500	-0.88487600	2.55121900
C	0.83245800	-2.85258400	1.33277100
H	-1.01026800	-3.53160500	0.43935000
C	0.64779200	-0.94031500	2.79361400
H	-1.33309400	-0.12806000	3.03662400
C	1.43002500	-1.92317000	2.18645900
H	1.43011600	-3.63085900	0.86673800
H	1.10128600	-0.22336000	3.47102000
H	2.49696600	-1.97031500	2.38572900
C	3.73301900	1.06849400	0.27176600
C	4.20876500	-0.08228300	-0.25593500
C	5.51735200	-0.70179900	-0.41443100
C	5.72235600	-2.05332600	-0.08564500
C	6.59495200	0.06497200	-0.89168700
C	6.98457200	-2.62221600	-0.22214600
H	4.89472100	-2.64885500	0.28938500
C	7.85910300	-0.50779800	-1.01103800
H	6.43219400	1.09795300	-1.18048500
C	8.05570000	-1.84967400	-0.67947200
H	7.13600900	-3.66557600	0.03572400
H	8.68693000	0.09042300	-1.37834800
H	9.04027200	-2.29507400	-0.78179400
Cl	2.80478400	-0.98992200	-0.97403200
C	1.56588200	2.46249400	0.65285600
C	1.42935000	2.67048900	2.03266300
C	0.95995700	3.35721400	-0.23994500
C	0.70172700	3.75931600	2.51053100
H	1.87694400	1.96682200	2.72768400
C	0.23231300	4.44605000	0.24064500

H	1.06444000	3.19268000	-1.30774400
C	0.10244800	4.64893700	1.61536600
H	0.59855500	3.91098900	3.58068500
H	-0.23094300	5.13499900	-0.45903800
H	-0.46271400	5.49709900	1.98961500
C	4.61307000	2.07231800	0.96310500
H	4.68142000	2.98982600	0.36715300
H	4.18736400	2.35219800	1.93081200
H	5.61983100	1.68220700	1.12008400

Cartesian coordinates of the optimized geometries for **19** ([Au]$^+$ = JohnPhosAu$^+$) at B3LYP-D3BJ/6-31G(d),def2-TZVP+ECP level of theory (number of imaginary frequencies = 1):

C	1.14254200	1.12434300	-0.16282700
C	2.36145400	0.83717000	0.02594800
Au	-0.81881700	0.62734800	-0.32025300
C	3.68427400	0.32970000	0.16397400
C	4.05262600	-0.74282400	-0.58952100
C	5.37381000	-1.38746600	-0.59418200
C	6.53732600	-0.60508400	-0.69465000
C	5.49357500	-2.78554800	-0.51875600
C	7.79272500	-1.20855300	-0.69862800
H	6.45257900	0.47137100	-0.80073000
C	6.75031200	-3.38257300	-0.51235500
H	4.59999900	-3.39807200	-0.45924700
C	7.90231900	-2.59674300	-0.60102600
H	8.68386000	-0.59528400	-0.78913200
H	6.83194500	-4.46274100	-0.44243300
H	8.88123600	-3.06607200	-0.60381700
P	-3.06304100	0.00687700	-0.56560800
C	-4.15652800	1.45126500	-0.01097700
C	-3.31300500	-0.51764400	-2.37942100
C	1.87567000	2.50573700	0.26905900
C	2.38004300	3.38459000	-0.71098700
C	1.72991700	2.94116300	1.60361300
C	2.72538500	4.68423200	-0.35835200
H	2.49399900	3.02888700	-1.72968700
C	2.06293100	4.24552800	1.94264200
H	1.34415900	2.24418500	2.34020600
C	2.56301900	5.11386900	0.96358000
H	3.11727200	5.36462500	-1.10749100
H	1.94379100	4.59023600	2.96484700
H	2.83076800	6.13069700	1.23421100
Cl	2.85702800	-1.48579800	-1.64533300
C	4.56068600	0.97626300	1.21335100
H	4.90251200	1.96570000	0.88787800
H	3.99089800	1.11699800	2.13716000
H	5.43356200	0.36095200	1.43028500
C	-3.90638000	2.64643200	-0.94731100
H	-4.40132800	3.53235200	-0.53299200
H	-2.83898500	2.87214600	-1.04487400
H	-4.31833600	2.47855600	-1.94636200
C	-3.68007700	1.80748600	1.41183900
H	-2.62790600	2.10962900	1.42663900
H	-4.28088500	2.64279600	1.78977200
H	-3.80820800	0.96529100	2.09928600
C	-5.66083100	1.14001300	0.04959500
H	-6.18946700	2.05091300	0.35424800
H	-6.07111800	0.83154300	-0.91401500
H	-5.88510600	0.36874800	0.78992900
C	-2.47690000	0.42275100	-3.27309000
H	-1.41110300	0.36673000	-3.03252800
H	-2.60476100	0.11723600	-4.31807100
H	-2.78829200	1.46670000	-3.19136900
C	-2.73435900	-1.94165100	-2.49384700

H	-2.70969200	-2.22969200	-3.55104100
H	-1.70809300	-1.99016700	-2.11132500
H	-3.33661900	-2.67629100	-1.95397200
C	-4.76490200	-0.49673100	-2.88320100
H	-5.19082200	0.50981700	-2.86730700
H	-4.77409600	-0.83703300	-3.92533500
H	-5.42254300	-1.16512500	-2.32445800
C	-3.54518200	-1.42675100	0.47990100
C	-4.82851900	-1.98028900	0.30429700
C	-2.67857500	-2.02007600	1.42713100
C	-5.25776900	-3.08658100	1.02945800
H	-5.50855600	-1.54232300	-0.41269400
C	-3.13013000	-3.13764200	2.14937300
C	-4.40050400	-3.67176600	1.95974600
H	-6.25352500	-3.48649200	0.86570100
H	-2.45795300	-3.58582100	2.87435200
H	-4.71728100	-4.53661400	2.53442400
C	-1.28893200	-1.57376100	1.75739700
C	-1.06576400	-0.61505000	2.75705100
C	-0.18545100	-2.23717700	1.19774600
C	0.23118700	-0.32846600	3.18747600
H	-1.91382100	-0.11491300	3.21374000
C	1.11009100	-1.94714900	1.62623000
H	-0.35143300	-2.99122800	0.43421700
C	1.32081200	-0.99678600	2.62627300
H	0.38420000	0.39776900	3.98127000
H	1.95201200	-2.46818300	1.18211600
H	2.32841200	-0.78834400	2.97410400

Cartesian coordinates of the optimized geometries for **20** ([Au]$^+$ = JohnPhosAu$^+$) at B3LYP-D3BJ/6-31G(d),def2-TZVP+ECP level of theory (number of imaginary frequencies = 0):

C	0.76725500	2.42632300	0.00819600
C	1.67919600	1.58601400	-0.09038300
C	-0.21758900	3.45368300	0.14311000
Cl	2.36888700	-1.10524400	-1.39601800
C	-0.68687400	3.80226500	1.42379800
C	-0.74582000	4.08788200	-0.99733900
C	-1.73232100	5.05772700	-0.85186700
C	-2.19640700	5.40148300	0.42134500
C	-1.67118200	4.77643200	1.55601700
H	-2.03222100	5.04758100	2.54306500
H	-0.28157200	3.29435900	2.29199000
H	-0.37739400	3.81064300	-1.97961400
H	-2.13883500	5.54853800	-1.73043000
Au	-0.21121100	0.37498200	-0.34499900
C	3.03152700	1.11735800	0.01326800
C	3.48176900	-0.03450200	-0.53648800
C	4.86579000	-0.53734200	-0.49031900
C	5.93394900	0.29168400	-0.86879200
C	5.13295300	-1.85191600	-0.07260900
C	7.24442000	-0.17866400	-0.80849900
H	5.73156400	1.29455800	-1.23030600
C	6.44402300	-2.31374200	-0.00427700
H	4.31069000	-2.50676800	0.19837800
C	7.50258800	-1.47863000	-0.37113000
H	8.06187500	0.46782100	-1.11222300
H	6.64076700	-3.32744800	0.33064000
H	8.52386100	-1.84385000	-0.32481500
H	-2.96684300	6.15863600	0.52937900
P	-2.15661100	-0.84634300	-0.70956800
C	-3.60424500	0.38142000	-0.70576300
C	-1.94062700	-1.81094700	-2.33513200
C	3.91102700	2.02819100	0.84945500
H	4.14364400	2.95205600	0.30735700

H	3.37746100	2.30972800	1.76258000
H	4.84756300	1.54129500	1.12253400
C	-1.15829000	-0.92198600	-3.32526100
H	-0.16665200	-0.66200300	-2.94536200
H	-1.02457200	-1.47666100	-4.26104100
H	-1.68552100	0.00530000	-3.56155100
C	-1.08016700	-3.04249400	-1.99017200
H	-0.79307100	-3.54494000	-2.92077100
H	-0.15968300	-2.75993700	-1.46698600
H	-1.61938200	-3.76011600	-1.36716300
C	-3.24441300	-2.25526100	-3.01861900
H	-2.98396600	-2.82171500	-3.92019900
H	-3.85545900	-2.91151200	-2.39630300
H	-3.85319700	-1.40452500	-3.33528700
C	-3.43664300	1.35762500	-1.88236400
H	-4.17932900	2.15779200	-1.78532100
H	-2.44662100	1.82458800	-1.88652400
H	-3.60058000	0.87207100	-2.84834600
C	-3.49942400	1.15953800	0.62121000
H	-2.57086000	1.73194900	0.69153300
H	-4.33535000	1.86595000	0.68141600
H	-3.56379700	0.49064100	1.48520500
C	-4.98883200	-0.28509600	-0.76670200
H	-5.74937800	0.50416600	-0.78322100
H	-5.13078700	-0.89395300	-1.66157000
H	-5.17860600	-0.90409700	0.11308100
C	-2.52217000	-2.05734400	0.62120800
C	-3.59129700	-2.95455600	0.43318000
C	-1.78731800	-2.12298500	1.82780300
C	-3.93826900	-3.89374700	1.39845900
H	-4.16856000	-2.91955300	-0.48026200
C	-2.15766700	-3.07349700	2.79325300
C	-3.21710600	-3.95219900	2.58997900
H	-4.76688300	-4.57151800	1.21913800
H	-1.58917700	-3.11554900	3.71697500
H	-3.47530700	-4.67726200	3.35552900
C	-0.61683000	-1.26475500	2.18890900
C	0.68818200	-1.68588300	1.88522800
C	-0.79373800	-0.11212700	2.96759500
C	1.79125400	-0.96603100	2.34750300
H	0.83219300	-2.58901100	1.30015400
C	0.31059300	0.60904400	3.42470100
H	-1.79860200	0.20737200	3.22520500
C	1.60429900	0.18236600	3.11891600
H	2.79452400	-1.30606000	2.11155300
H	0.15970800	1.48907000	4.04360800
H	2.46257500	0.73108900	3.49421500

Cartesian coordinates of the optimized geometries for **21** ([Au]$^+$ = JohnPhosAu$^+$) at B3LYP-D3BJ/6-31G(d),def2-TZVP+ECP level of theory (number of imaginary frequencies = 1):

C	-1.61365700	-1.04721500	-0.12436600
C	-2.08124200	-0.95551500	-1.30930300
C	-2.13599800	-1.34634900	1.20868300
Cl	-1.82200700	-0.63282500	-2.92534600
C	-3.01726700	-2.41938800	1.41702900
C	-1.76693700	-0.54108400	2.29973800
C	-2.30982100	-0.77479700	3.56067900
C	-3.20375300	-1.83003500	3.75695200
C	-3.54597300	-2.65660100	2.68404300
H	-4.22701400	-3.48890300	2.83312400
H	-3.28249000	-3.06410200	0.58902800
H	-1.07995500	0.28111600	2.13988700
H	-2.02987500	-0.13565500	4.39269300
Au	0.48878500	-0.65103700	-0.22975300

H	-3.62192200	-2.01534500	4.74147100
P	2.80592300	-0.46880200	-0.04713900
C	3.57240100	-1.11446800	-1.65573700
C	3.29507100	-1.44174500	1.51528500
C	2.90181000	-0.30920400	-2.78707200
H	1.81835600	-0.46391700	-2.81141100
H	3.31493000	-0.63548600	-3.74844500
H	3.09732000	0.76328000	-2.68624800
C	5.09469300	-0.92874000	-1.77062600
H	5.42484000	-1.37717700	-2.71492800
H	5.64600200	-1.41983000	-0.96607900
H	5.37310400	0.12706000	-1.79568000
C	3.21713800	-2.60391400	-1.80781800
H	2.14313800	-2.78285400	-1.68858500
H	3.75546600	-3.22921400	-1.09013800
H	3.50398300	-2.93566100	-2.81226100
C	2.41822400	-2.70817700	1.60585200
H	1.35480000	-2.46022900	1.67495100
H	2.69543900	-3.25931800	2.51188800
H	2.55610200	-3.37805400	0.75430500
C	4.77101100	-1.86498000	1.58514600
H	5.03923400	-2.55626100	0.78192800
H	4.93615100	-2.38914800	2.53367800
H	5.45852800	-1.01740800	1.56557500
C	2.95251800	-0.52594400	2.70713500
H	1.91247000	-0.18229100	2.66344200
H	3.60289700	0.35076500	2.75404800
H	3.07664500	-1.09458500	3.63574900
C	3.42707700	1.24513700	0.19025100
C	4.80507700	1.42646000	0.42085600
C	2.58093000	2.37830200	0.23062200
C	5.34559500	2.67952600	0.68967800
H	5.47189600	0.57645500	0.39574800
C	3.14496800	3.63376800	0.51142900
C	4.50803100	3.79260500	0.73961600
H	6.41244300	2.78117900	0.86210100
H	2.48708400	4.49669500	0.53940400
H	4.91212600	4.77769100	0.95139800
C	1.10660600	2.37077500	-0.00553200
C	0.22437700	2.53235400	1.07317100
C	0.58591700	2.33802200	-1.30799400
C	-1.15070900	2.63596900	0.85463500
H	0.62540800	2.58083800	2.08173300
C	-0.78829000	2.45159300	-1.52357200
H	1.26391500	2.24348600	-2.15009100
C	-1.66089200	2.59370900	-0.44343000
H	-1.82758300	2.75557100	1.69505200
H	-1.17567300	2.43730700	-2.53751800
H	-2.72706200	2.68575800	-0.61024000
C	-4.55301300	-0.62988700	-1.03952500
C	-4.28298800	-1.71966800	-1.52755300
C	-4.82325900	0.64719400	-0.47954800
C	-5.15329500	1.72510900	-1.32523600
C	-4.75645300	0.84506600	0.91370200
C	-5.40465700	2.98157700	-0.78257400
H	-5.20992100	1.56230100	-2.39648500
C	-4.99910000	2.10856000	1.44281900
H	-4.51483400	0.01116600	1.56115900
C	-5.32132200	3.17653000	0.59969800
H	-5.66307900	3.80969600	-1.43493400
H	-4.94569300	2.25956300	2.51658300
H	-5.51430400	4.15905200	1.01942300
C	-4.23489400	-3.04078100	-2.15089800
H	-3.71524500	-2.99881200	-3.11436300
H	-3.71269500	-3.76538400	-1.51717400
H	-5.25119100	-3.40991000	-2.32872700

Cartesian coordinates of the optimized geometries for **22** ([Au]$^{+}$ = JohnPhosAu^{+}) at B3LYP-D3BJ/6-31G(d),def2-TZVP+ECP level of theory (number of imaginary frequencies = 0):

C	-1.55838600	-1.14469300	-0.08024000
C	-2.19089300	-1.41020200	-1.26211800
C	-2.29239400	-1.20968100	1.19717200
Cl	-1.34967500	-1.26892000	-2.81284500
C	-3.20555000	-2.23463600	1.51260100
C	-2.06713400	-0.20158500	2.15518600
C	-2.77318700	-0.18602100	3.35660300
C	-3.68700900	-1.20071700	3.64839300
C	-3.88660300	-2.23458200	2.72743700
H	-4.57042800	-3.04550700	2.96012800
H	-3.35531600	-3.05354500	0.81755200
H	-1.34814600	0.57825000	1.93199200
H	-2.59502400	0.61019600	4.07352100
Au	0.46224700	-0.69995700	-0.01414300
H	-4.22266300	-1.20159000	4.59278300
P	2.80620600	-0.45503800	0.06743100
C	3.49794800	-1.36614100	-1.44793300
C	3.39719800	-1.14437100	1.74132300
C	2.78089200	-0.73850700	-2.66136000
H	1.69659200	-0.87409900	-2.60988400
H	3.14181600	-1.22153600	-3.57702800
H	2.99580000	0.33242900	-2.74302400
C	5.01371400	-1.24860500	-1.67573800
H	5.28032000	-1.87073200	-2.53842600
H	5.60291800	-1.60043500	-0.82623800
H	5.31050000	-0.22418300	-1.91069200
C	3.10981900	-2.85187400	-1.33854300
H	2.04172700	-2.98178200	-1.13485600
H	3.67822900	-3.36566800	-0.55788400
H	3.33470200	-3.34860600	-2.28953100
C	2.54006100	-2.37790200	2.09303600
H	1.47695800	-2.12709100	2.15393100
H	2.85714700	-2.75710800	3.07171500
H	2.65468600	-3.18818300	1.36950000
C	4.87876000	-1.54408500	1.81104000
H	5.11529300	-2.36386700	1.12731000
H	5.09777800	-1.89472400	2.82668100
H	5.55342700	-0.70986000	1.60905000
C	3.10323300	-0.03719800	2.77313700
H	2.05975500	0.29470800	2.71880400
H	3.74844100	0.83418000	2.63568600
H	3.27568700	-0.43513400	3.77986700
C	3.46173100	1.26389900	-0.02563500
C	4.85523800	1.45570400	0.04779200
C	2.63016400	2.40687400	-0.08574100
C	5.42583900	2.72439000	0.05785700
H	5.51218000	0.60018100	0.10579800
C	3.22401800	3.68041600	-0.06730500
C	4.60311900	3.84771600	0.00053900
H	6.50471000	2.83044500	0.11471300
H	2.57565300	4.54975000	-0.11552400
H	5.02987600	4.84596400	0.00902100
C	1.13878000	2.39836200	-0.14649700
C	0.40714400	2.84119300	0.96531900
C	0.45073700	2.08087400	-1.32702100
C	-0.98087300	2.96282400	0.89912900
H	0.93649800	3.09787000	1.87822000
C	-0.93895300	2.20603000	-1.39240000
H	1.00798400	1.75572000	-2.19936900
C	-1.65624800	2.64784200	-0.28097000
H	-1.53382200	3.31241800	1.76654200

H	-1.45453500	1.95610100	-2.31468600
H	-2.73301300	2.75638300	-0.33375700
C	-4.17762400	-0.62296300	-1.17974100
C	-3.63891300	-1.75426500	-1.47516600
C	-4.65671500	0.60546400	-0.76625500
C	-4.86979600	1.64533900	-1.71498100
C	-4.93215100	0.82679900	0.61295900
C	-5.33995700	2.87405000	-1.28646400
H	-4.65548600	1.45682500	-2.76138600
C	-5.40122200	2.06649000	1.01937400
H	-4.77921000	0.02264900	1.32146600
C	-5.60370200	3.08279200	0.07707400
H	-5.50539100	3.67421200	-1.99997600
H	-5.61371900	2.24639800	2.06784100
H	-5.97443900	4.04937200	0.40495300
C	-4.15948900	-3.05616700	-1.99861800
H	-3.72969700	-3.24559100	-2.98768800
H	-3.84484600	-3.87103500	-1.33840500
H	-5.24868100	-3.04717500	-2.07399400

Cartesian coordinates of the optimized geometries for **23** ([Au]$^+$ = JohnPhosAu$^+$) at B3LYP-D3BJ/6-31G(d),def2-TZVP+ECP level of theory (number of imaginary frequencies = 1):

C	1.34984900	1.06027000	-0.41873300
C	2.40066900	0.28799600	-0.74553300
C	1.52118800	2.43551500	0.07208100
Cl	2.19998000	-1.45461900	-1.04432800
C	0.77782200	3.49508700	-0.48107900
C	2.39237900	2.71512900	1.14234700
C	2.53406300	4.01366200	1.62586600
C	1.81310000	5.06050700	1.04512000
C	0.93633500	4.79532000	-0.01118700
H	0.36962300	5.60420000	-0.46255400
H	0.08970300	3.28621300	-1.29447900
H	2.94509400	1.89878700	1.59563200
H	3.20532600	4.21122900	2.45680800
Au	-0.59929400	0.34386400	-0.48757600
H	1.92484200	6.07330100	1.41985600
P	-2.83275000	-0.37557500	-0.68112400
C	-2.87456200	-1.85308400	-1.86866500
C	-3.86940800	1.11673300	-1.25384800
C	-1.86491400	-2.86635100	-1.29119600
H	-0.85528400	-2.44869400	-1.23295700
H	-1.83510800	-3.74868500	-1.94151000
H	-2.16116600	-3.19847100	-0.29084400
C	-4.23556000	-2.55366900	-2.00499200
H	-4.13509800	-3.36547400	-2.73537400
H	-5.02386300	-1.88828400	-2.36320200
H	-4.55616200	-3.00107600	-1.06145800
C	-2.38833500	-1.38311900	-3.25091700
H	-1.43681600	-0.84480500	-3.18563000
H	-3.12068200	-0.73837200	-3.74544200
H	-2.23689900	-2.25947900	-3.89203500
C	-3.03003100	1.93066800	-2.26071000
H	-2.09433700	2.27668700	-1.81268900
H	-3.60734700	2.81016600	-2.56966900
H	-2.78305300	1.36096900	-3.15955400
C	-5.21907100	0.78715600	-1.91056100
H	-5.09709600	0.21625600	-2.83485200
H	-5.71707800	1.72824200	-2.17303700
H	-5.89524400	0.24330500	-1.24852700
C	-4.09122600	1.98128900	0.00290600
H	-3.14362900	2.20625000	0.50615500
H	-4.75541700	1.49742000	0.72318200
H	-4.54573000	2.93316800	-0.29564500

C	-3.60003500	-0.97244600	0.88294700
C	-4.96798600	-1.30742400	0.88538100
C	-2.87964900	-1.07961800	2.09496800
C	-5.61882400	-1.73257700	2.03892200
H	-5.53992900	-1.23665100	-0.02889200
C	-3.55301900	-1.51042500	3.24987100
C	-4.90581800	-1.83440700	3.23236500
H	-6.67507200	-1.98072800	2.00249500
H	-2.98887300	-1.58831600	4.17407700
H	-5.39780700	-2.16358800	4.14252800
C	-1.42890400	-0.76703300	2.27132000
C	-1.03499600	0.48023900	2.77925400
C	-0.45465600	-1.75113300	2.05521500
C	0.30719200	0.74022900	3.05615500
H	-1.78793100	1.24190400	2.95867200
C	0.88769400	-1.49063000	2.33288100
H	-0.75344500	-2.72229400	1.67410600
C	1.26942000	-0.24657300	2.83522100
H	0.59969100	1.71352700	3.43699300
H	1.63117500	-2.26142400	2.15566700
H	2.31348300	-0.04800700	3.06040700
C	4.75264200	-0.08108400	-0.57894100
C	3.81927000	0.74772800	-0.92884200
C	5.69960600	-1.03366600	-0.30298300
C	6.05787300	-1.99512800	-1.29834700
C	6.33898000	-1.06576800	0.97419300
C	7.02650700	-2.94068500	-1.01935500
H	5.55008500	-1.96748200	-2.25601300
C	7.30036500	-2.02392100	1.23610200
H	6.05653100	-0.33027100	1.71955400
C	7.64319400	-2.95439300	0.24215400
H	7.30692800	-3.67435800	-1.76751400
H	7.79150400	-2.05779500	2.20265800
H	8.40087900	-3.70287600	0.45454200
C	4.14618900	2.10053600	-1.54950500
H	3.40577200	2.31255700	-2.32419700
H	4.10041800	2.89493200	-0.80343400
H	5.14377700	2.08533900	-1.99417300

Cartesian coordinates of the optimized geometries for **24** ([Au]$^+$ = JohnPhosAu$^+$) at B3LYP-D3BJ/6-31G(d),def2-TZVP+ECP level of theory (number of imaginary frequencies = 0):

C	1.32341700	1.14855300	-0.16669900
C	2.42591100	0.38135200	-0.20898800
C	1.38585300	2.59972700	0.08194600
Cl	2.32615400	-1.29236800	-0.84051600
C	2.12906100	3.43120100	-0.77547900
C	0.65030500	3.19044700	1.12540100
C	0.70973600	4.56481200	1.34076500
C	1.46793000	5.38003500	0.49549200
C	2.16636800	4.80937100	-0.57116300
H	2.73869500	5.43918500	-1.24614900
H	2.66341100	2.98554500	-1.60976900
H	0.04942900	2.55621900	1.76772800
H	0.15328800	5.00479300	2.16330500
Au	-0.57155100	0.35818000	-0.44298100
H	1.50043900	6.45312200	0.65705400
P	-2.77108900	-0.39465000	-0.80699200
C	-2.72356900	-1.76754000	-2.11377500
C	-3.80112900	1.12829200	-1.31013500
C	-1.72706700	-2.81330200	-1.57309500
H	-0.73080700	-2.38734300	-1.42007000
H	-1.64275300	-3.63227600	-2.29726900
H	-2.07253300	-3.24052100	-0.62584200
C	-4.06083200	-2.47483600	-2.38563900

H	-3.90626700	-3.21141100	-3.18323200
H	-4.84449700	-1.79182400	-2.72000600
H	-4.41841300	-3.01628400	-1.50681700
C	-2.17454300	-1.17276600	-3.42284100
H	-1.23924800	-0.62634800	-3.26135000
H	-2.89303700	-0.49961100	-3.89919200
H	-1.97186900	-1.98834900	-4.12689900
C	-2.92585900	2.03121800	-2.20493800
H	-2.01933100	2.35719000	-1.68657100
H	-3.50378900	2.92361300	-2.47298600
H	-2.62562300	1.53843500	-3.13251900
C	-5.11272600	0.83559800	-2.05492100
H	-4.93801800	0.34333600	-3.01533500
H	-5.61364000	1.78825900	-2.26461000
H	-5.80950600	0.23107300	-1.47122100
C	-4.09709900	1.88549300	-0.00034100
H	-3.17874300	2.07853800	0.56637100
H	-4.79019900	1.33888400	0.64392300
H	-4.54826300	2.85418300	-0.24469300
C	-3.61288500	-1.11656900	0.66430500
C	-4.97005200	-1.47660900	0.55287700
C	-2.98329700	-1.25365900	1.92396100
C	-5.69918200	-1.94948000	1.63900100
H	-5.47346000	-1.38161100	-0.39855700
C	-3.73816200	-1.72496300	3.01154200
C	-5.07929000	-2.07022900	2.88140700
H	-6.74401700	-2.21596000	1.51316400
H	-3.24454300	-1.82590500	3.97311600
H	-5.63302300	-2.43324400	3.74185900
C	-1.55267500	-0.94657000	2.22595600
C	-1.22827400	0.18076100	2.99513900
C	-0.53318800	-1.84673000	1.88344800
C	0.08809000	0.41048100	3.39830400
H	-2.01643100	0.87138500	3.28074400
C	0.78079800	-1.61958600	2.29221900
H	-0.77685400	-2.73319300	1.30767300
C	1.09584300	-0.48858800	3.04565000
H	0.32298000	1.28563900	3.99728800
H	1.55577500	-2.32723500	2.01621200
H	2.11843300	-0.31438900	3.36863100
C	4.72670400	-0.22217600	0.00186200
C	3.79643600	0.65667700	0.26038500
C	5.77060400	-1.09217700	-0.19158800
C	6.63842100	-0.93211300	-1.31518100
C	6.00060400	-2.15815600	0.73183800
C	7.69538300	-1.80514100	-1.49662600
H	6.44802600	-0.12247900	-2.01113800
C	7.06690500	-3.01581000	0.53838800
H	5.32529700	-2.27489000	1.57232400
C	7.90815100	-2.83961600	-0.57233400
H	8.35921700	-1.69275900	-2.34708300
H	7.25282700	-3.82568400	1.23566500
H	8.74108400	-3.52066400	-0.72030200
C	4.16670000	1.84952500	1.13639200
H	4.21507800	2.76637900	0.54844600
H	3.39441200	1.97558700	1.89861100
H	5.13033500	1.68282100	1.62342900

Cartesian coordinates of the optimized geometries for **25** ([Au]$^+$ = JohnPhosAu$^+$) at B3LYP-D3BJ/6-31G(d),def2-TZVP+ECP level of theory (number of imaginary frequencies = 1):

C	1.22903500	1.41324700	-0.24047100
C	2.37022700	0.75171300	-0.35287300
C	1.30114400	2.84244200	0.14679400
Cl	2.41576600	-1.05345900	-1.02800700

C	2.12993700	3.73564600	-0.55347400
C	0.50224600	3.33653700	1.19282600
C	0.57191700	4.67823700	1.55860500
C	1.41112900	5.55603000	0.86664200
C	2.18008200	5.08273700	-0.19767100
H	2.81546400	5.76362200	-0.75630400
H	2.70564600	3.37236500	-1.39957100
H	-0.15995200	2.65628400	1.71647700
H	-0.04045900	5.04422400	2.37748300
Au	-0.62352000	0.51131900	-0.45933900
H	1.45172400	6.60458900	1.14486500
P	-2.76916300	-0.40251900	-0.73681000
C	-2.70470100	-1.68479500	-2.13079500
C	-3.95020000	1.05574700	-1.06871100
C	-1.59290900	-2.67400900	-1.72636700
H	-0.62432400	-2.17571800	-1.61967900
H	-1.49974100	-3.44297300	-2.50219500
H	-1.82898200	-3.17693200	-0.78304100
C	-4.00035000	-2.48202900	-2.34975100
H	-3.84847800	-3.16372000	-3.19506400
H	-4.85504700	-1.84727600	-2.59196300
H	-4.25197400	-3.09184400	-1.47906700
C	-2.30278900	-0.97151900	-3.43378000
H	-1.40230500	-0.36176800	-3.30359300
H	-3.10335700	-0.33078800	-3.81407300
H	-2.09255300	-1.72476000	-4.20202900
C	-3.20737900	2.08939000	-1.94209800
H	-2.30548900	2.46341300	-1.44855500
H	-3.87462100	2.94097700	-2.11952100
H	-2.91712700	1.68636900	-2.91512500
C	-5.27422500	0.70158200	-1.76391300
H	-5.11524100	0.29382100	-2.76559000
H	-5.86275200	1.61977900	-1.87671400
H	-5.88503100	0.00046700	-1.19216500
C	-4.23032100	1.69380400	0.30622700
H	-3.29957200	1.92668400	0.83677500
H	-4.83674500	1.04740800	0.94527000
H	-4.77284300	2.63441200	0.15699700
C	-3.42966800	-1.29273000	0.73348000
C	-4.76519100	-1.73954600	0.70371200
C	-2.67255500	-1.51565700	1.90700600
C	-5.35098400	-2.38228100	1.78920300
H	-5.36372200	-1.58316500	-0.18250200
C	-3.28135000	-2.16538100	2.99383800
C	-4.60327100	-2.59590400	2.94590100
H	-6.38390700	-2.71069600	1.72912000
H	-2.69051100	-2.33118800	3.88929300
H	-5.04392200	-3.09430600	3.80384700
C	-1.24551400	-1.12177200	2.11882600
C	-0.93636300	0.03936400	2.84334700
C	-0.20358300	-1.98057900	1.74209700
C	0.38683700	0.34307700	3.16748700
H	-1.74192600	0.69560600	3.16004400
C	1.11804900	-1.67830500	2.07146700
H	-0.43510800	-2.89399000	1.20367900
C	1.41753800	-0.51431600	2.77979900
H	0.61104700	1.24466300	3.72943400
H	1.91132900	-2.35956200	1.77877500
H	2.44613200	-0.28193500	3.04059900
C	4.27939600	-0.43934700	-0.33767100
C	3.77834900	0.75408100	-0.01260200
C	5.37387200	-1.34483700	-0.36705600
C	6.25504300	-1.35602100	-1.47109100
C	5.59207400	-2.22551200	0.71624900
C	7.34534400	-2.21703700	-1.47529700
H	6.07220800	-0.68359700	-2.30269100

C	6.68493300	-3.08195500	0.70128200
H	4.90357100	-2.21458600	1.55517300
C	7.55830400	-3.07750700	-0.39274200
H	8.02974400	-2.22192300	-2.31728000
H	6.86152100	-3.75264200	1.53581100
H	8.41051000	-3.75023600	-0.40149600
C	4.53209400	1.85638300	0.67129200
H	4.66185700	2.71393300	0.00630600
H	3.97032800	2.20149900	1.54457500
H	5.51492200	1.50205600	0.99145500

5. ^{1}H NMR and ^{13}C NMR Spectra of New Compounds

The field strengths and the solvents of the following spectra are given in the Chapter 2.

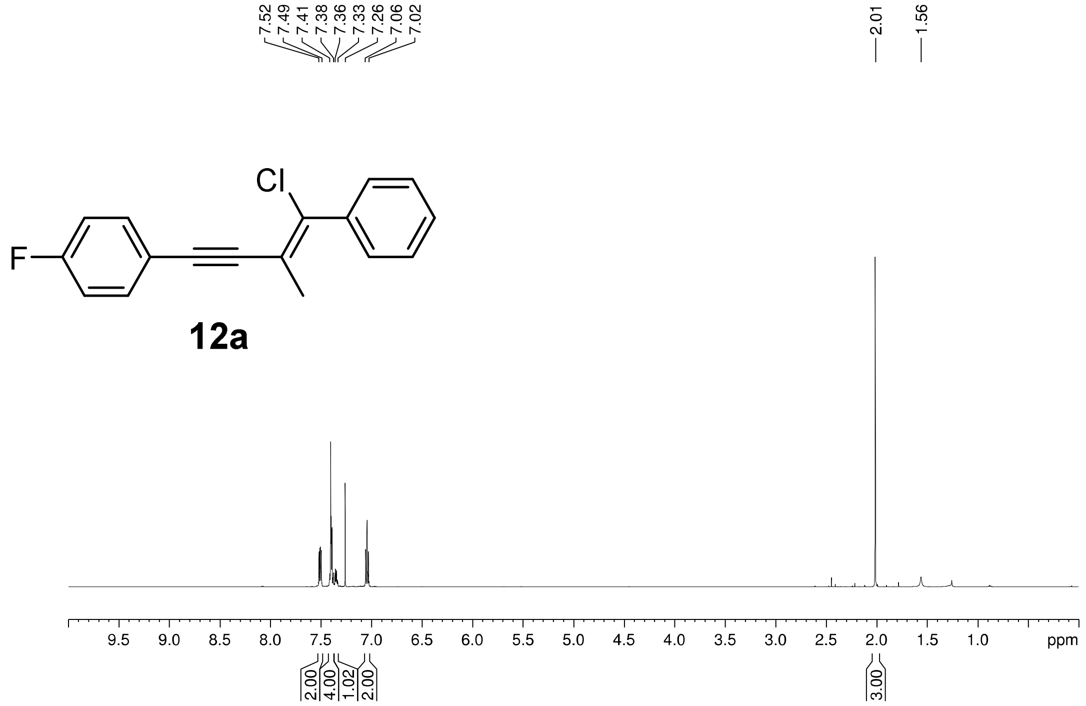

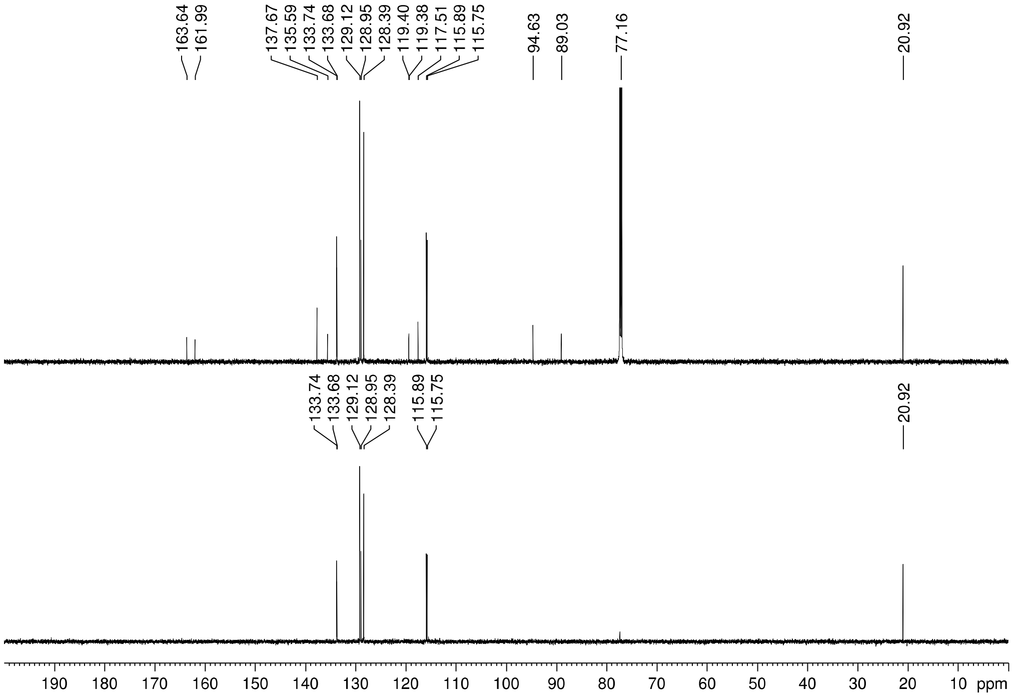

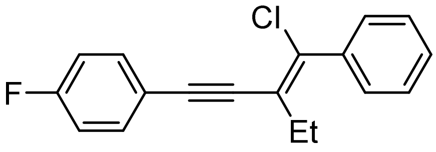
F
Cl
Et

12b

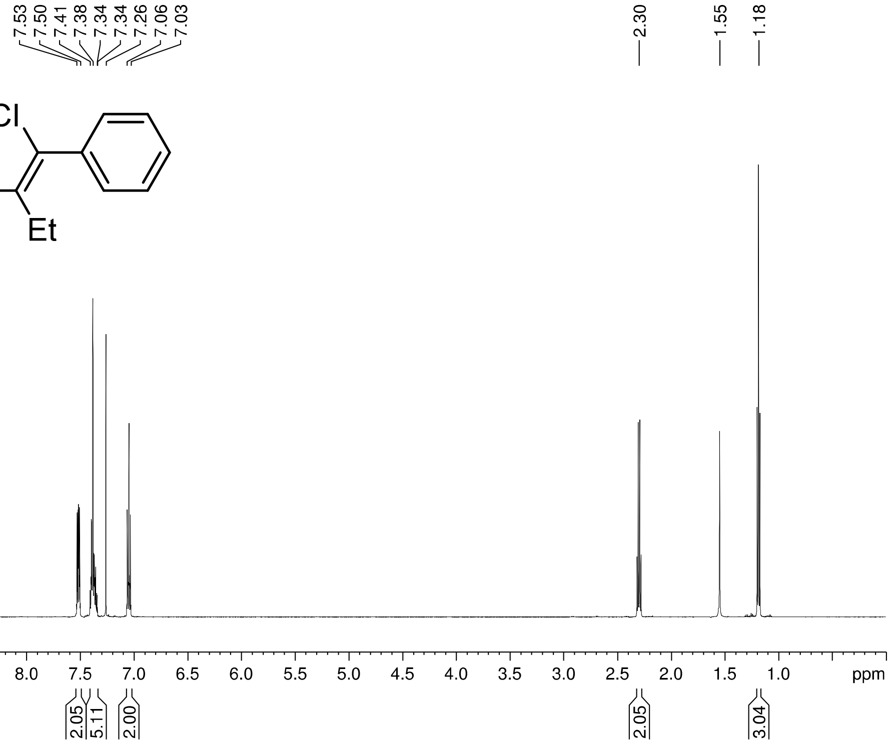
7.53 7.50 7.41 7.38 7.34 7.34 7.26 7.06 7.03
2.30
1.55
1.18
9.5 9.0 8.5 8.0 7.5 7.0 6.5 6.0 5.5 5.0 4.5 4.0 3.5 3.0 2.5 2.0 1.5 1.0 ppm
2.05 5.11 2.00 2.05 3.04

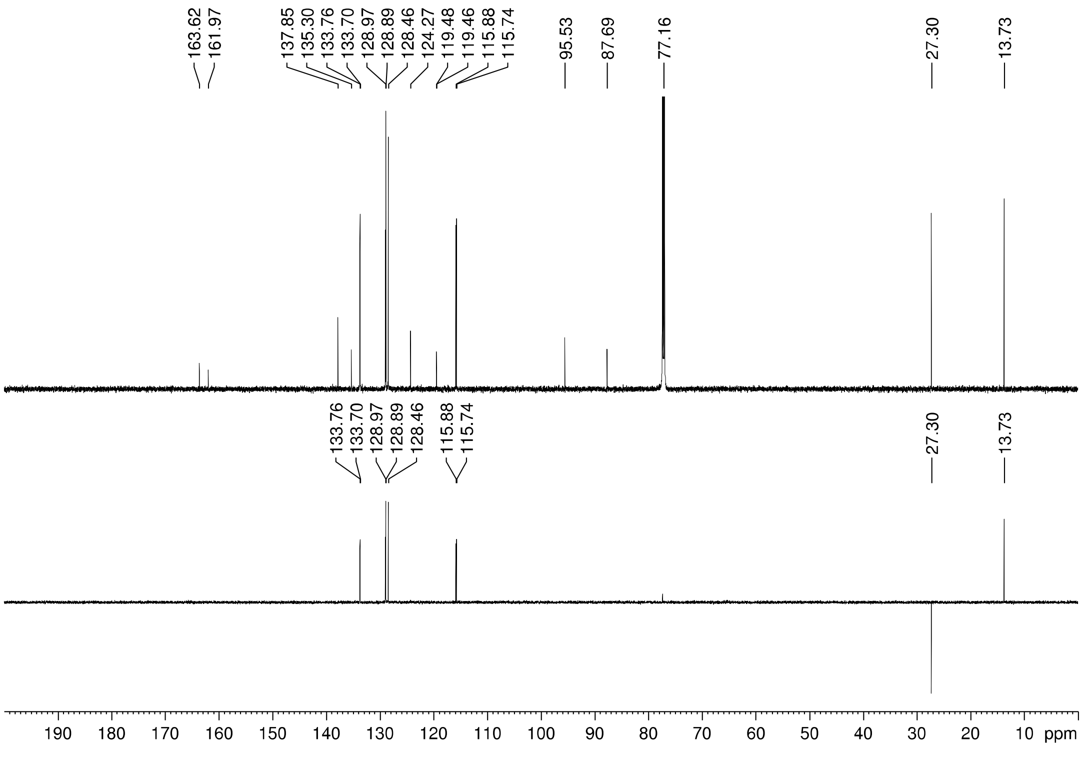
163.62 161.97
137.85 135.30 133.76 133.70 128.97 128.89 128.46 124.27 119.48 119.46 115.88 115.74
95.53
87.69
77.16
27.30
13.73
133.76 133.70 128.97 128.89 128.46 115.88 115.74
27.30
13.73
190 180 170 160 150 140 130 120 110 100 90 80 70 60 50 40 30 20 10 ppm

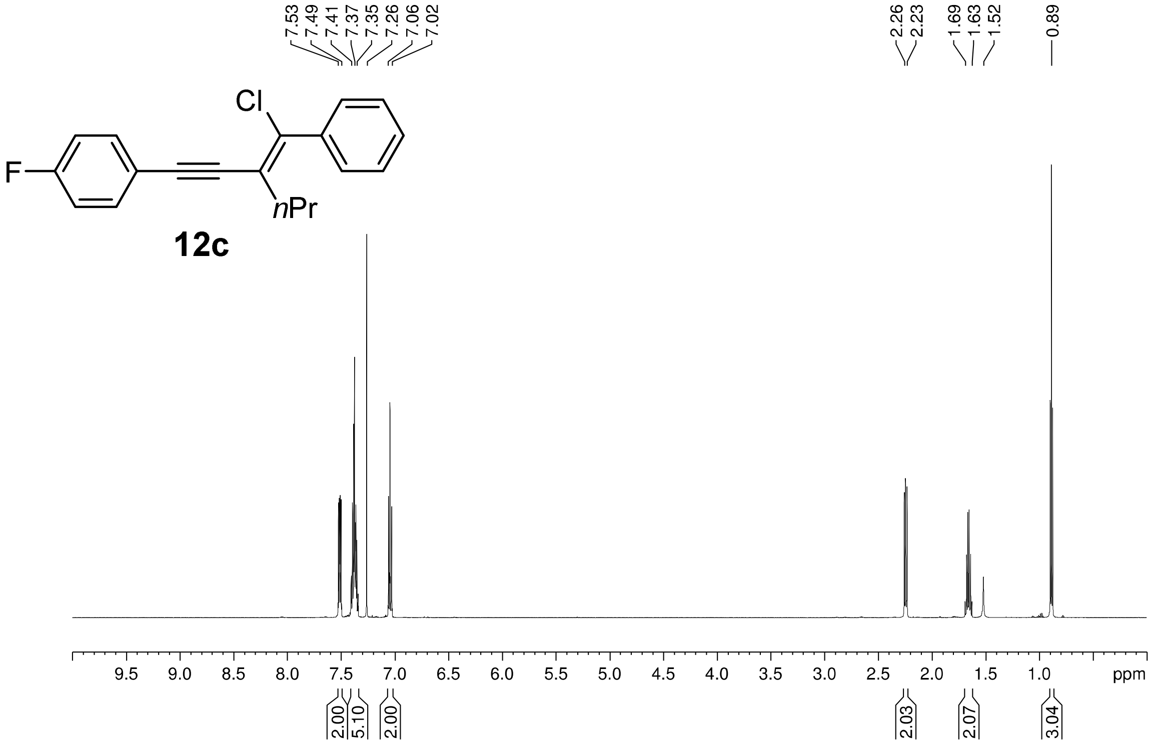
7.53
7.49
7.41
7.37
7.35
7.26
7.06
7.02
2.26
2.23
1.69
1.63
1.52
0.89
Cl
F
nPr
9.5 9.0 8.5 8.0 7.5 7.0 6.5 6.0 5.5 5.0 4.5 4.0 3.5 3.0 2.5 2.0 1.5 1.0 ppm
2.00
5.10
2.00
2.03
2.07
3.04

12c

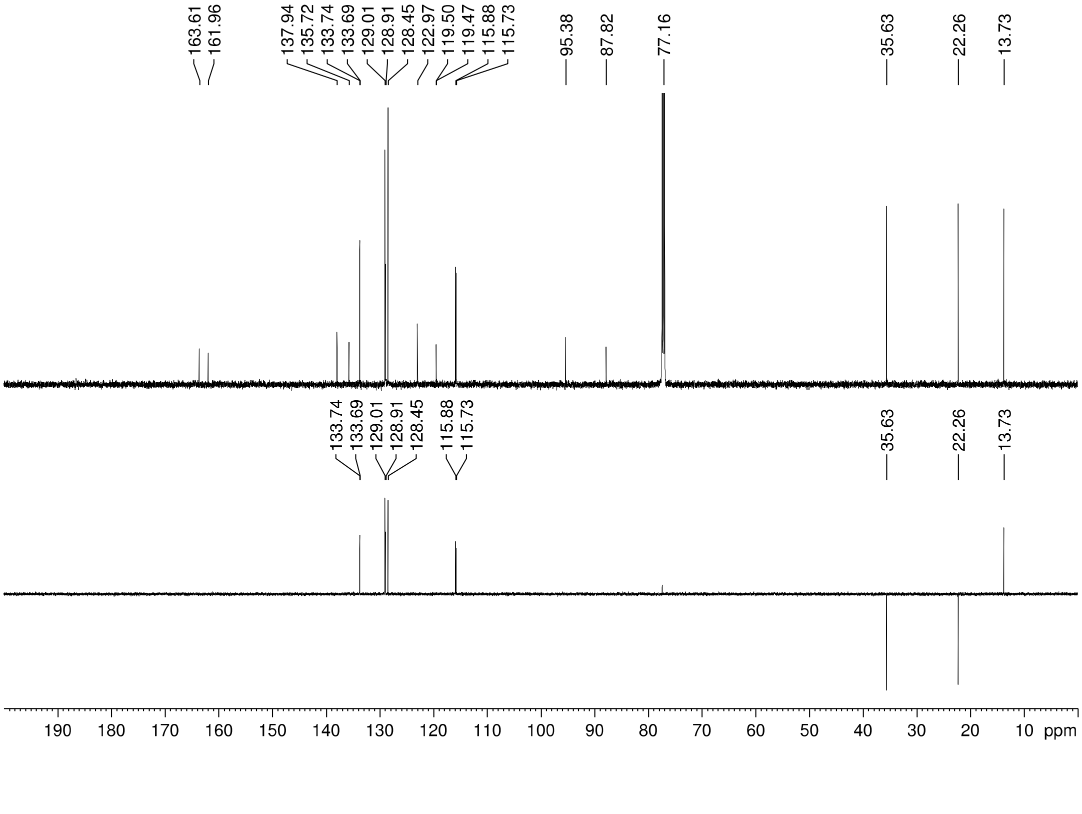
163.61
161.96
137.94
135.72
133.74
133.69
129.01
128.91
128.45
122.97
119.50
119.47
115.88
115.73
95.38
87.82
77.16
35.63
22.26
13.73
133.74
133.69
129.01
128.91
128.45
115.88
115.73
35.63
22.26
13.73
190 180 170 160 150 140 130 120 110 100 90 80 70 60 50 40 30 20 10 ppm

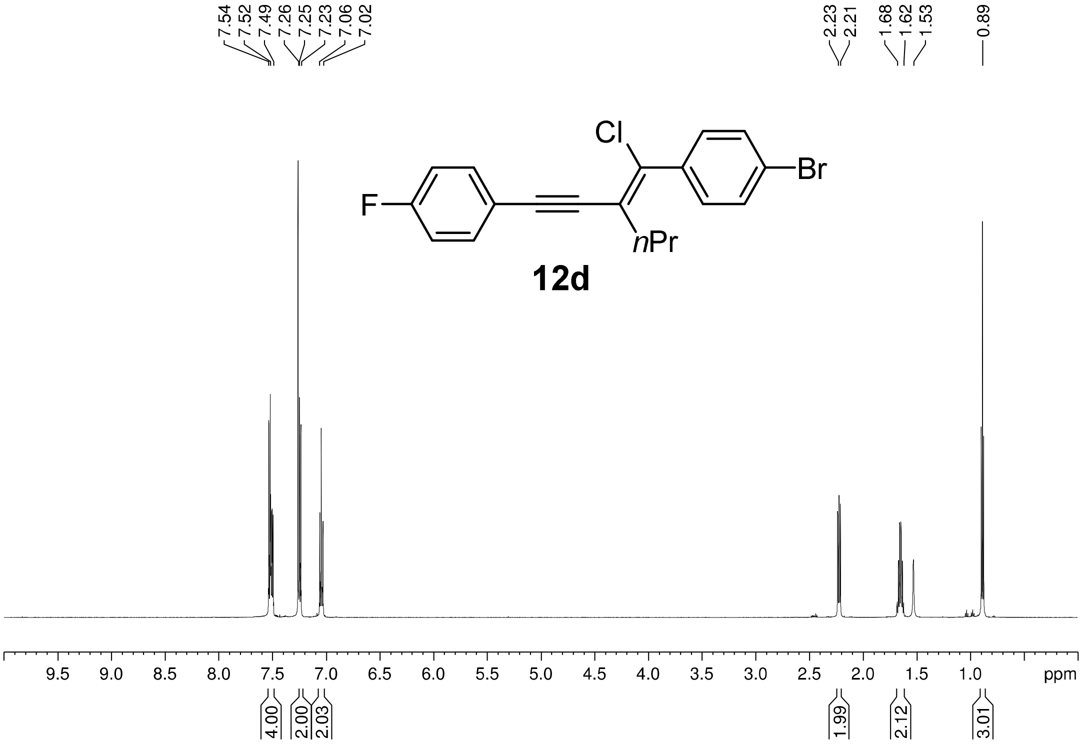
7.54 7.52 7.49 7.26 7.25 7.23 7.06 7.02
2.23 2.21 1.68 1.62 1.53 0.89
Cl
Br
F
nPr
12d
9.5 9.0 8.5 8.0 7.5 7.0 6.5 6.0 5.5 5.0 4.5 4.0 3.5 3.0 2.5 2.0 1.5 1.0 ppm
4.00 2.00 2.03 1.99 2.12 3.01

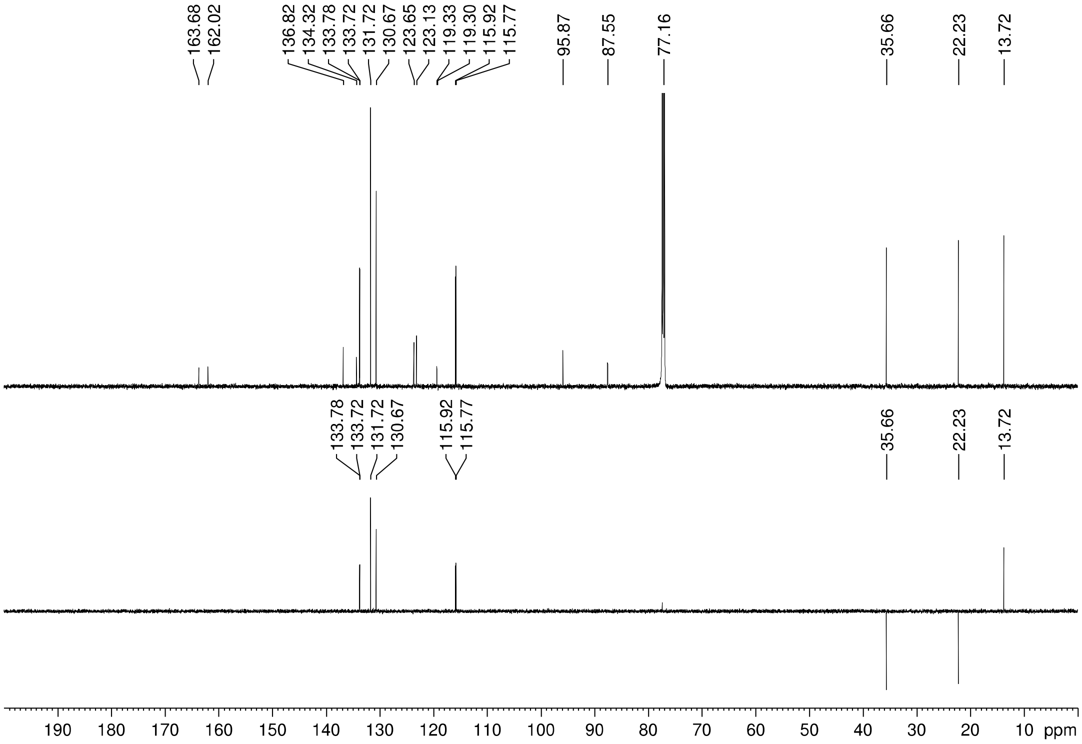
163.68 162.02 136.82 134.32 133.78 133.72 131.72 130.67 123.65 123.13 119.33 119.30 115.92 115.77 95.87 87.55 77.16 35.66 22.23 13.72
133.78 133.72 131.72 130.67 115.92 115.77 35.66 22.23 13.72
190 180 170 160 150 140 130 120 110 100 90 80 70 60 50 40 30 20 10 ppm

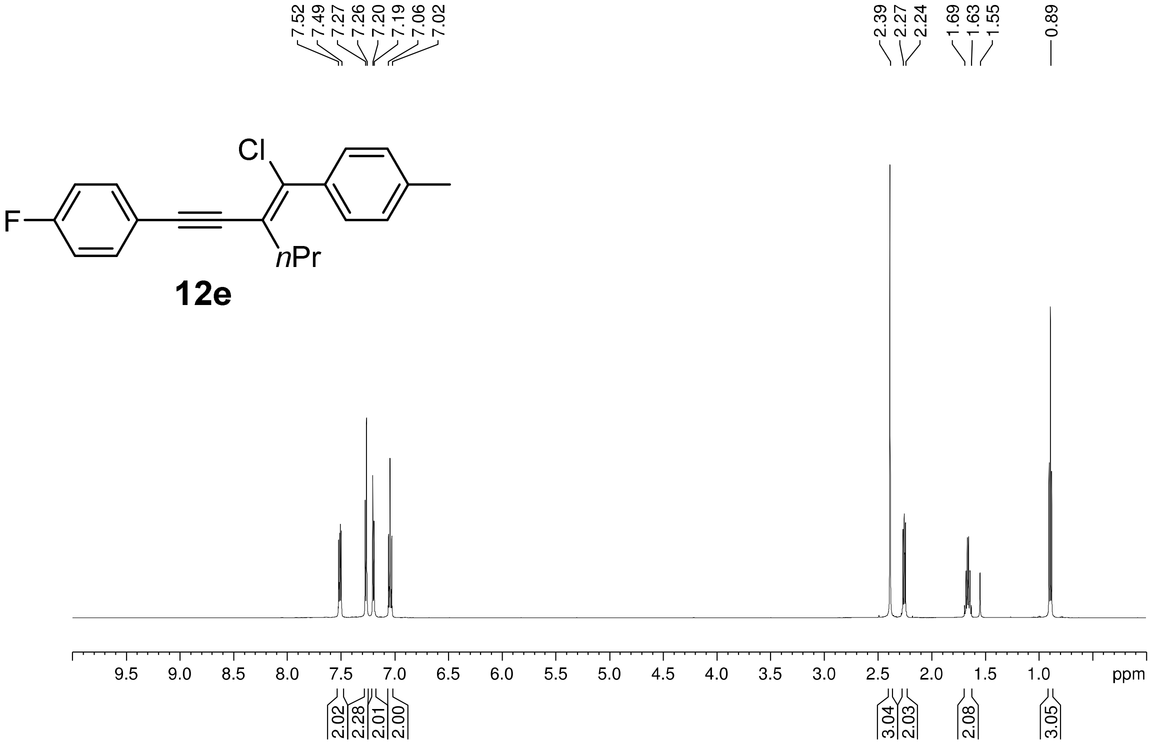
Cl
F
nPr
7.52
7.49
7.27
7.26
7.20
7.19
7.06
7.02
2.39
2.27
2.24
1.69
1.63
1.55
0.89
9.5 9.0 8.5 8.0 7.5 7.0 6.5 6.0 5.5 5.0 4.5 4.0 3.5 3.0 2.5 2.0 1.5 1.0 ppm
2.02
2.28
2.01
2.00
3.04
2.03
2.08
3.05

12e

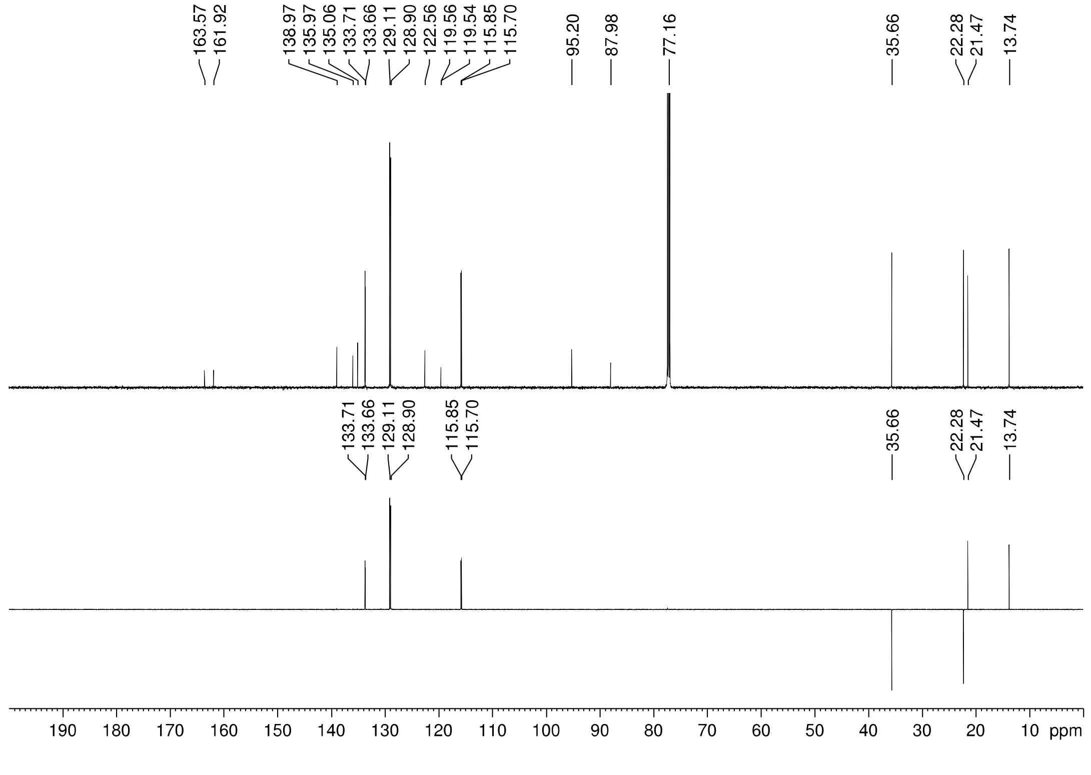
163.57
161.92
138.97
135.97
135.06
133.71
133.66
129.11
128.90
122.56
119.56
119.54
115.85
115.70
95.20
87.98
77.16
35.66
22.28
21.47
13.74
133.71
133.66
129.11
128.90
115.85
115.70
35.66
22.28
21.47
13.74
190 180 170 160 150 140 130 120 110 100 90 80 70 60 50 40 30 20 10 ppm

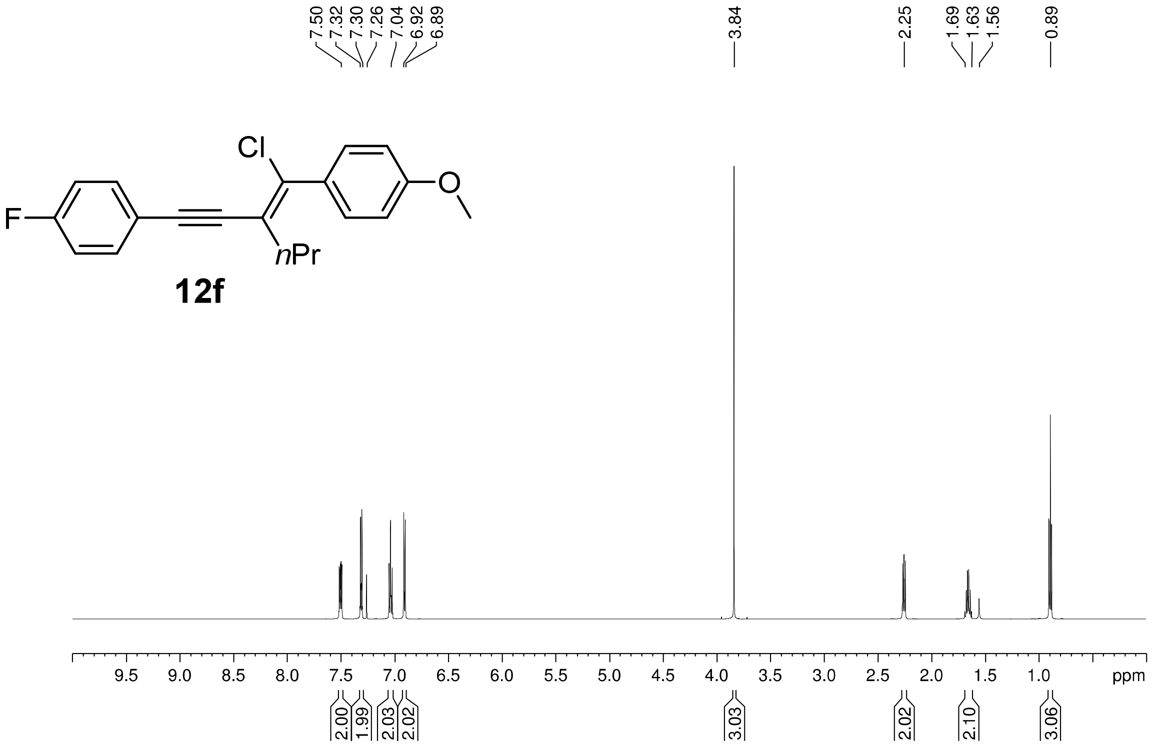
Cl
F
O
nPr
12f
7.50
7.32
7.30
7.26
7.04
6.92
6.89
3.84
2.25
1.69
1.63
1.56
0.89
9.5 9.0 8.5 8.0 7.5 7.0 6.5 6.0 5.5 5.0 4.5 4.0 3.5 3.0 2.5 2.0 1.5 1.0 ppm
2.00
1.99
2.03
2.02
3.03
2.02
2.10
3.06

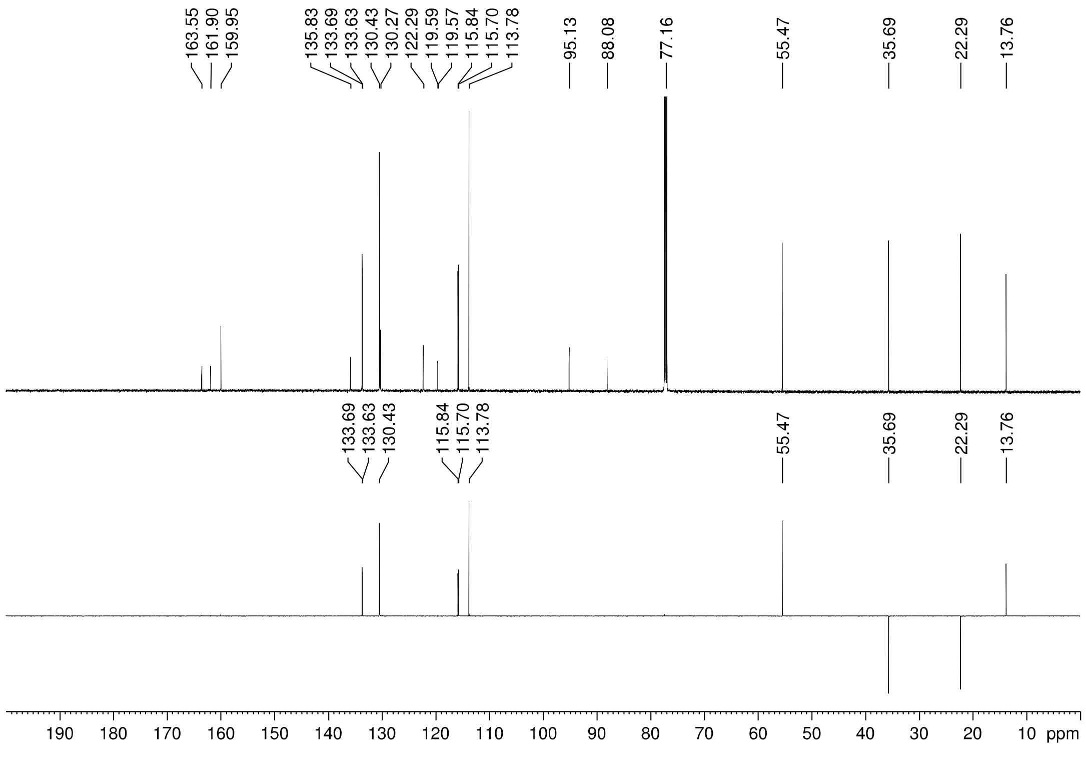
163.55
161.90
159.95
135.83
133.69
133.63
130.43
130.27
122.29
119.59
119.57
115.84
115.70
113.78
95.13
88.08
77.16
55.47
35.69
22.29
13.76
133.69
133.63
130.43
115.84
115.70
113.78
55.47
35.69
22.29
13.76
190 180 170 160 150 140 130 120 110 100 90 80 70 60 50 40 30 20 10 ppm

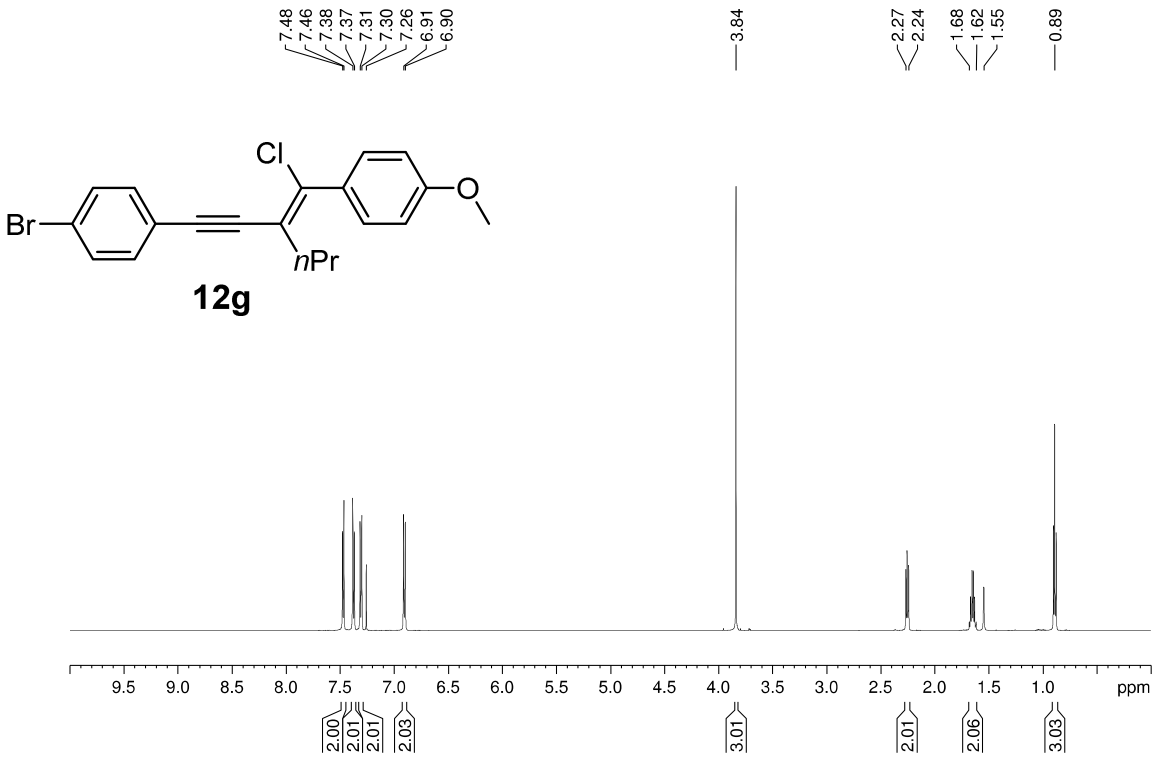

12g

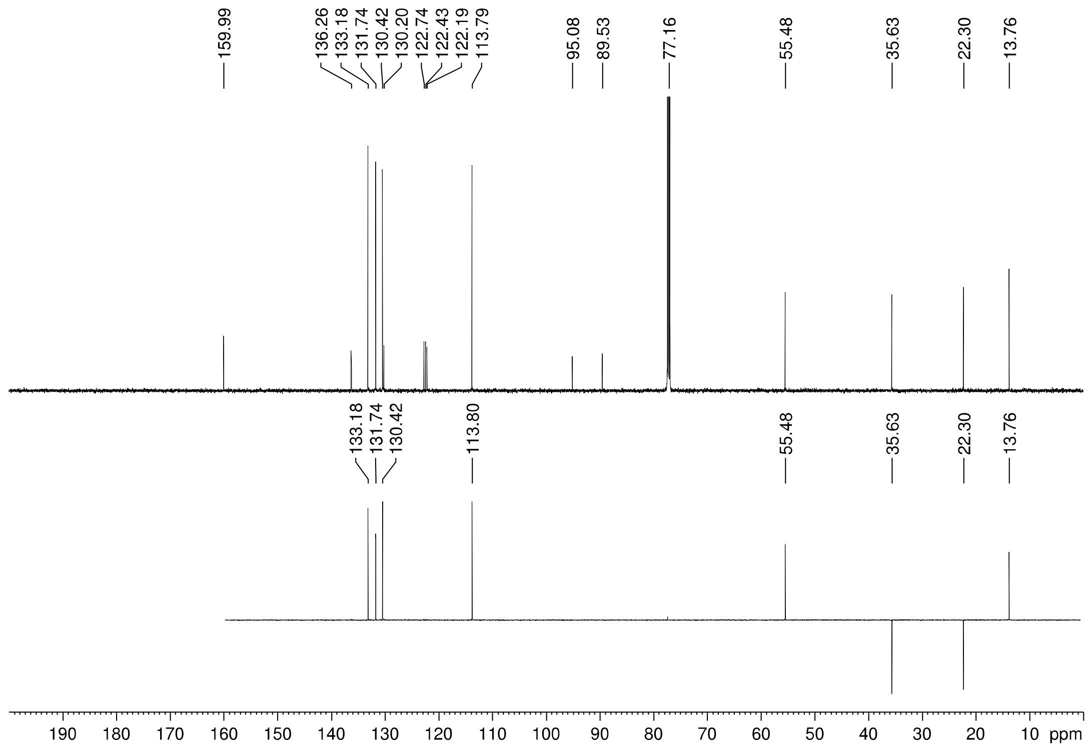

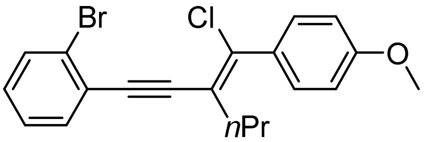

12h

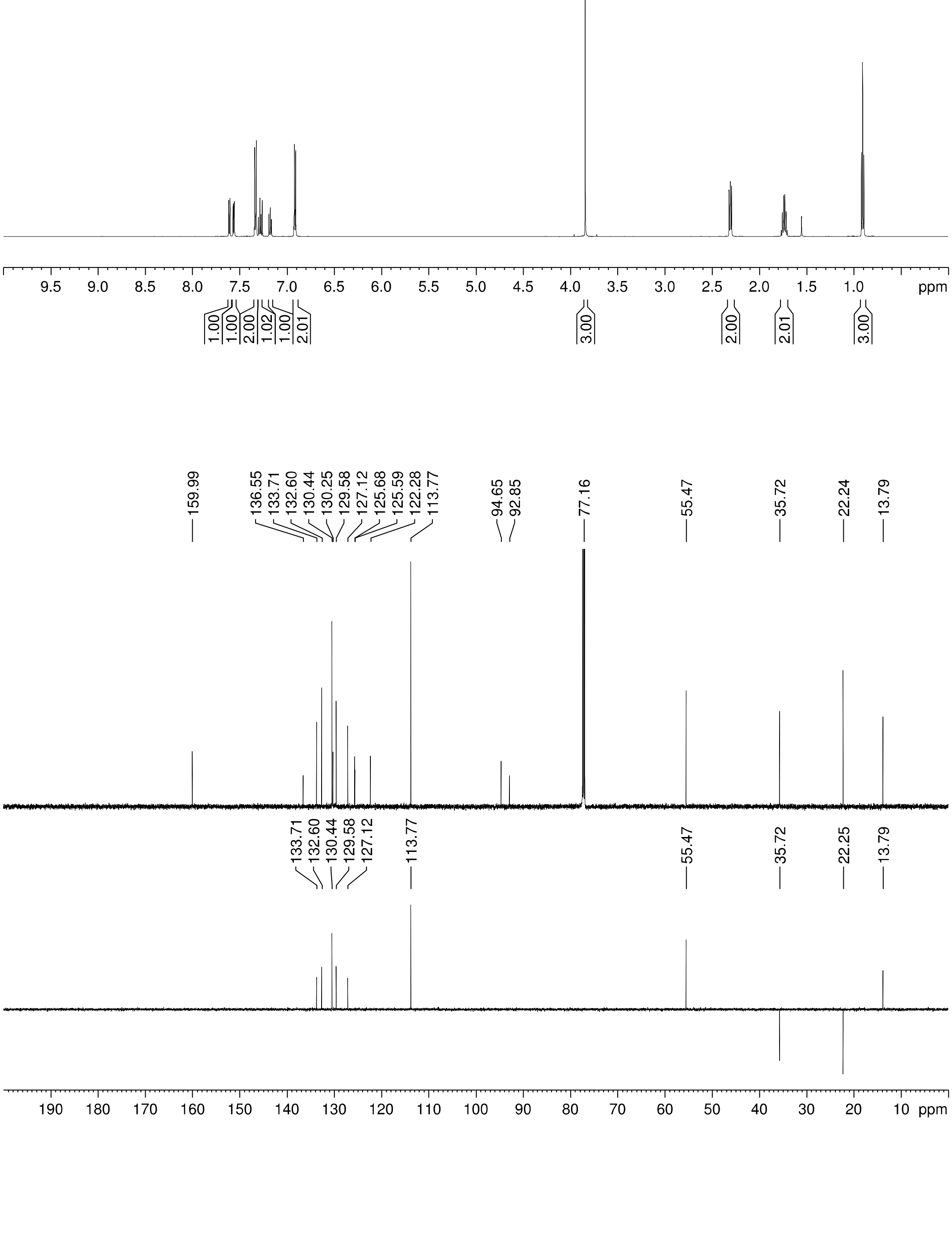

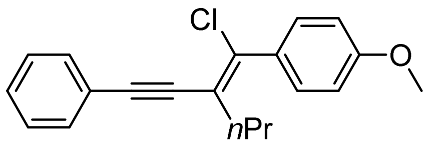
Cl
nPr

12i

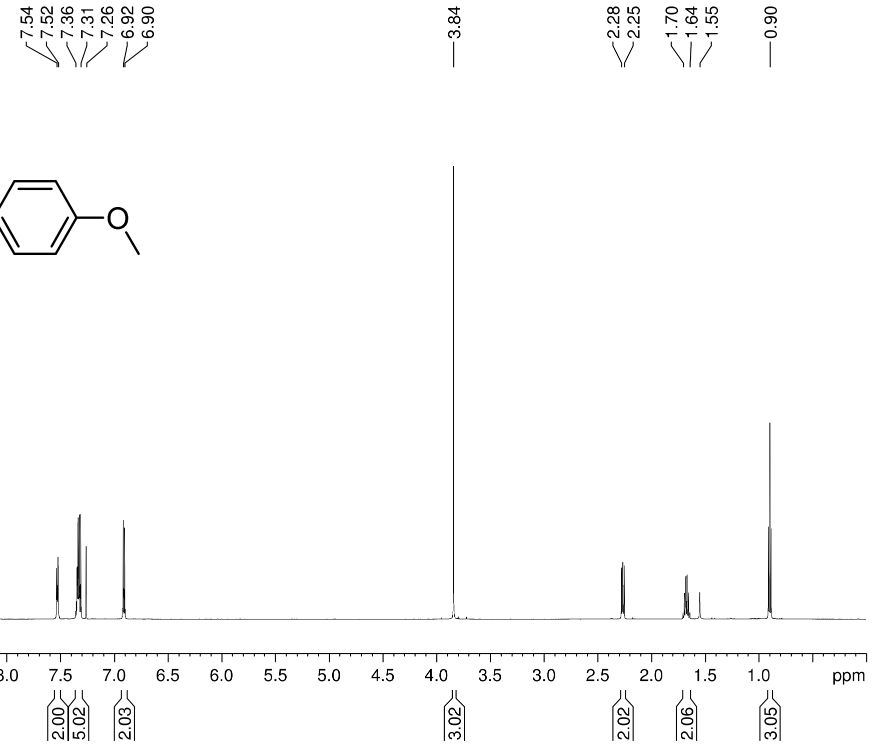
7.54
7.52
7.36
7.31
7.26
6.92
6.90
3.84
2.28
2.25
1.70
1.64
1.55
0.90
9.5 9.0 8.5 8.0 7.5 7.0 6.5 6.0 5.5 5.0 4.5 4.0 3.5 3.0 2.5 2.0 1.5 1.0 ppm
2.00
5.02
2.03
3.02
2.02
2.06
3.05

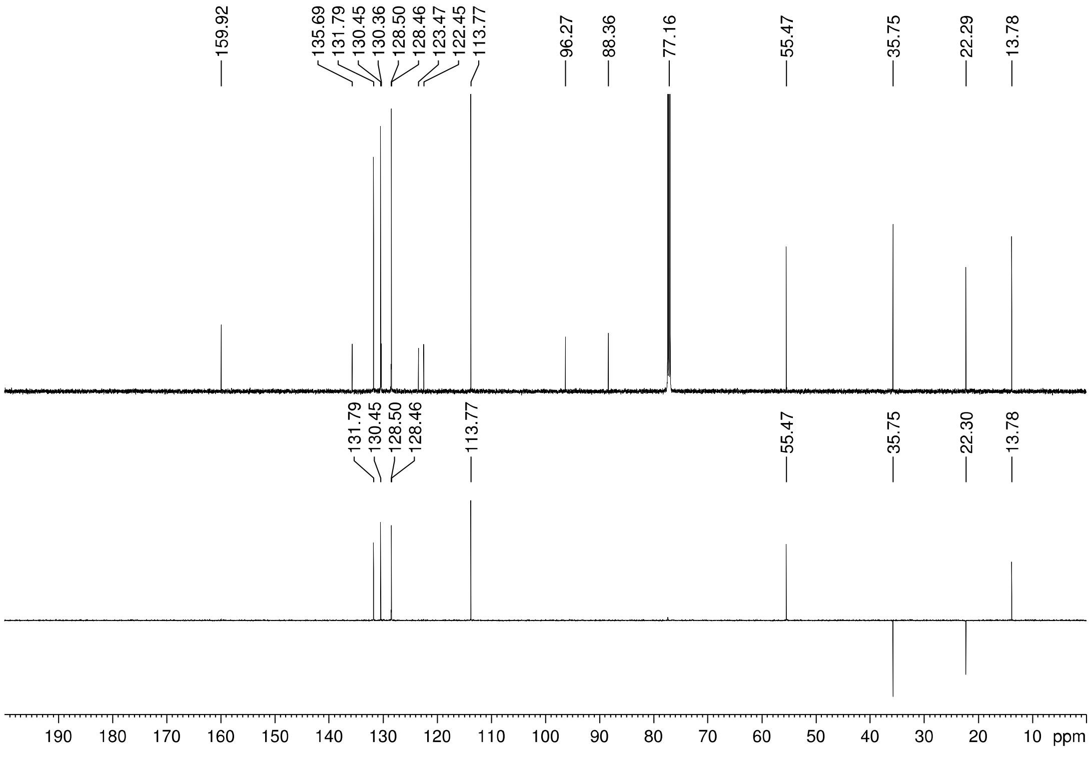
159.92
135.69
131.79
130.45
130.36
128.50
128.46
123.47
122.45
113.77
96.27
88.36
77.16
55.47
35.75
22.29
13.78
131.79
130.45
128.50
128.46
113.77
55.47
35.75
22.30
13.78
190 180 170 160 150 140 130 120 110 100 90 80 70 60 50 40 30 20 10 ppm

12j

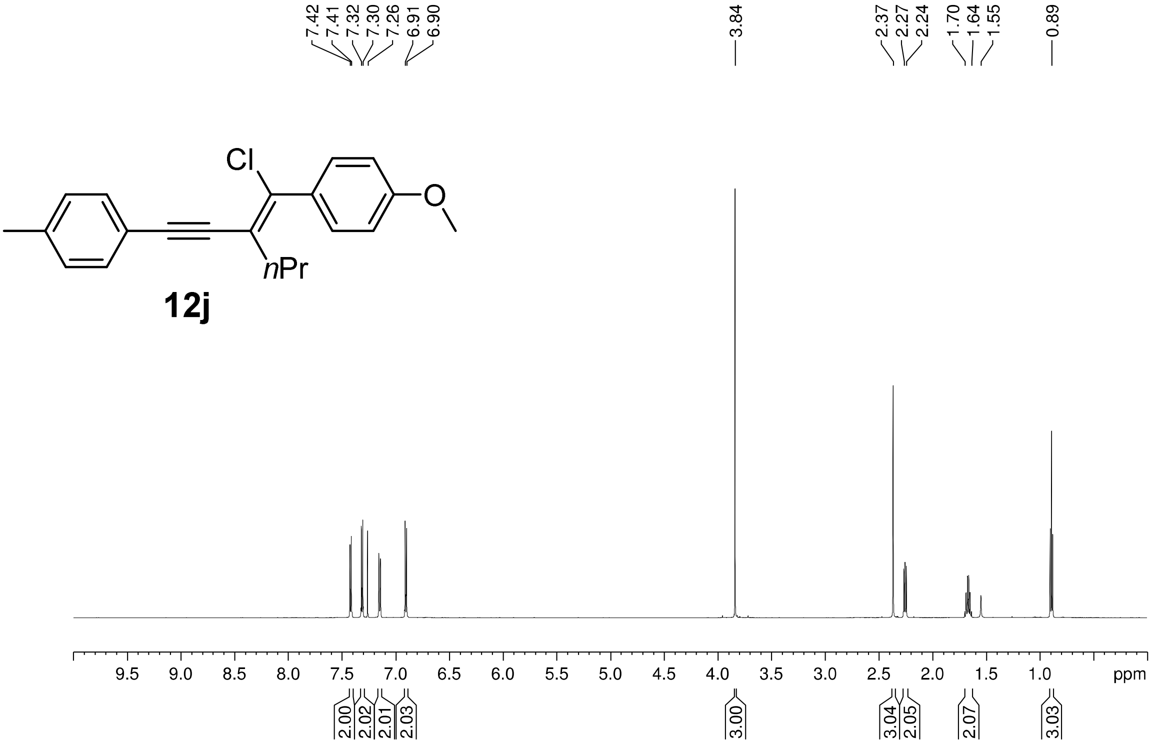
Cl
O
nPr
7.42
7.41
7.32
7.30
7.26
6.91
6.90
3.84
2.37
2.27
2.24
1.70
1.64
1.55
0.89
2.00
2.02
2.01
2.03
3.00
3.04
2.05
2.07
3.03
ppm

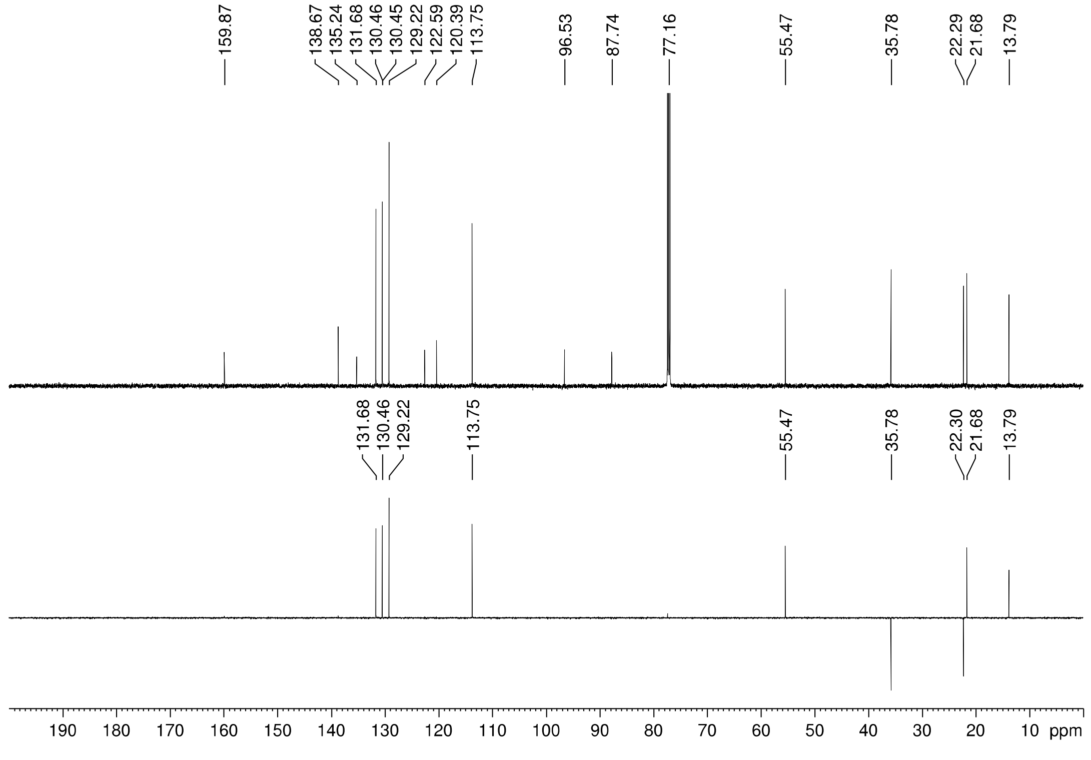
159.87
138.67
135.24
131.68
130.46
130.45
129.22
122.59
120.39
113.75
96.53
87.74
77.16
55.47
35.78
22.29
21.68
13.79
131.68
130.46
129.22
113.75
55.47
35.78
22.30
21.68
13.79
ppm

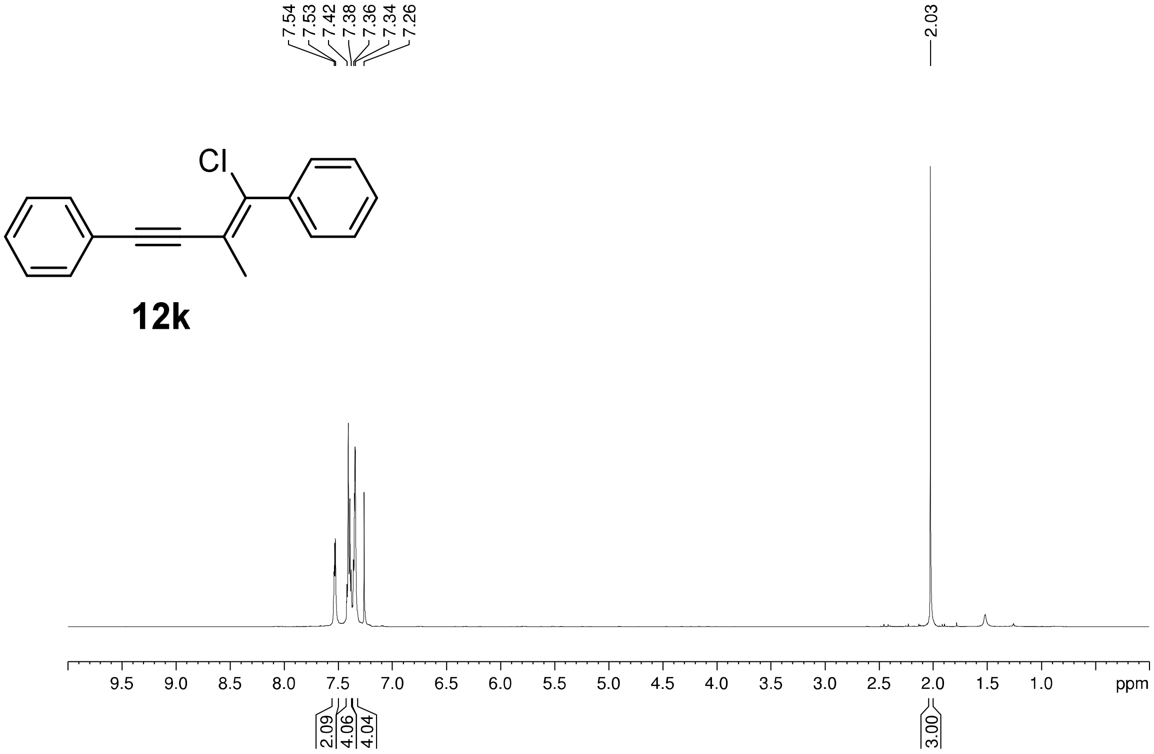
Cl
12k
7.54
7.53
7.42
7.38
7.36
7.34
7.26
2.03
9.5 9.0 8.5 8.0 7.5 7.0 6.5 6.0 5.5 5.0 4.5 4.0 3.5 3.0 2.5 2.0 1.5 1.0 ppm
2.09
4.06
4.04
3.00

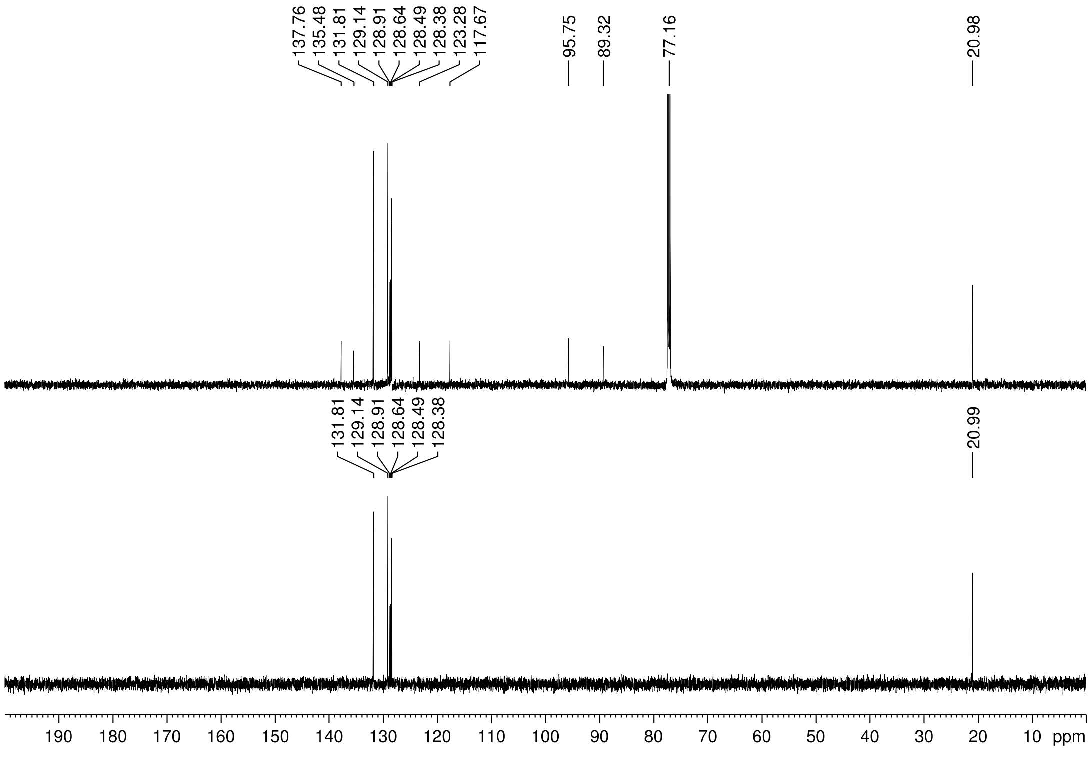
137.76
135.48
131.81
129.14
128.91
128.64
128.49
128.38
123.28
117.67
95.75
89.32
77.16
20.98
131.81
129.14
128.91
128.64
128.49
128.38
20.99
190 180 170 160 150 140 130 120 110 100 90 80 70 60 50 40 30 20 10 ppm

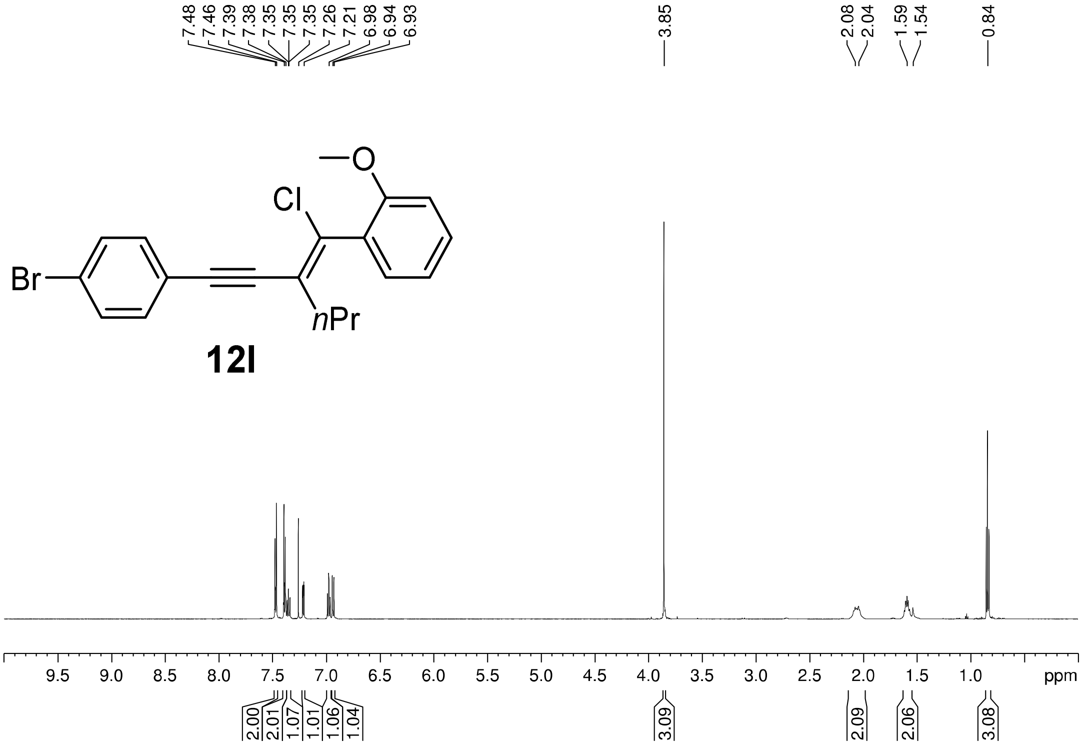
7.48 7.46 7.39 7.38 7.35 7.35 7.35 7.26 7.21 6.98 6.94 6.93
3.85
2.08 2.04 1.59 1.54
0.84
Br
Cl
O
nPr
12l
9.5 9.0 8.5 8.0 7.5 7.0 6.5 6.0 5.5 5.0 4.5 4.0 3.5 3.0 2.5 2.0 1.5 1.0 ppm
2.00 2.01 1.07 1.01 1.06 1.04
3.09
2.09 2.06
3.08

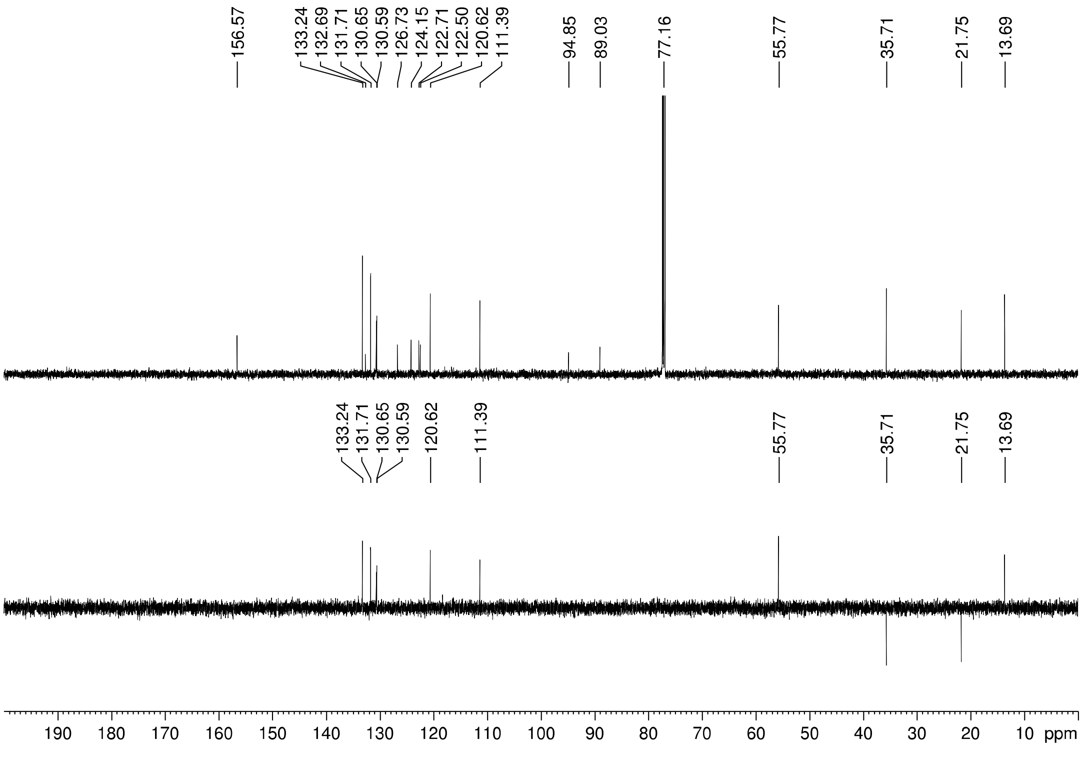
156.57
133.24 132.69 131.71 130.65 130.59 126.73 124.15 122.71 122.50 120.62 111.39
94.85 89.03 77.16
55.77
35.71
21.75 13.69
133.24 131.71 130.65 130.59 120.62 111.39
55.77
35.71
21.75 13.69
190 180 170 160 150 140 130 120 110 100 90 80 70 60 50 40 30 20 10 ppm

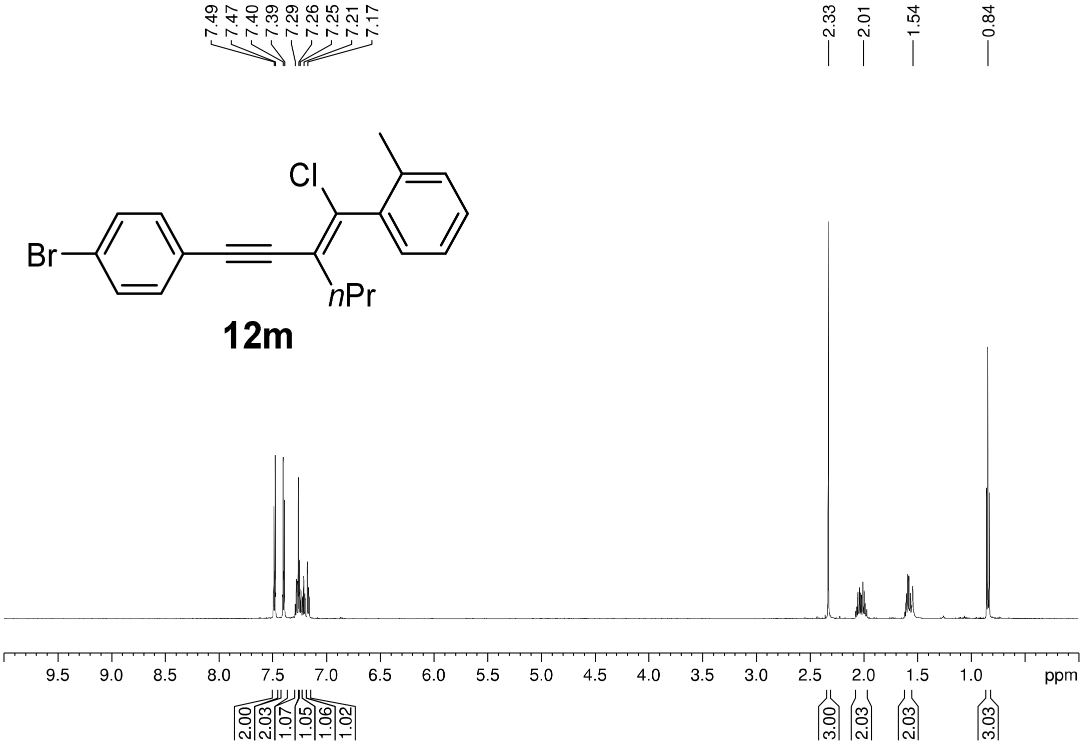
7.49 7.47 7.40 7.39 7.29 7.26 7.25 7.21 7.17
2.33 2.01 1.54 0.84
Cl
Br
nPr
12m
9.5 9.0 8.5 8.0 7.5 7.0 6.5 6.0 5.5 5.0 4.5 4.0 3.5 3.0 2.5 2.0 1.5 1.0 ppm
2.00 2.03 1.07 1.05 1.06 1.02 3.00 2.03 2.03 3.03

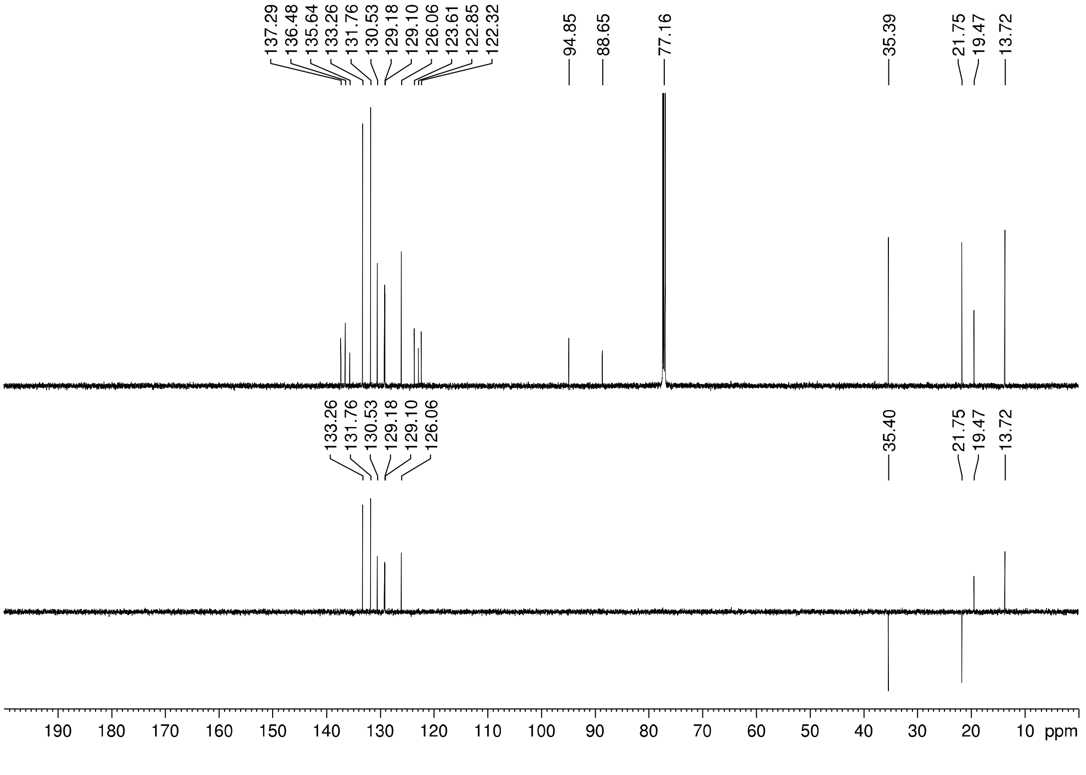
137.29 136.48 135.64 133.26 131.76 130.53 129.18 129.10 126.06 123.61 122.85 122.32
94.85 88.65 77.16 35.39 21.75 19.47 13.72
133.26 131.76 130.53 129.18 129.10 126.06
35.40 21.75 19.47 13.72
190 180 170 160 150 140 130 120 110 100 90 80 70 60 50 40 30 20 10 ppm

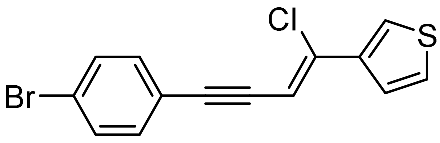
Cl
S
Br

12n

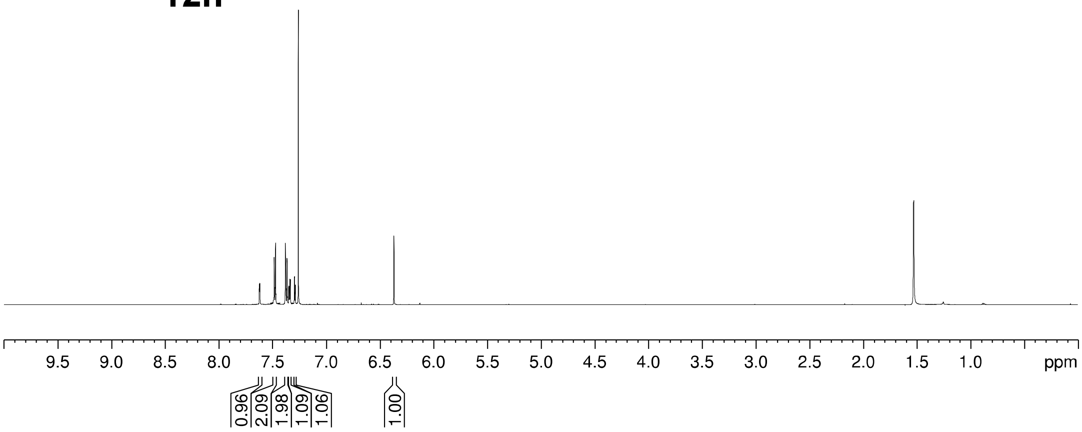
7.62
7.48
7.47
7.38
7.36
7.35
7.33
7.29
7.29
7.26
6.37
1.53
9.5 9.0 8.5 8.0 7.5 7.0 6.5 6.0 5.5 5.0 4.5 4.0 3.5 3.0 2.5 2.0 1.5 1.0 ppm
0.96
2.09
1.98
1.09
1.06
1.00

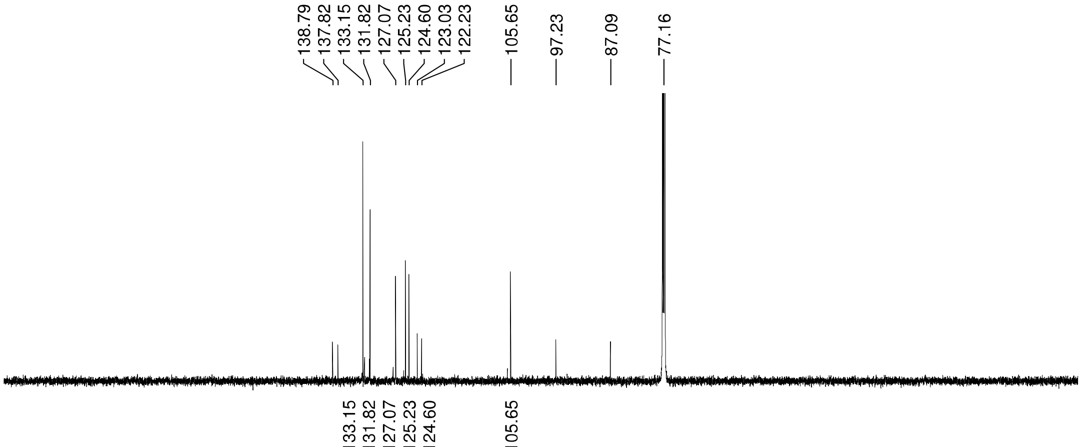
138.79
137.82
133.15
131.82
127.07
125.23
124.60
123.03
122.23
105.65
97.23
87.09
77.16

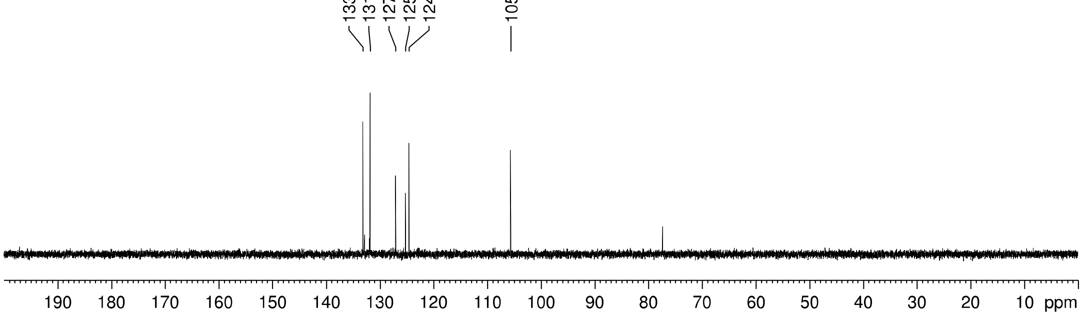
133.15
131.82
127.07
125.23
124.60
105.65
190 180 170 160 150 140 130 120 110 100 90 80 70 60 50 40 30 20 10 ppm

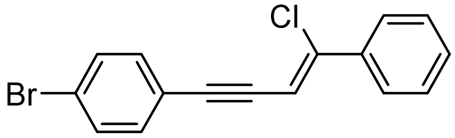
Br
Cl

12o

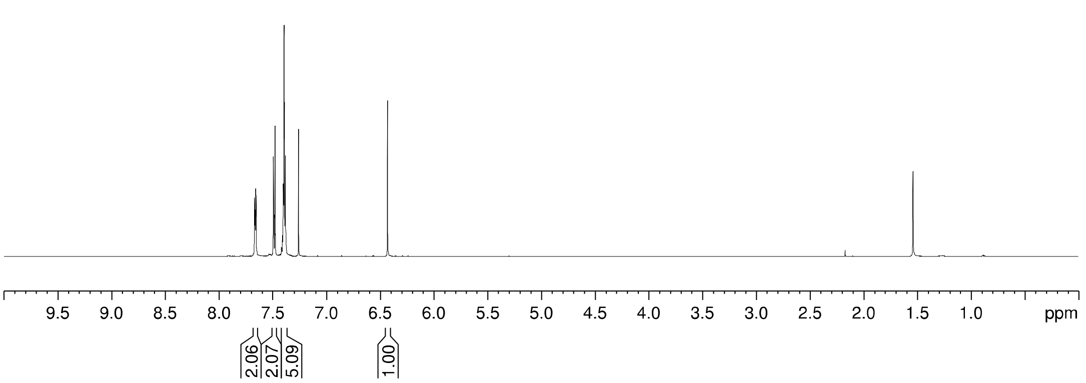
7.67
7.65
7.49
7.48
7.39
7.26
6.43
1.54
9.5 9.0 8.5 8.0 7.5 7.0 6.5 6.0 5.5 5.0 4.5 4.0 3.5 3.0 2.5 2.0 1.5 1.0 ppm
2.06
2.07
5.09
1.00

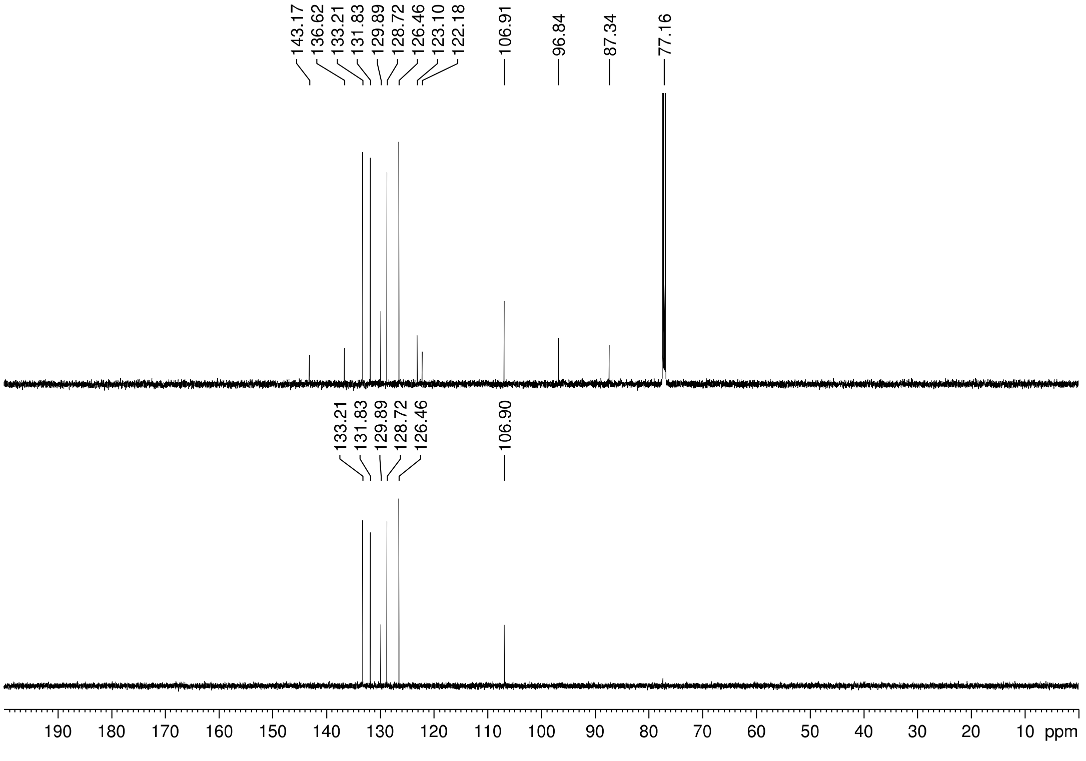
143.17
136.62
133.21
131.83
129.89
128.72
126.46
123.10
122.18
106.91
96.84
87.34
77.16
133.21
131.83
129.89
128.72
126.46
106.90
190 180 170 160 150 140 130 120 110 100 90 80 70 60 50 40 30 20 10 ppm

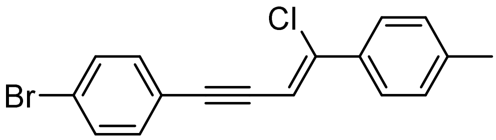
Cl
Br

12p

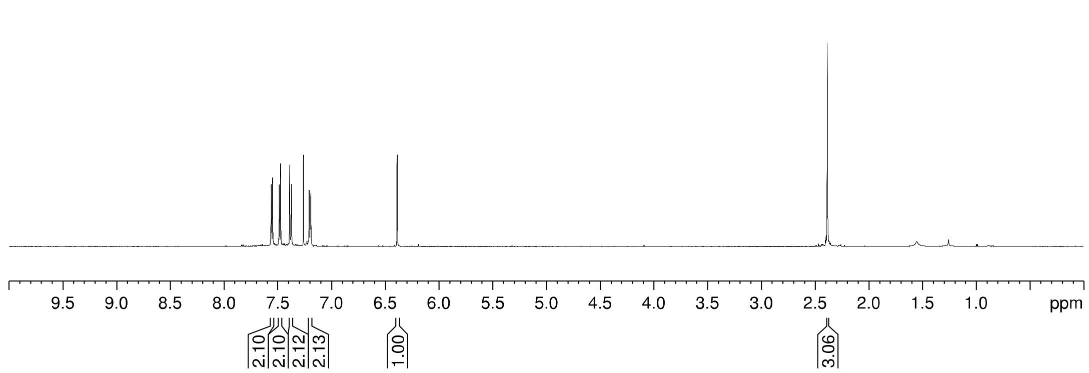
7.56
7.55
7.49
7.47
7.39
7.37
7.26
7.20
7.19
6.39
2.38
9.5 9.0 8.5 8.0 7.5 7.0 6.5 6.0 5.5 5.0 4.5 4.0 3.5 3.0 2.5 2.0 1.5 1.0 ppm
2.10
2.10
2.12
2.13
1.00
3.06

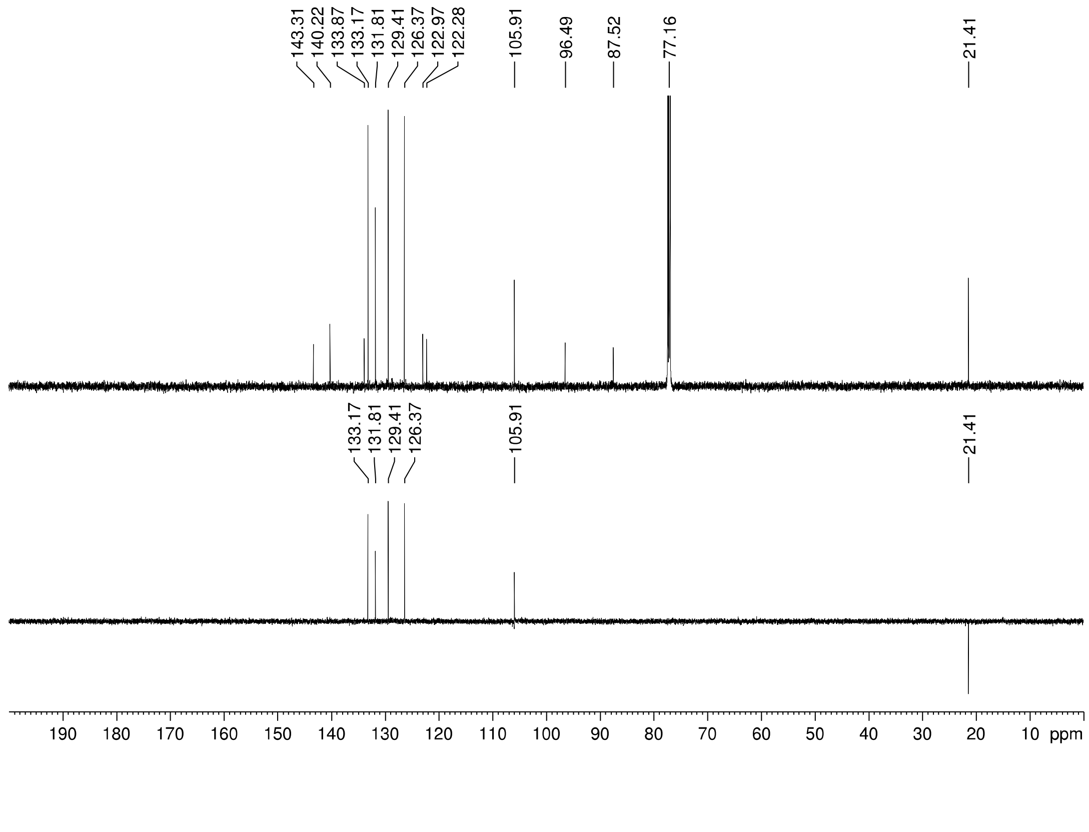
143.31
140.22
133.87
133.17
131.81
129.41
126.37
122.97
122.28
105.91
96.49
87.52
77.16
21.41
133.17
131.81
129.41
126.37
105.91
21.41
190 180 170 160 150 140 130 120 110 100 90 80 70 60 50 40 30 20 10 ppm

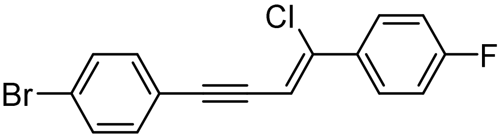

12q

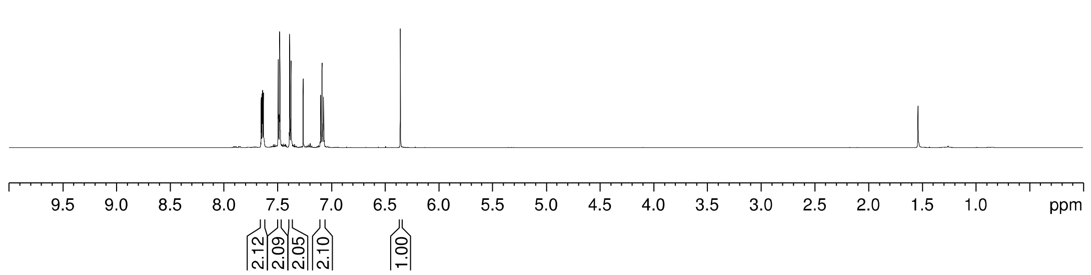
7.65
7.63
7.49
7.48
7.39
7.37
7.26
7.08
6.36
1.54
9.5 9.0 8.5 8.0 7.5 7.0 6.5 6.0 5.5 5.0 4.5 4.0 3.5 3.0 2.5 2.0 1.5 1.0 ppm
2.12
2.09
2.05
2.10
1.00

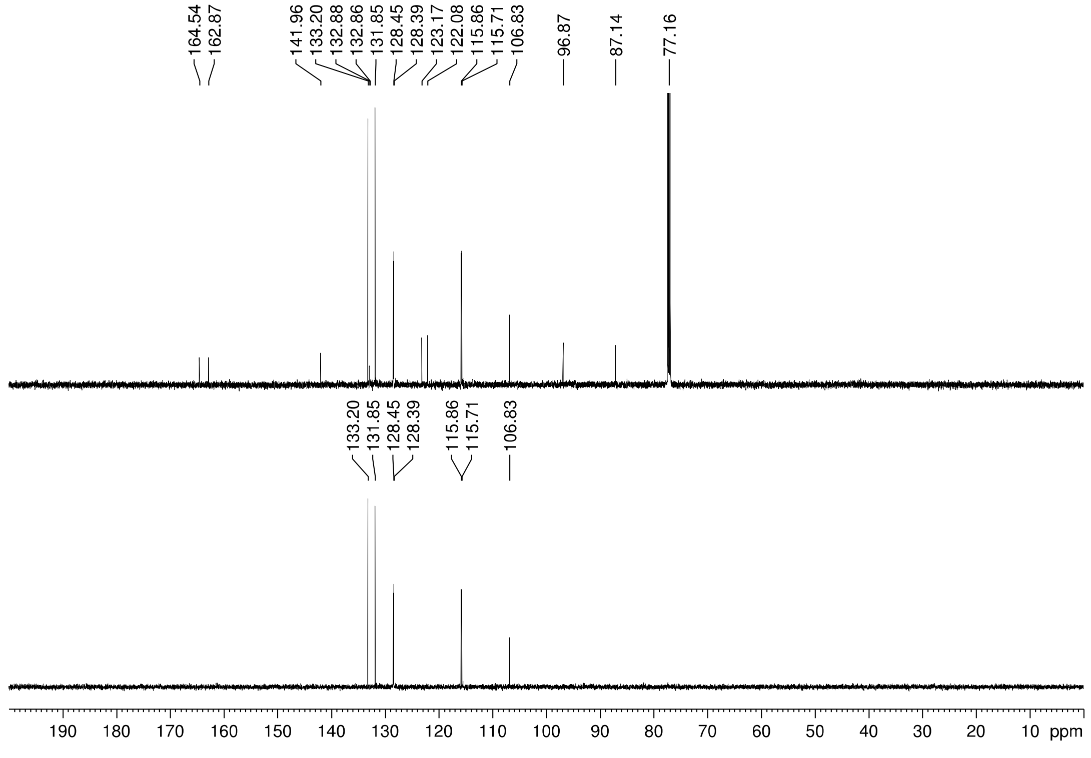
164.54
162.87
141.96
133.20
132.88
132.86
131.85
128.45
128.39
123.17
122.08
115.86
115.71
106.83
96.87
87.14
77.16
133.20
131.85
128.45
128.39
115.86
115.71
106.83
190 180 170 160 150 140 130 120 110 100 90 80 70 60 50 40 30 20 10 ppm

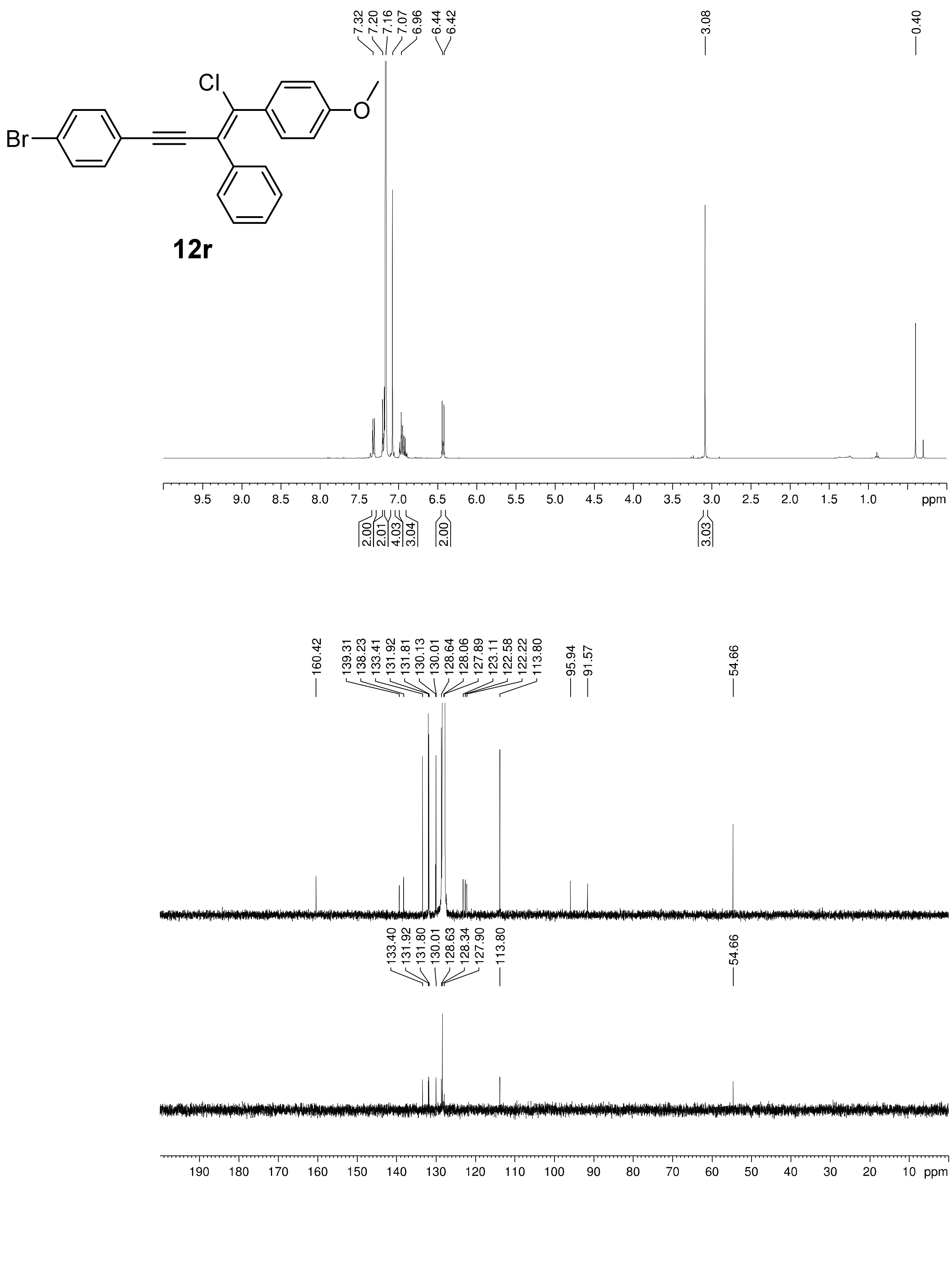
Br
Cl
O

12r

7.32
7.20
7.16
7.07
6.96
6.44
6.42
3.08
0.40
9.5
9.0
8.5
8.0
7.5
7.0
6.5
6.0
5.5
5.0
4.5
4.0
3.5
3.0
2.5
2.0
1.5
1.0
ppm
2.00
2.01
4.03
3.04
2.00
3.03
160.42
139.31
138.23
133.41
131.92
131.81
130.13
130.01
128.64
128.06
127.89
123.11
122.58
122.22
113.80
95.94
91.57
54.66
133.40
131.92
131.80
130.01
128.63
128.34
127.90
113.80
54.66
190
180
170
160
150
140
130
120
110
100
90
80
70
60
50
40
30
20
10
ppm

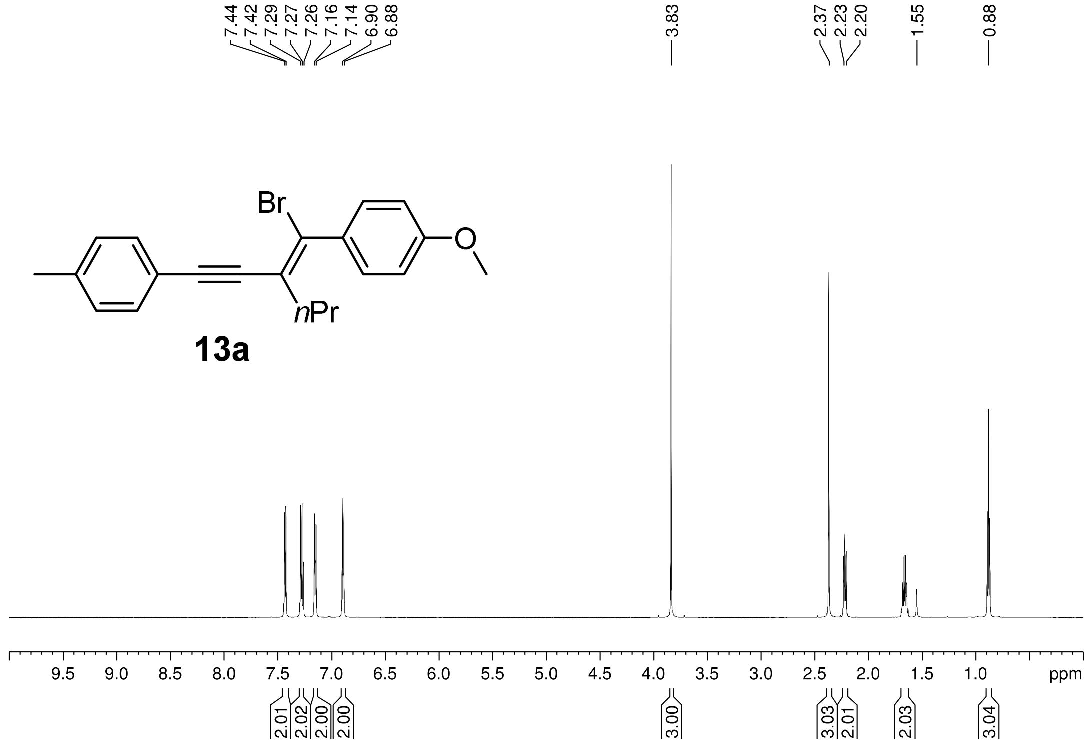
Br
nPr
7.44
7.42
7.29
7.27
7.26
7.16
7.14
6.90
6.88
3.83
2.37
2.23
2.20
1.55
0.88
9.5 9.0 8.5 8.0 7.5 7.0 6.5 6.0 5.5 5.0 4.5 4.0 3.5 3.0 2.5 2.0 1.5 1.0 ppm
2.01 2.02 2.00 2.00 3.00 3.03 2.01 2.03 3.04

13a

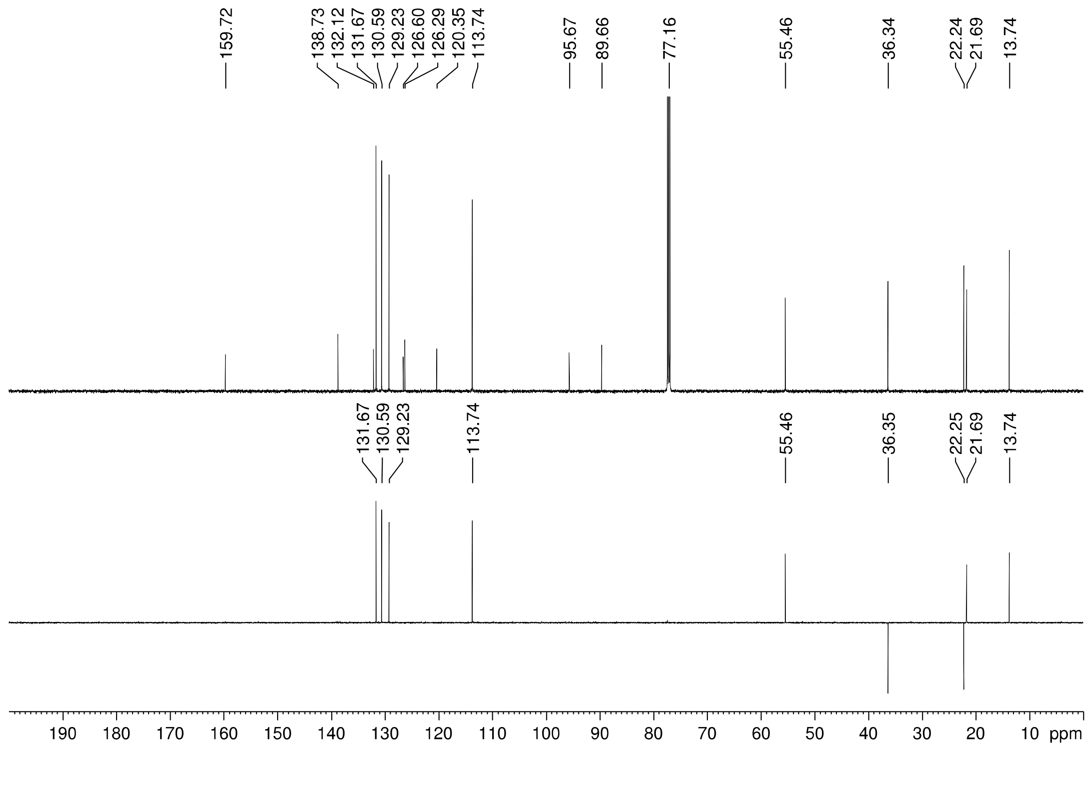
159.72
138.73
132.12
131.67
130.59
129.23
126.60
126.29
120.35
113.74
95.67
89.66
77.16
55.46
36.34
22.24
21.69
13.74
131.67
130.59
129.23
113.74
55.46
36.35
22.25
21.69
13.74
190 180 170 160 150 140 130 120 110 100 90 80 70 60 50 40 30 20 10 ppm

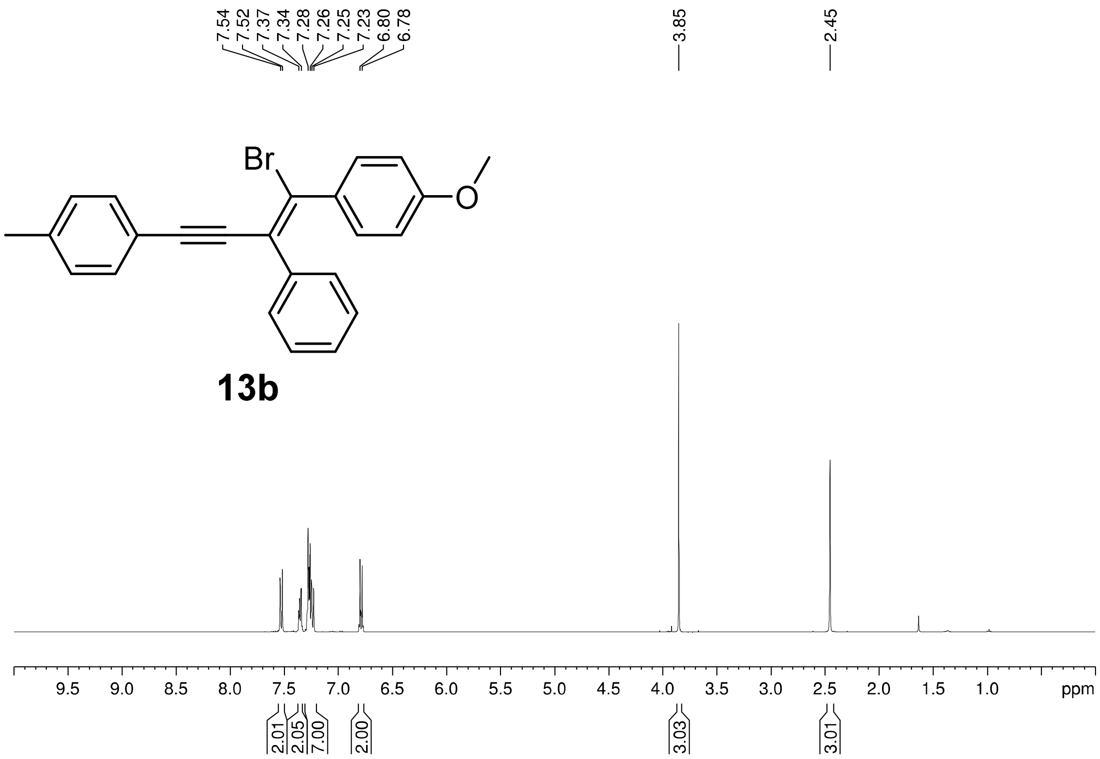
Br
O
7.54
7.52
7.37
7.34
7.28
7.26
7.25
7.23
6.80
6.78
3.85
2.45
9.5 9.0 8.5 8.0 7.5 7.0 6.5 6.0 5.5 5.0 4.5 4.0 3.5 3.0 2.5 2.0 1.5 1.0 ppm
2.01
2.05
7.00
2.00
3.03
3.01

13b

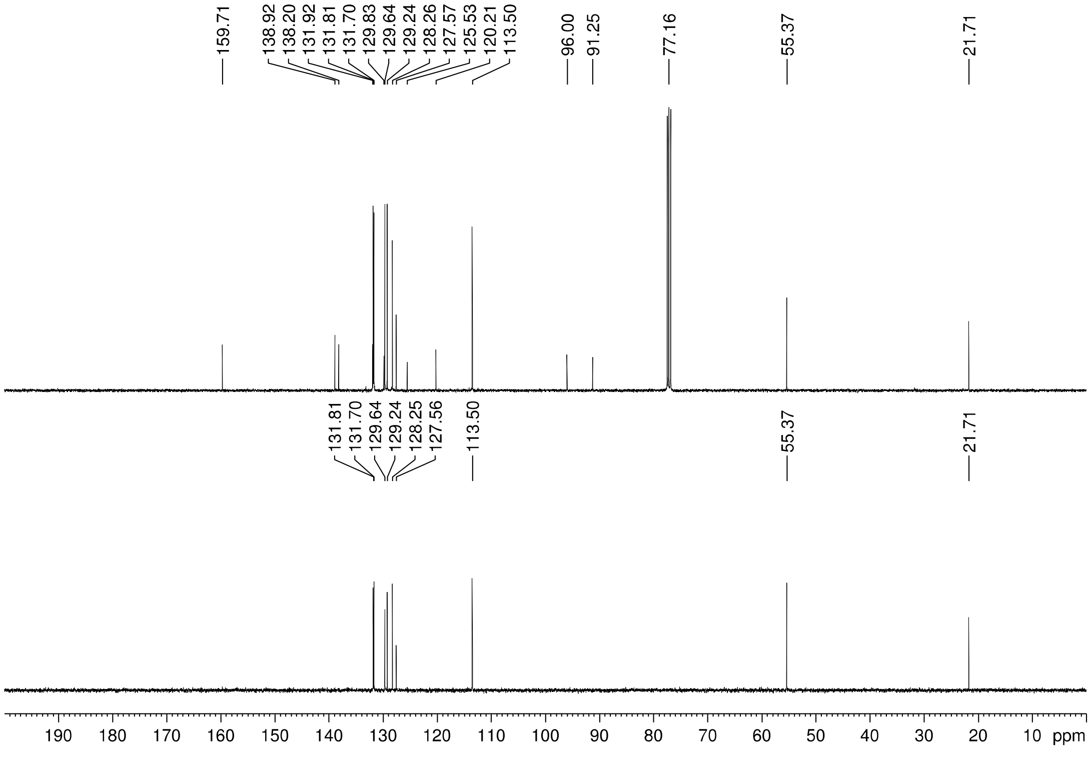
159.71
138.92
138.20
131.92
131.81
131.70
129.83
129.64
129.24
128.26
127.57
125.53
120.21
113.50
96.00
91.25
77.16
55.37
21.71
131.81
131.70
129.64
129.24
128.25
127.56
113.50
55.37
21.71
190 180 170 160 150 140 130 120 110 100 90 80 70 60 50 40 30 20 10 ppm

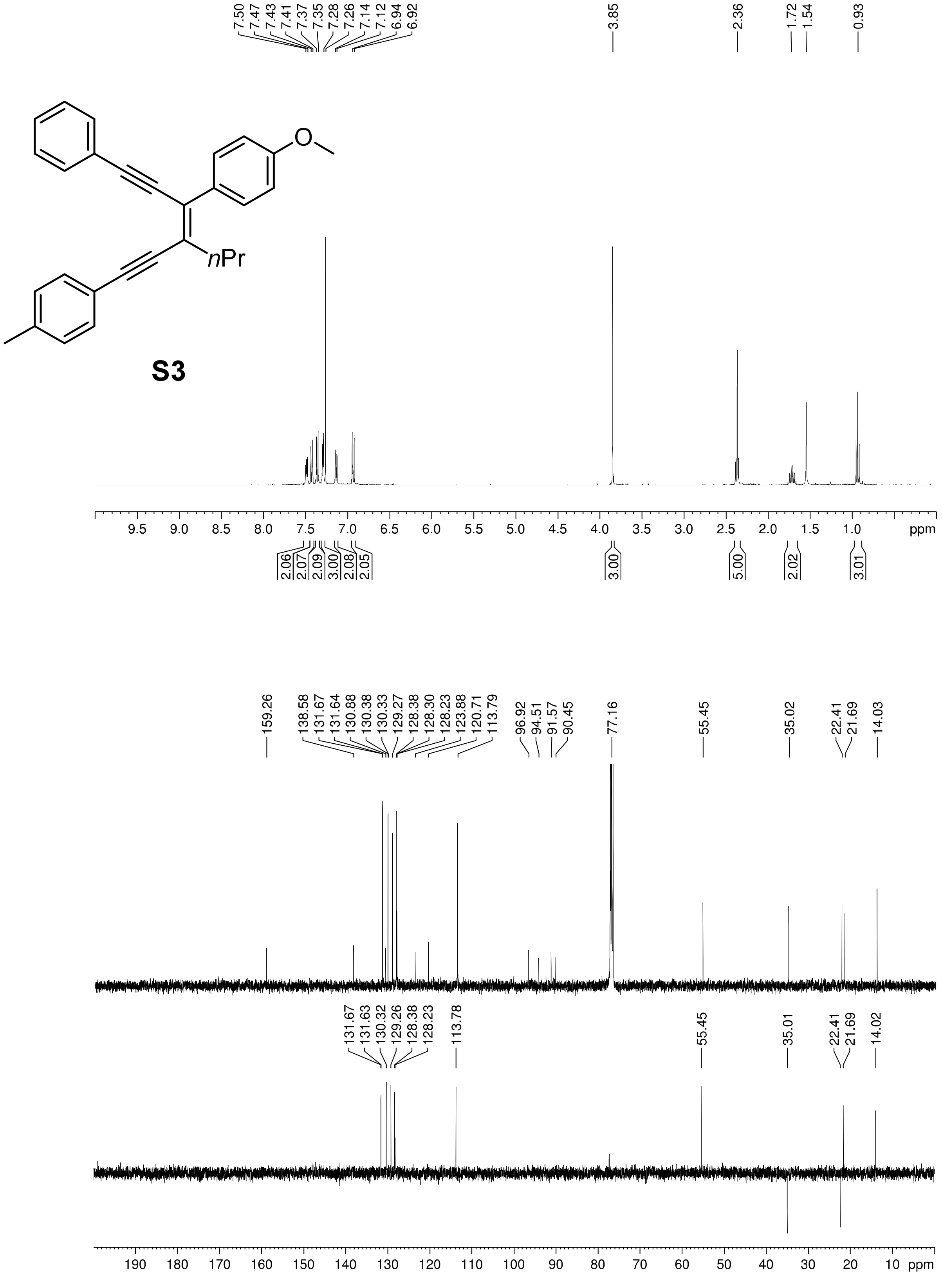

nPr
S3
7.50
7.47
7.43
7.41
7.37
7.35
7.28
7.26
7.14
7.12
6.94
6.92
3.85
2.36
1.72
1.54
0.93
ppm
2.06
2.07
2.09
3.00
2.08
2.05
3.00
5.00
2.02
3.01
159.26
138.58
131.67
131.64
130.88
130.38
130.33
129.27
128.38
128.30
128.23
123.88
120.71
113.79
96.92
94.51
91.57
90.45
77.16
55.45
35.02
22.41
21.69
14.03
131.67
131.63
130.32
129.26
128.38
128.23
113.78
55.45
35.01
22.41
21.69
14.02
ppm

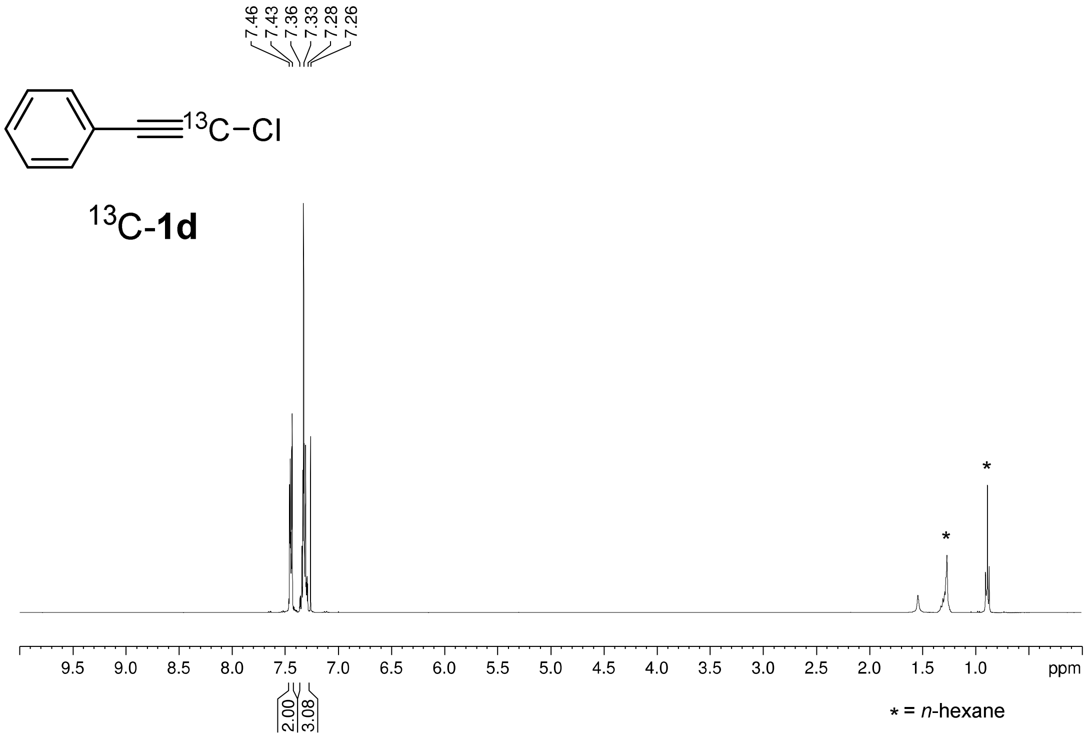
7.46
7.43
7.36
7.33
7.28
7.26
9.5 9.0 8.5 8.0 7.5 7.0 6.5 6.0 5.5 5.0 4.5 4.0 3.5 3.0 2.5 2.0 1.5 1.0 ppm
2.00
3.08
* = n-hexane

^{13}C-**1d**

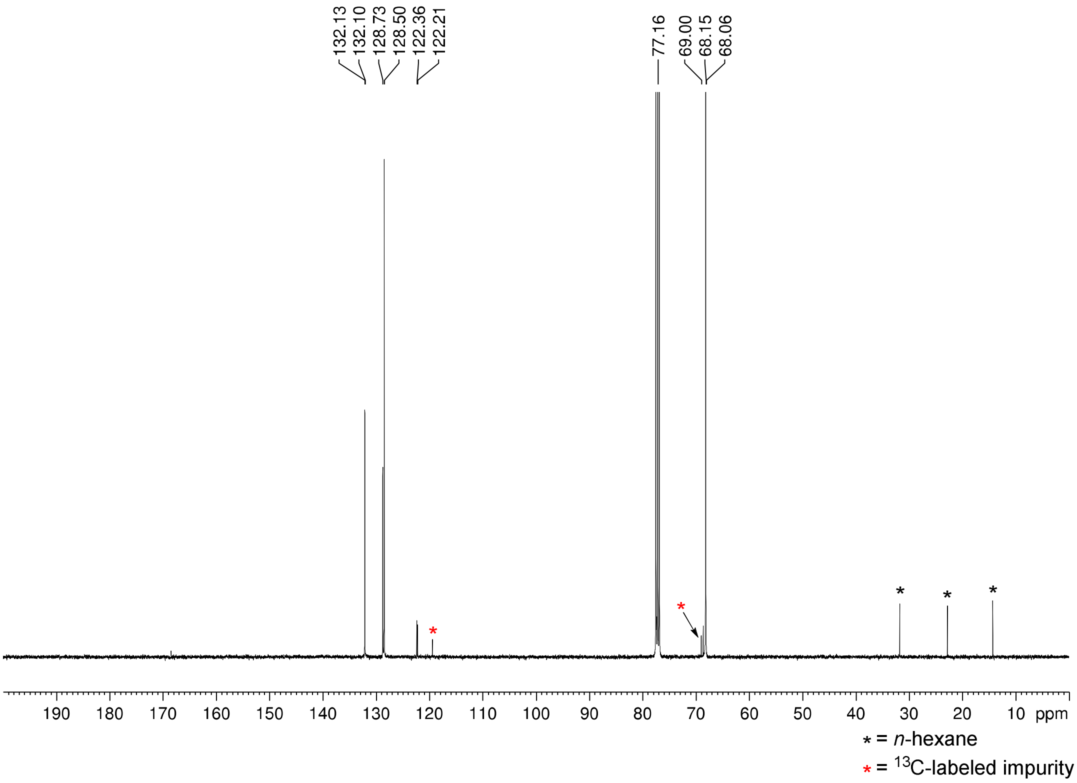
132.13
132.10
128.73
128.50
122.36
122.21
77.16
69.00
68.15
68.06
190 180 170 160 150 140 130 120 110 100 90 80 70 60 50 40 30 20 10 ppm
* = n-hexane
* = 13C-labeled impurity

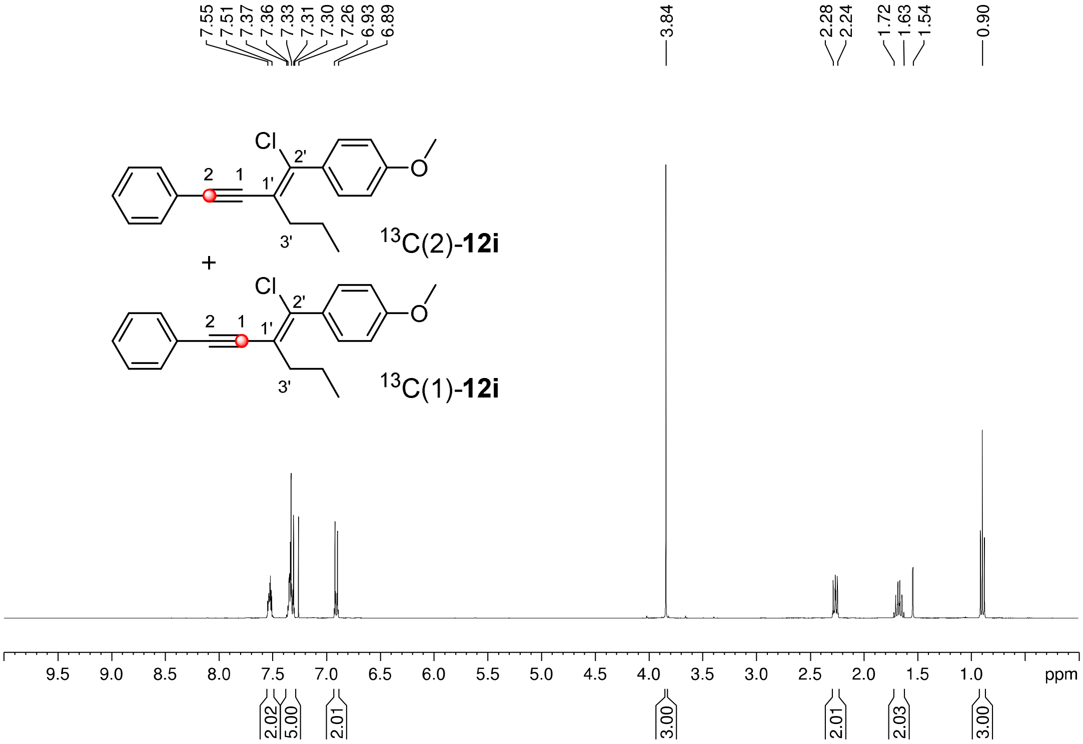
7.55
7.51
7.37
7.36
7.33
7.31
7.30
7.26
6.93
6.89
3.84
2.28
2.24
1.72
1.63
1.54
0.90
Cl
2'
2
1
1'
3'
O
^{13}C(2)-**12i**
+
^{13}C(1)-**12i**
9.5 9.0 8.5 8.0 7.5 7.0 6.5 6.0 5.5 5.0 4.5 4.0 3.5 3.0 2.5 2.0 1.5 1.0 ppm
2.02
5.00
2.01
3.00
2.01
2.03
3.00

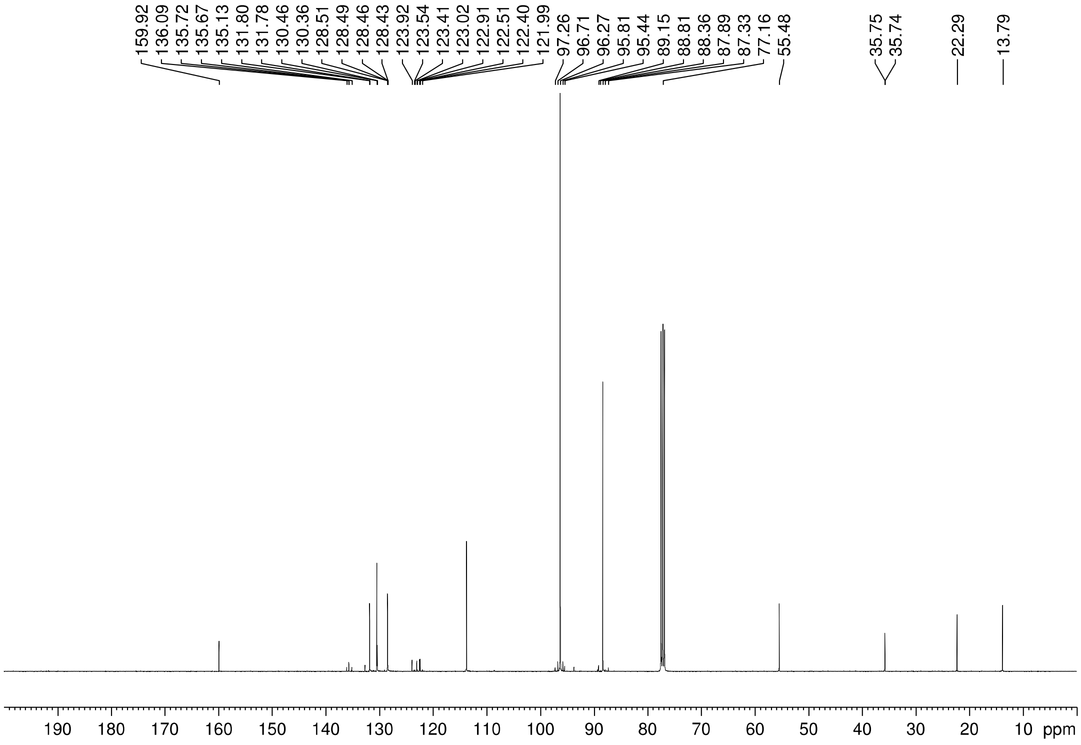
159.92
136.09
135.72
135.67
135.13
131.80
131.78
130.46
130.36
128.51
128.49
128.46
128.43
123.92
123.54
123.41
123.02
122.91
122.51
122.40
121.99
97.26
96.71
96.27
95.81
95.44
89.15
88.81
88.36
87.89
87.33
77.16
55.48
35.75
35.74
22.29
13.79
190 180 170 160 150 140 130 120 110 100 90 80 70 60 50 40 30 20 10 ppm

6. Supporting Information References

(1) Zhang, Z.; Luo, Y.; Du, H.; Xu, J.; Li, P. *Chem. Sci.* **2019**, *10*, 5156-5161.
(2) Hojo, M.; Murakami, Y.; Aihara, H.; Sakuragi, R.; Baba, Y.; Hosomi, A. *Angew. Chem., Int. Ed.* **2001**, *40*, 621-623.
(3) Wang, X.; Studer, A. *J. Am. Chem. Soc.* **2016**, *138*, 2977-2980.
(4) Shi, D.; Liu, Z.; Zhang, Z.; Shi, W.; Chen, H. *ChemCatChem* **2015**, *7*, 1424-1426.
(5) Chen, X.; Chen, D.; Lu, Z.; Kong, L.; Zhu, G. *J. Org. Chem.* **2011**, *76*, 6338-6343.
(6) Gulia, N.; Pigulski, B.; Charewicz, M.; Szafert, S. *Chem. Eur. J.* **2014**, *20*, 2746-2749.
(7) Zhao, Y.; Jin, J.; Chan, P. W. H. *Adv. Synth. Catal.* **2019**, *361*, 1313-1321.
(8) Chen, Z.; Jiang, H.; Li, Y.; Qi, C. *Chem. Commun.* **2010**, *46*, 8049-8051.
(9) Bai, Y.-B.; Luo, Z.; Wang, Y.; Gao, J.-M.; Zhang, L. *J. Am. Chem. Soc.* **2018**, *140*, 5860-5865.
(10) Gaussian 16, Revision A.03, Gaussian, Inc., 2016.
(11) Becke, A. D. *Phys. Rev. A* **1988**, *38*, 3098-3100.
(12) Lee, C.; Yang, W.; Parr, R. G. *Phys. Rev. B* **1988**, *37*, 785-789.
(13) Miehlich, B.; Savin, A.; Stoll, H.; Preuss, H. *Chem. Phys. Lett.* **1989**, *157*, 200-206.
(14) Grimme, S.; Ehrlich, S.; Goerigk, L. *J. Comp. Chem.* **2011**, *32*, 1456-1465.
(15) Ditchfield, R.; Hehre, W. J.; Pople, J. A. *J. Chem. Phys.* **1971**, *54*, 724-728.
(16) Hehre, W. J.; Ditchfield, R.; Pople, J. A. *J. Chem. Phys.* **1972**, *56*, 2257-2261.
(17) Weigend, F.; Ahlrichs, R. *Phys. Chem. Chem. Phys.* **2005**, *7*, 3297-3305.
(18) Andrae, D.; Häußermann, U.; Dolg, M.; Stoll, H.; Preuß, H. *Theor. Chim. Acta* **1990**, *77*, 123-141.
(19) Adamo, C.; Barone, V. *J. Chem. Phys.* **1999**, *110*, 6158-6170.
(20) Ernzerhof, M.; Scuseria, G. E. *J. Chem. Phys.* **1999**, *110*, 5029-5036.
(21) Zhao, Y.; Truhlar, D. G. *Theor. Chem. Account* **2008**, *120*, 215-241.
(22) Grimme, S. *J. Comput. Chem.* **2006**, *27*, 1787-1799.
(23) Grimme, S.; Antony, J.; Ehrlich, S.; Krieg, H. *J. Chem. Phys.* **2010**, *132*, 154104.
(24) Grimme, S. *Chem. Eur. J.* **2012**, *18*, 9955-9964.
(25) Figgen, D.; Rauhut, G.; Dolg, M.; Stoll, H. *Chem. Phys.* **2005**, *311*, 227-244.
(26) Peterson, K. A.; Puzzarini, C. *Theor. Chem. Acc.* **2005**, *114*, 283-296.
(27) Krishnan, R.; Binkley, J. S.; Seeger, R.; Pople, J. A. *J. Chem. Phys.* **1980**, *72*, 650-654.
(28) McLean, A. D.; Chandler, G. S. *J. Chem. Phys.* **1980**, *72*, 5639-5648.
(29) Curtiss, L. A.; McGrath, M. P.; Blaudeau, J. P.; Davis, N. E.; Binning Jr, R. C.; Radom, L. *J. Chem. Phys.* **1995**, *103*, 6104-6113.
(30) Clark, T.; Chandrasekhar, J.; Spitznagel, G. W.; Schleyer, P. V. R. *J. Comput. Chem.* **1983**, *4*, 294-301.
